# Implementation of expert systems to support the functional evaluation of stand-to-sit activity

**DOI:** 10.1186/1475-925X-13-98

**Published:** 2014-07-21

**Authors:** Maíra Junkes-Cunha, Glauco Cardozo, Christine F Boos, Fernando de Azevedo

**Affiliations:** 1 Medical Sciences Postgraduate Program, University Federal of Santa Catarina, Florianópolis, Santa Catarina, Brazil; 2 Institute of Biomedical Engineering, University Federal of Santa Catarina, Florianópolis, Santa Catarina, Brazil; 3 Electrical and Eletronics Engineering Department, University Federal of Santa Catarina, Florianópolis, Santa Catarina, Brazil

**Keywords:** Expert system, Physiotherapy, Activities of daily life, Disability evaluation

## Abstract

**Background:**

Functional evaluation of sit-to-stand and stand-to-sit activities is often used by physiotherapists in patients with neurological and musculoskeletal disorders. The observation of the way these activities are executed is essential in identifying kinesiological problems. There are different methodologies used to describe the stand-to-sit activity and its evaluation is not yet standardized, which makes the practical application of resources on clinical observation difficult. The objective of this study is to automate the decision making process of an evaluation protocol, developed in previous study, and facilitate its utilization by professionals in the area.

**Methods:**

A decision-making system has been implemented through a computational tool, more specifically an Expert System that due its inherent characteristics emulates the decision-making process of a human expert in the domain area. A *Shell* called Expert Sinta was used to develop two knowledge bases, i.e. two expert systems, one for the anterior view and another for the lateral view of stand-to-sit activity. Variables, values, associated rules and confidence factors, objectives, and additional information questions were defined by the expert of domain and once implemented each expert system generates a number of questions to its user. These questions serve as a guide to physiotherapists and support the standardization of the activity evaluation. The developed systems were evaluated by physiotherapists through the application of a questionnaire that evaluates the knowledge base and the usability of the system. The physiotherapists’ answers were then evaluated through statistical estimation and percentage analysis.

**Results:**

When asked about the systems’ “utility for clinical practice of the physiotherapist”, 67% of evaluators answered positively. An interesting finding was that most physiotherapists (i.e. 92%) considered that the systems are suitable for educational purposes, which was not the main objective of this study.

**Conclusions:**

The developed expert systems can support the physiotherapist in evaluating stand-to-sit activity through a conclusion suggestion about the “level of inadequacy” for the “degree of inadequacy” searched during its execution. Results of experts’ evaluation analyzed through statistical methods indicate that the automation of protocols contributed to the standardization of the evaluation of stand-to-sit activity and that it has application for teaching purposes.

## Background

Sit-to-stand and stand-to-sit are common movements in daily life [1]. Functional evaluation of these activities is often used by physiotherapists in patients with neurological and musculoskeletal disorders [2]. The observation of the way these activities are executed is essential in identifying kinesiological problems, being preconditions and post conditions for orthostatic mobility [3,4]. Although the stand-to-sit activity is performed several times a day, it has been less investigated because it has lower functional impact compared with the sit-to-stand activity [5].

Different methodologies are used to describe the stand-to-sit activity, and its evaluation is not standardized [6-10], which makes difficult the practical application on clinical observation. Kralj *et al*. [11] established a normative data for the stand-to-sit activity describing it in 4 phases: *initial phase* (anterior tilt of trunk), *descending* (vertical displacement), *seat loading* (weight transfer to the seat), and *stabilization* (trunk and balance adjustment). Perracini *et al*. [12] consider this activity to be performed in sequential order of the *anterior tilt* (phase I), *vertical displacement* (phase II), *angular displacement of knee* (phase III), and *stabilization* (phase IV).

Cunha *et al*. [13,14] developed two protocols to support the observation of the movements during the stand-to-sit activity on anterior and lateral views and described them in 4 consecutive phases: “Initial Position”, “Pre-squat”, “Squat” and “Stabilization”. For each phase, the protocol gives 3 options of answers. An example using the knee segment is shown in Table 1. The proposed methodology allows the observation of the sequential events and the respective phases during its execution, which is useful for clinical practice and research purposes. The proposal of using these protocols is to facilitate the analysis of the stand-to-sit activity in clinical practice [13,14] because there is no gold standard method for identifying the positions of body segments during its execution [3]. This would allow the identification of inappropriate movements to orientate the therapeutic decision making, considering that kinesiological abnormalities compromise the functionality of human beings. The practicality regarding the use of these protocols may attend to the physiotherapists’ daily requirements in the clinical practice. The automation would help to spread their use among physiotherapists and also to standardize these procedures.

**Table 1:** Example of description of stand-to-sit activity on the anterior view

**BODY SEGMENT**	**PHASE 1**	**PHASE 2**	**PHASE 3**	**PHASE 4**
	**Initial position**	**Pre-squat**	**Squat**	**Stabilization**
Right knee	Neutral position	Neutral position	Neutral position	Neutral position
	Valgus	Valgus	Valgus	Valgus
	Varus	Varus	Varus	Varus

Source: CUNHA *et al.*[13] and CUNHA [14].

Expert Systems (ESs) are among the most popular and promising products of the Artificial Intelligence (AI). They have been used to solve problems by trying to simulate human behavior of experts in a particular domain. They can be found in different medical specialties and in other health areas: psychiatry, for the identification of mental disorders [15]; cardiology, for diagnostic assistance in coronary artery disease [16]; endocrinology, for decision support by multiple regimen insulin dose adjustment [17]; neurology, for the diagnosis and identification of neurological disorders symptoms [18]; orthopedics, for foot abnormality recognition [19]; and pediatrics, for the diagnosis of pediatric rheumatic diseases [20]. In physiotherapy, studies about the development of ESs are focused on the analysis of risk factors at work-related musculoskeletal disorders [21], to support decision making in the treatment of shoulders and neck pain [22] and to help in low-back pain diagnosis [23].

Based on this context, the aim of this study is to automate the protocols developed in previous study [13,14] in a decision-making system based on an ES to support the physiotherapists in the evaluation of stand-to-sit activity.

## Methods

### Study design

This research is classified as technological and applied [24,25], was approved by the Ethics Committee on Human Research of University Federal of Santa Catarina (protocol number 1093) and qualitative techniques were used in the evaluation process.

### Instruments

The developed ESs could be implemented using either the Prolog language exploring all resources made available by the language or a *Shell* to facilitate the system’s implementation by people without expertise on computer systems. Considering the need for fast prototyping, the last option was chosen to allow the implementation of the system by the domain expert (physiotherapist researcher), the AI expert acting only as a consultant. *Shell* Expert Sinta (which can be found in Additional file 1) was chosen [26] because it uses a computational model based on production rules (If-Then) and confidence factors for structuring human knowledge. An example of some of the rules for the knee segment is shown in Table 2.

**Table 2:** Model of production rules of protocol to support the evaluation of stand-to-sit activity on LV

**RULE NUMBER**	**RULE**
1	if
	P1 R Knee == Neutral position
	then
	IP R Knee ← Neutral
2	if
	P1 R Knee <> Neutral position
	then
	IP R Knee ← Not neutral
3	if
	P1 == 0; P2 == 0; P3 == 0; P4 == 0
	then
	R Knee Condition ← "Adequate"
4	if
	P1 == 0; P2 == 0; P3 == 0; P4 == 1
	then
	R Knee Condition ← "Inadequate Level I"
5	if
	P1 == 0; P2 == 0; P3 == 1; P4 == 1
	then
	R Knee Condition ← "Inadequate Level II"
6	if
	P1 == 1; P2 == 1; P3 == 1; P4 == 0
	then
	R Knee Condition ← "Inadequate Level III"
7	if
	P1 == 1; P2 == 1; P3 == 1; P4 == 1
	then
	R Knee Condition ← "Inadequate Level IV"

The conclusions are shown based on their respective assumptions. For example, consider the rule 6. P1, P2, P3 and P4 mean the phases of the activity (as presented in the Table 1). The “0” value means the normal condition and value “1” means other than what is expected based on the literature. The rule conclusion (R Knee Condition ← “Inadequate Level III”) means that the knee condition has a level of inadequacy equal to III.

The instrument used for evaluation was a questionnaire based on ISO/IEC 9126–1 standard. For each view we used two protocols with two groups of questions: the first evaluates the knowledge base of the system and the second evaluates the usability of the system. The evaluative questions of knowledge base refer to “clarity of instructions”, “formulation of questions”, “order of evaluation and number of body segments”, “definition and sequence of stand-to-sit phases”, “interpretation of segmental evaluation” and “general conclusion of the results”. In this study a Likert type scale [27] was used with the following values: 1 for “Bad”, 2 for “Regular”, 3 for “Good” and 4 for “Excellent”. The usability questions of the system involve: “efficacy”, “utility for clinical practice of the physiotherapist”, “easy to use”, “supply the needs of physiotherapists in the evaluation of stand-to-sit activity”, “useful for educational purposes”, “dependency on the experience level in the area for ease of use”, and “reliability”. A dichotomous scale with value “1” for positive answers and “0” for negative answers was used. Additionally, there was a space for the evaluator to expose other relevant aspects they consider and that were not covered by the previous questions.

### Implementation of the expert systems

The systems were implemented according to the clinical needs of the physiotherapist, and the knowledge bases were elaborated according to the clinical protocols developed in previous studies [13,14] to support the evaluation of the stand-to-sit activity on the anterior (AV) and lateral (LV) views. As an example, the system for lateral view is available in Additional file 2. Developing a knowledge base using ES implies on defining variables, values, rules (production rules) and associated confidence factors (when necessary), objectives, and questions for a given problem. The variables were defined from established protocol according to the activity and related view in the evaluation, listing the various body segments and the different situations of stand-to-sit activity. For each variable it is necessary to define a set of values which are attributed to the variable during the execution of the ES. For example, on AV the variable “knee” has the values “neutral position”, *valgus* and *varus*.

In the next step, production rules were created as the following pseudo code:

if

Variable x == value *or* Variable x <> value

then

Variable y ← Value

Afterwards, the confident factors associated to each variable value in each production rule should be attributed. These values range from “0” (complete denial) to “1” (complete affirmation). They quantify the certainty that a domain expert has on the subject according to his or her knowledge and experience. In this work, the intrinsic characteristics of the problem lead to variable values with only two possible confidence factors (“0” or “1”), which means they are dichotomous.

The next step involves defining the goal-variables, which are targets of the inference machine. There are two types of goal-variable, the intermediate and the final one. For each body segment one intermediate goal-variable is defined during the system construction. Afterwards the final goal-variable – i.e. the “degree of inadequacy” which is what the expert searches – is determined according to the processing of the intermediate goal-variables (Table 3).

**Table 3:** Conclusion of the results about the segments during the activity

**Conclusion of body segments in the activity**	**Value for the intermediate goal-variables**
Adequate Condition	0 points
Inadequate Condition Level I	1 points
Inadequate Condition Level II	2 points
Inadequate Condition Level III	3 points
Inadequate Condition Level IV	4 points

Source: CUNHA *et al.*[13] and CUNHA [14].

Finally, the questions asked by the system to the user were created. Each question is linked to only one variable of the system. The answer chosen by the user is the value appointed to that variable.

The final conclusion after consulting the system is to achieve the ultimate goal variable which represents the conditions of body segments during the execution of the stand-to-sit activity based on the used protocols. Scores were attributed to the “degree of inadequacy”, which corresponds to the number of phases in which the segment showed a position different from what is expected. Thus, “0” represents an “adequate condition” when a person performs appropriate movements during the activity and “4” corresponds to an “inadequate condition level IV” which means that the person presents different movements from what is expected in all phases of the activity (Table 3).

### Using the expert system

Running ESs means the that physiotherapist answers the questions presented by the systems about each body segment according to his/her observation of the patient movement execution. While running, the user can ask the reason behind any question made by the ESs. Depending on how the system was implemented, the ES can show these reasons exactly as they were provided to the system or, as a default, it traces the production rules used to build the question.

The ES offers possible answers for each question, from which the user chooses the most appropriate one regarding the current status of the body segment under analysis. When questions and answers are finished, the ES gives possible results to this current analysis and this occurs for all body segments. At the end of the whole process, the ES summarizes the found results for each body segment in a sequential manner. All of these results are then combined by the system to generate a “level of inadequacy” (for the “degree of inadequacy” searched). Therefore, the use of ESs to support the evaluation of the stand-to-sit activity allows a conclusion to indicate the condition of the body segment through the “level of inadequacy”.

### Evaluation of the patient

To evaluate the stand-to-sit activity the patient should be with the proper attire, such as swimsuit or gym clothes, so that the body segments are properly seen during the execution of the movements. The physiotherapist asks the patient to execute the stand-to-sit activity and observes the movements of each body segment according to the 4 phases of the activity. The experiments have shown that physiotherapist should run the ES after the first visualization of the movements of his/her patient which reduces the possibility of not observing some of the body segments. This eventual problem can also be minimized by observing the video recording (Additional file 3), which is often carried out.

### Interpretation of evaluation results

From the use of the ES, physiotherapists can understand the conclusion of the results about the body segments through a classification related to the adequacy (or inadequacy considering we have called “degree of inadequacy” to the corresponded variable) of the movements in each phase of activity. The “level of inadequacy” (for the “degree of inadequacy” searched) corresponds to the number of phases in which the segment shows a different situation other than what is correct according to the literature.

### ESs assessment

Evaluation of the systems is necessary before allowing their use in clinical practice. However, this evaluation does not consider the *Shell* Expert Sinta used to implement the knowledge bases in this work because it was developed by others [26]. Therefore, it is related only to both ESs which implement the evaluation protocol of stand-to-sit activity.

The ESs were evaluated by 12 physiotherapists, all of them with experience in the area and who were finishing a specialization program in the Functional Reeducation of Movement and Posture area at Central Institute of Physiotherapy–Hospital of Clinics in Medicine Faculty University of Sao Paulo. These physiotherapists were from 8 universities from different regions of Brazil, which gives a significant diversity in their basic training and they had no previous knowledge about ESs.

### Statistical analysis

Considering the nature and volume of data we have used an estimation of the parameters considering 95% confidence in order to generalize the results. The evaluative questions about knowledge bases scores were analyzed by calculating the average and standard deviation (SD) of the values and usability questions were analyzed through percentage.

## Results

At the end of physiotherapist’s consultation, the system presents suggestions about the conclusion, which represents the classification of segment conditions during the execution of stand-to-sit activity (Table 3). ESs also allow the creation of a decision tree related to each view, showing the chain of accepted rules to a specific question. This tree can support the expert user in understanding the conclusion provided by the system by following the inference chain of rules accepted from the inference machine throughout consultation. The use of ES to evaluate the stand-to-sit activity allows a quantitative conclusion to indicate the condition of the body segment through the “level of inadequacy” (for the “degree of inadequacy” searched).

### ESs assessment

**Figure 1:**
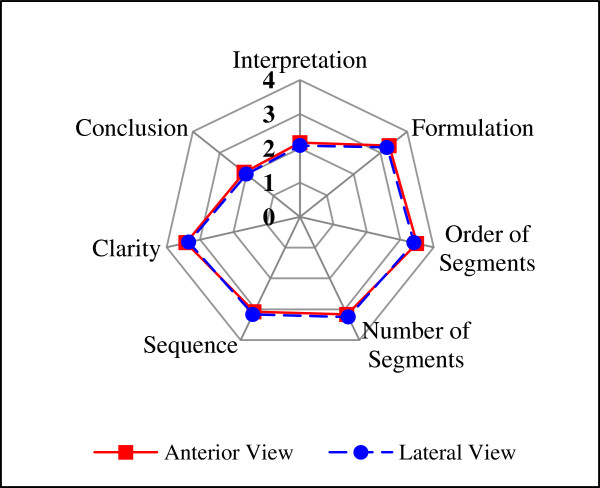
Comparison of average scores of evaluative items for both views.

**Figure 2:**
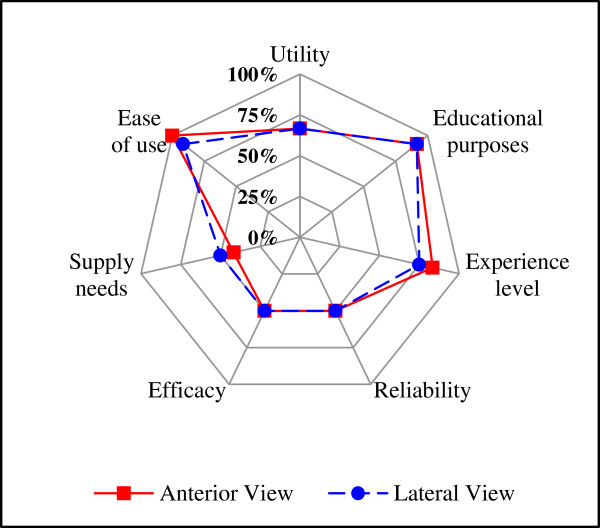
Comparison of evaluative percentages about usability of the system for both views.

The confidence interval for the average score of the knowledge bases items was from 2.76 to 3.10 for AV, and from 2.79 to 3.23 for the LV, as shown in Table 4. The average score of physiotherapists’ answers regarding evaluative items is shown in Table 5 and Figure 1, respectively. The average score of the answers regarding usability questions is shown in Table 6 and Figure 2, respectively. For AV the percentage of positive answers for usability was 69% with a confidence interval from 59.1% to 78.9%. For LV the percentage was 67.9% with a confidence interval from 57.9% to 77.9%.As observed in Figure 2 the “ease of use” was 100% for the system in LV while AV system 91.7% was achieved in this item. Most physiotherapists (i.e. 91.7%) considered that the systems are suitable for educational purposes. For LV, 75% of the evaluators agreed that there is correlation between ease of use and system-level experience in the specific area. In AV, 83.3% of them considered the existence of this correlation.

**Table 4:** Average score of knowledge base system

**View**	**Average ± SD**	**Confidence interval**
Anterior	2.96 ± 0.80	2.76 to 3.10
Lateral	2.93 ± 0.80	2.79 to 3.23

**Table 5:** Average score of evaluative items of knowledge bases system

**Items**	**Anterior view**	**Lateral view**
Clarity	3.4	3.3
Formulation	3.3	3.3
Order of Segments	3.5	3.4
Number of Segments	3.2	3.3
Sequence	3.1	3.2
Interpretation	2.2	2.1
Conclusion	2.1	2.0

**Table 6:** Percentage of the evaluative items of system usability

**Items**	**Anterior view**	**Lateral view**
**Efficacy**	50.0%	50.0%
**Utility**	66.7%	66.7%
**Ease of use**	100.0%	91.7%
**Supply needs**	41.7%	50.0%
**Educational purposes**	91.7%	91.7%
**Experience level**	83.3%	75.0%
**Reliability**	50.0%	50.0%

## Discussion

The results achieved with five from seven evaluative questions about the knowledge bases can be considered good, seeing as the average score achieved was 2.96 and 2.93 for AV and LV, respectively. An interesting finding was that the average score for practically all items coincide for both views.

Unlike the knowledge base assessment the usability questions have also presented relevant negative results. The percentages of positive answers for both views were respectively 69% and 67.9%. As happened in the evaluative questions there was an agreement between specialists on all items of the questionnaires concerning the usability of both views. Results show that specialists considered the system has good “ease of use”, “utility” and is suitable for educational purposes. According to specialists’ answers, these ESs can be considered applicable in providing a step-by-step sequence for the evaluation of the stand-to-sit activity, which can be a guide for physiotherapists without expertise in the area. In the case of specialized physiotherapists, the systems may provide a way of standardizing the evaluation.

However, half of the specialists found that the ESs cannot completely supply the needs of physiotherapists on supporting the evaluation, which indicates a lack of details about the movements in the activity. The evaluative item about “usability” presented the worst result. This clearly implies that while ESs may have clinical applicability there is a need for further studies in order to improve its general usability and usefulness for professionals in the area.

A possible improvement would be the creation of variables that could give more details about movements, for example, an intensity variable such as “range of motion”. However, this would require the development of a classification based on the degrees that occur in the body segments, which would increase the system complexity, as what is being considered for further studies.

The negative answers can be explained by the fact that many physiotherapists do not consider computational programs as useful tools in clinical practice. The majority of them use computational tools only for administrative activities (e.g. make spreadsheets and schedule patients). However, these answers showed that even physiotherapists who have “resistance” to the use of computer systems in clinical practice agreed that the ESs developed are user-friendly. Specialists who were more critical in their evaluation had somewhat unrealistic expectations that the system could automatically identify movement patterns through video recording.

Although the system has not been ranked as completely useful, in the free space for suggestions many evaluators reported the system’s application for teaching purposes. A future development necessary to allow this kind of use would be to build a tutorial system, preferably an intelligent one. Cardoso *et al.*[28] have developed an ES prototype of orthopedic hip, knee, and ankle exams by AI Expert Sinta *Shell*. Simulations performed on this prototype demonstrated that ESs can be used to support the systematization of these tests, which may contribute to support learning process of experts.

Finally, some reports of the specialists suggest that evaluators cannot trust a system that they do not understand the mechanisms of inference. It can be argued that: (I) the decision tree observation could give this confidence to the evaluators but they also were not able to understand the decision tree; (II) nowadays, in almost all areas of knowledge, people are dependents of results provided by machines and they do not know how these results were found (by the machine). In the health area, in particular, computer based medical equipments have played a very important role in the decision-making process and even so, they are not questioned, except in extraordinary cases. A systematic review study about controlled clinical trials to evaluate the effect of clinical decision support systems on physicians’ performance and clinical outcomes showed that these systems could improve the clinical performance for drug dosing, prevention, and other aspects related to medical care but were not convincing to support the diagnosis [29]. However, the goal of these ESs is to support the evaluation of the stand-to-sit activity without claiming to confirm the diagnosis [30]. In fact, a study about the development of an ES as a diagnostic support of cervical cancer found 100% effectiveness in the validation test, and it relies on the reduction of false positives and false negatives by providing a more accurate diagnosis for cervical cancer [31]. These results suggest that this system can support the professional to increase the accuracy of the evaluation of stand-to-sit activity.

## Conclusions

In this study the professional knowledge in physiotherapy has been transformed into a logical language and has been structured through the construction of production rules, used as basis for the implementation of the system.

The developed ESs can support the physiotherapist in evaluating stand-to-sit activity through a conclusion suggestion about the “level of inadequacy” for the “degree of inadequacy” searched during its execution. This process allows a quantitative result for the condition of the body segment. Results of experts’ evaluation analyzed through statistical methods indicate that the automation of protocols contributed to the standardization of the evaluation of stand-to-sit activity and that it has application for teaching purposes.

Further studies are needed in order to develop a completely useful system for physiotherapists’ clinical practice where it can be used as a reference for an overview of the segments in a subsequent functional evaluation.

## Abbreviations

AI: Artificial intelligence; CI: Computational intelligence; ESs: Expert systems; ES: Expert system; AV: Anterior view; LV: Lateral view.

## Competing interests

The authors declare that they have no competing interests.

## Authors’ contributions

MJ-C The author is a physiotherapist and did a study at the University of São Paulo during the course of specialization in which two protocols were developed for the assessment of stand-to-sit activity, one in anterior view and another in lateral view. In this study, the author was a domain expert and implemented these protocols during the Masters course at the Postgraduate Program in Medical Sciences at Federal University of Santa Catarina and was the main author for writing the manuscript. GC Being a computer specialist, the author supported the implementation. He contributed to the writing of the text about the implementation and in the revision of the text. CFB Being an telecommunications engineer, the author supported in the application of the statistical analysis and contributed to the writing and revision of the text. FdA This author is the head of the laboratory and guided the work of the thesis of Masters, which this paper refers. He contributed in all stages of this work as teacher and head of the group. All authors read and approved the final manuscript.

## Supplementary Material

Additional file 1 Stand-to-sit Expert System on Lateral View. Click here for file

Additional file 2 ***Shell *** **Expert Sinta.** Click here for file

Additional file 3 Consultation of the Expert System to support in the evaluation of stand-to-sit activity. Click here for file
