# Supplementary material for: Implementation of expert systems to support the functional evaluation of stand-to-sit activity
Source: Biomed Eng Online. 2014 Jul 21;13:98. doi: 10.1186/1475-925X-13-98 (PMC4120714; doi:10.1186/1475-925X-13-98)
Supplement: Additional file 2 — Shell Expert Sinta. [file 1475-925X-13-98-S2.zip › exvcldoc.zip!/SINTAVCL.rtf]

Laboratório de Inteligência Artificial/LIA
Grupo Sistemas INTeligentes Aplicados/SINTA
Universidade Federal do Ceará


Expert SINTA 
Visual Component Library
versão 1.1

Manual do Desenvolvedor


© 1995-1998	Laboratório de Inteligência Artificial/LIA	
	Grupo Sistemas INTeligentes Aplicados/SINTA	


Expert SINTA Visual Component Library
Versão da documentação 1.1
Criação deste documento: 11 de abril de 1997
Última modificação: 5 de janeiro de 1998


Apresentação

	
A construção de sistemas especialistas envolve investimentos em profissionais de diversas áreas, implicando em riscos maiores de produção. Uma conhecida estratégia para minimizar o problema é o investimento em ferramentas capazes de prototipar, avaliar e implementar o projeto de um sistema. Procura-se, assim, diminuir a quantidade de recursos necessários, entre eles o tempo envolvido. Porém, é difícil conciliar alta produtividade e versatilidade em ferramentas qualificadas em tais categorias.
	O Expert SINTA, um shell voltado para facilidade de uso, já vem sendo utilizado com sucesso na construção de sistemas especialistas, mas não dispõe de recursos de intercâmbio de dados com outros ambientes de desenvolvimento, nem possibilita o total aproveitamento das características do sistema operacional na criação de interfaces com o usuário final.
	Com a introdução da biblioteca de componentes Expert SINTA Visual Component Library (Expert SINTA VCL), torna-se viável aproveitar as possibilidades oferecidas pela ferramenta descrita dentro de uma linguagem de programação orientada a objeto de alta produtividade. Ferramentas como estas são comumente denominadas ferramentas RAD (Rapid Application Development). Entre os motivos que levaram o grupo à implementação da Expert SINTA VCL, temos:

•	o Expert SINTA (shell) não provia toda a funcionalidade necessitada em certos sistemas especialistas;

•	não haviam meios de aproveitar os dados obtidos com o shell em outros programas;

•	seria inviável o acréscimo de vários recursos de interface e intercâmbio de dados na ferramenta em si. A política do grupo SINTA é disponibilizar soluções baseadas em sistemas inteligentes de forma gratuita para a comunidade de Computação em geral. Tal abordagem demandaria muito tempo e dinheiro;

•	os sistemas especialistas poderiam ser compilados em uma dada linguagem de programação e utilizados de forma totalmente independente do Expert SINTA;

•	seria possível reaproveitar milhares de linhas de código já escritas na construção do shell.

	O shell Expert SINTA destina-se às plataformas Windows 3.1 ou superior, incluindo versões nativas para Windows 95 e NT. A Expert SINTA Visual Component Library pode ser utilizada pelos ambientes Borland Delphi (ou C++ Builder) em qualquer versão.
Esse documento descreve as propriedades chave dos componentes da Expert SINTA Visual Component Library 1.1 e exemplos de aplicações. Para maiores informações sobre outras propriedades, métodos e eventos, consulte a documentação da classe base de cada componente apresentado na documentação respectiva do Borland Delphi.
Este documento de referência está organizado nas seguintes seções:

•	Desenvolvendo com a Expert SINTA VCL: aqui são explicados exemplos reais de aplicações implementadas com esta biblioteca. São apresentados os componentes de forma introdutória  seguidos de diferentes programas;

•	Além da Expert SINTA VCL: como estender a biblioteca e acrescentar novos componentes;
 
•	Manual de referência: descrição das principais propriedades, métodos e eventos de todos os componentes da Expert SINTA VCL. Abrange desde as classes de estrutura de dados aos componentes visuais. Consulte-o para conhecer outras funções e tirar dúvidas;

•	Sobre o projeto: informações sobre o LIA e os participantes da equipe de desenvolvimento do Expert SINTA e Expert SINTA VCL.

Para informações adicionais, consulte a documentação do shell Expert SINTA e do Borland Delphi.

	ATENÇÃO: O LABORATÓRIO DE INTELIGÊNCIA ARTIFICIAL (LIA) DISPÕE DE UMA HOMEPAGE PARA ESCLARECIMENTO DE DÚVIDAS: http://www.lia.ufc.br. PORÉM, NENHUMA GARANTIA É DADA PELOS AUTORES. DO MESMO MODO, SUPORTE TÉCNICO PODE NÃO ESTAR DISPONÍVEL.


Desenvolvendo com a 
Expert SINTA VCL

Como e onde usar determinados componentes? Esta seção explica desde a descrição das principais classes que formam a Expert SINTA VCL a exemplos úteis de como estas devem ser aplicadas.

Capítulo 1 - Os Componentes
Apresentando os onze componentes visuais da Expert SINTA VCL, suas funções e como estas se relacionam.

Capítulo 2 - Um Run-Time para o Expert SINTA
O primeiro exemplo prático mostra como construir rapidamente um run-time para efetuar consultas a bases de conhecimento geradas como o shell Expert SINTA.

Capítulo 3 - Controlando a Entrada de Dados
Organize a entrada de dados do usuário da maneira que lhe for conveniente. Neste capítulo, temos um pequeno formulário que permite a entrada prévia de dados para uma posterior consulta.

Capítulo 4 - A Expert SINTA VCL e Bancos de Dados
Bancos de dados são extremamente úteis para consultas a dados previamente cadastrados. Veja como relacionar um sistema especialista Expert SINTA a um banco de dados utilizando o Delphi.

Capítulo
1


Os Componentes


Quais são os componentes da Expert SINTA VCL? Como se relacionam? Onde utilizá-los? Estas perguntas são respondidas no capítulo que se segue, apresentando os onze componentes básicos da biblioteca. Os tópicos deste capítulo incluem:

•	O que é possível fazer com a Expert SINTA VCL
•	Os componentes
•	Relacionando os componentes

O que é possível fazer com a Expert SINTA VCL

A Expert SINTA VCL é uma biblioteca de componentes para programação de sistemas especialistas baseados em regras de produção, fatores de confiança e encadeamento para trás, de forma semelhante ao clássico MYCIN. Para maiores informações sobre este tipo de sistema especialista, consulte a documentação do shell Expert SINTA.
De uma forma geral, a Expert SINTA VCL torna possível a criação de front-ends para bases de conhecimento geradas com o Expert SINTA. Entre as tarefas desempenhadas por esta VCL, temos:

•	encapsular a máquina de inferência e a estrutura de dados que representa o conhecimento (regras de produção);
	
•	fornecer mecanismos para entrada de dados do usuário;
	
•	fornecer mecanismos de depuração;
	
•	além disso, permitir personalização da aplicação final;

	Talvez o melhor exemplo da capacidade da Expert SINTA VCL seja o próprio shell Expert SINTA. Este basicamente acrescenta um editor de bases de conhecimento à biblioteca, onde todas as estruturas de dados, operações de consultas e elementos de interface gráfica estão implementadas. A partir da VCL, o único limite é a vontade do programador.

Os componentes

Antes de mais nada, proceda com a instalação da biblioteca no Borland Delphi. O arquivo de instalação é ExSystem.pas. Para maiores informações de como instalar componentes, consulte a documentação do Borland Delphi.
	Uma vez instalada, a barra de ferramentas a seguir deve aparecer em seu ambiente:


Figura 1 - A Barra de Ferramentas no Borland Delphi
	Os componentes nativos da Expert SINTA VCL podem ser divididos nas seguintes categorias:

Componente principal

	TExpertSystem
Este componente encapsula a máquina de inferência e a estrutura de dados que representa a base de conhecimento. Para tal, referencia um arquivo de base de conhecimento gerado pelo shell. Todos os outros componentes fazem referência a um dado componente TExpertSystem direta ou indiretamente.	

Componentes gerais de interface gráfica

	TRuleView
Exibe as regras de um base de conhecimento referenciada por um componente TExpertSystem.
	
	TExpertPrompt
Um menu para entrada de dados do usuário em resposta a uma determinada pergunta efetuada pelo sistema. Uma pergunta não é a única maneira que um sistema especialista utilizar para obter informações complementares, mas é a mais comum. Portanto, este componente é bastante utilizado.
	
	TLabelQuestion
A única opção de personalização de interface integrada no shell Expert SINTA é a possibilidade de mudança da mensagem que aparece em uma pergunta para cada variável. É possível reaproveitar esta mensagem dentro de front-ends montados em uma linguagem como o Borland Delphi, de forma a manter um aspecto uniforme entre uma consulta realizada no próprio shell e um aplicativo final construído com a VCL.
	
	TValuesGrid
Exibe as instâncias (valores) de uma dada variável por ordem decrescente de grau de confiança.	

Componentes de depuração

	TWhyDialog
Caixa de diálogo que exibe uma explicação para a necessidade de uma dada pergunta, baseando-se tanto em explicações criadas pelo projetista da base no shell ou, na falta destas, em explicações montadas automaticamente a partir das regras.
	
	TDebugPanel
Semelhante a TRuleView, exibe as regras da base de conhecimento de um sistema especialista em um painel, mas indica também qual premissa (ou conclusão) está sendo analisada pela máquina de inferência em determinado ponto de uma consulta.
	
	TWatchPanel
De forma semelhante a opção Watch de um ambiente de programação, exibe as instâncias (valores atribuídos durante uma consulta) de todas variáveis através de dois painéis: o superior lista todas as variáveis; o inferior, as instâncias da variável selecionada no painel superior.
	
	TConsultTree
Um sistema especialista precisa explicar porque (como) determinadas conclusões foram atingidas. Este componente pode criar e exibir de forma hierárquica todos passos seguidos do começo ao fim de uma consulta.
	
	TAllVars
Mais um componente de exibição de instâncias de variáveis? Sim, mas de forma hierárquica. Ao contrário de TWatchPanel, este componente são se atualiza automaticamente para cada nova instância criada pela máquina de inferência.	

Componente de navegação

	TExNavigator
É basicamente um navegador que controla o fluxo da consulta em conjunto com as respostas entradas pelo usuário e outros componentes de interface acrescentados pelo desenvolvedor da aplicação. Suas operações básicas são iniciar consulta, voltar à pergunta anterior, dar uma pausa na consulta, executá-la passo a passo, e cancelar a consulta.	

Relacionando os componentes

Agora que temos conhecimento das partes, precisamos compreender o todo. Como proceder para unir estes componentes em uma aplicação completa?
Existe outra forma de classificar a VCL: componentes de atualização automática, os quais modificam-se automaticamente sempre que um fato relevante ocorre durante uma consulta; componentes passivos, que precisam da chamada de um método para exibir funcionalidade.
Basicamente, todos os componentes, à exceção de TConsultTree e TAllVars, são automáticos. TValuesGrid pode ser automático ou passivo, mas normalmente é passivo.
Para que componentes automáticos procedam como tal, é preciso relacioná-los a um componente TExpertSystem. Para tal, existe a propriedade ExpertSystem. Através do Object Inspector, atribua um sistema especialista para cada controle automático. Por exemplo, crie um novo formulário e acrescente dois componentes: um TExpertSystem e um TRuleView.


Figura 2 - Um exemplo simples de relação entre componentes
	Os nomes default são ExpertSystem1 e RuleView1. Selecione RuleView1 e ative o Object Inspector. Clique na propriedade ExpertSystem e selecione ExpertSystem1. Nada parece ocorrer. Agora selecione ExpertSystem1 e, através do Object Inspector, digite na propriedade FileName o caminho de um determinado arquivo BCM de base de conhecimento. Dentro de ExpertSystem1 ocorrerá o carregamento das regras. Automaticamente, RuleView1 refletirá a mudança.
	Cada componente reage de acordo com a mudança feita. TRuleView reage a mudanças do arquivo da base de conhecimento, mas nenhum componente nativo da Expert SINTA VCL é notificado sobre mudanças realizadas diretamente na estrutura de dados, como, por exemplo, uma alteração de nome de variável feita através do shell. Seria caro demais em termos de processamento.
	A tabela a seguir indica os componentes e as respectivas operações de TExpertSystem que provocam alterações. Obviamente, TExpertSystem não entra na relação:

Componente	Eventos	
TRuleView	Carregamento de nova base de conhecimento.	
TExpertPrompt	Carregamento de nova base de conhecimento.	
TLabelQuestion	Carregamento de nova base de conhecimento.	
TValuesGrid	Carregamento de nova base de conhecimento. Quando a propriedade AutomaticUpdate é verdadeira, também sofre mudanças a cada instância que é acrescentada ou retirada da base de fatos do sistema especialista.	
TWhyDialog	A cada pergunta realizada.	
TDebugPanel	Carregamento de nova base de conhecimento e a cada passo efetuado pela máquina de inferência.	
TWatchPanel	Carregamento de nova base de conhecimento e cada instância modificada de cada variável.	
TConsultTree	Não é atualizado automaticamente. Invoque um método para gerar mudanças.	
TAllVars	Não é atualizado automaticamente. Invoque um método para gerar mudanças.	
TExNavigator	Carregamento de nova base de conhecimento, início e fim de consulta, entrada e saída em modo de execução passo-a-passo, retorno à pergunta anterior, nova pergunta realizada. 	

	Outro parâmetro importante que aparece constantemente nos componentes da Expert SINTA VCL são os códigos de variáveis. Por exemplo, o componente TExpertPrompt monta automaticamente um menu de entrada de dados para que o usuário marque valores de uma dada variável. Logo, a variável é um parâmetro básico deste componente. Você indica a variável referenciada pelo componente através de seu código.
	Cada variável criada através do Expert SINTA recebe um código interno que nunca muda (a não ser, óbvio, que esta seja apagada e inserida novamente - categoricamente nem sequer seria a mesma variável). Assim, o uso de códigos é o modo mais estável de referência de variáveis. Nomes podem ser muito compridos e mudam constantemente.
	Para  obter os códigos criados pelo Expert SINTA, abra a base de conhecimento no shell e selecione o menu Arquivo  Exportar  Códigos... Digite o nome do arquivo texto onde sairão os resultados. Abra posteriormente este arquivo no seu editor de textos favorito quando precisar saber o código de uma dada variável ou valor.
	Sobre a entrada do nome do arquivo da base de conhecimento no Object Inspector, referente à propriedade FileName de TExpertSystem, é bom que se lembre que estamos digitando um nome de arquivo como outro qualquer. Assim, se o arquivo está em outro diretório que não o mesmo dos arquivos fonte da sua aplicação em desenvolvimento e diretório de saída, deve-se entrar em FileName o caminho completo ou relativo. Se você mantiver o mesmo diretório para base de conhecimento, arquivos fonte e arquivos objeto/executável, basta que se entre o nome da base, como "secaju.bcm" (isto é feito nos exemplos incluídos com a Expert SINTA VCL).
	Este detalhe pode parecer um contratempo, mas para uma biblioteca simples como a Expert SINTA VCL é uma alternativa mais atraente que o uso de mecanismos de independência de diretório, como o uso de um alias, adotado na Borland Database Engine. De qualquer forma, com uma única linha de código é possível consertar qualquer eventual dificuldade:

procedure TForm1.FormCreate(Sender: TObject);
begin
   ExpertSystem.FileName := 'meu sistema.bcm';
end;


Capítulo
2


Um Run-Time para o Expert SINTA


Este é o primeiro de uma série de exemplos para demonstrar a funcionalidade da Expert SINTA VCL. O código completo dos mesmos acompanha a biblioteca. Neste capítulo, o exemplo é um run-time, um programa capaz de aproveitar arquivos gerados em outro mas sem permitir edição. No nosso caso, isto significa a capacidade de realizar consultas. Basicamente, eqüivale ao shell Expert SINTA, mas sem as capacidades de edição.

Na linha de montagem

Os formulários principais de nosso run-time são dois: frmMain, a janela onde ocorrem as consultas e frmResults, onde os resultados são exibidos.
	Em frmMain, os principais objetos encontrados são:

•	LabelQuestion (classe TLabelQuestion): para exibição de perguntas;
•	ExpertPrompt (classe TExpertPrompt): para permitir a entrada de dados do usuário;
•	btnOk (classe TBitBtn): botão para confirmar entradas do usuário;
•	btnWhy (classe TBitBtn): chama caixa de diálogo com explicações;
•	WhyDialog (classe TWhyDialog): a própria caixa de diálogo;
•	DebugPanel (classe TDebugPanel): exibe regras e indica o ponto de análise da máquina de inferência durante uma consulta;
•	WatchPanel (classe TWatchPanel): painéis para exibição de instâncias de variáveis. Observe que o mesmo se encontra junto com DebugPanel dentro de um objeto TNotebook. Para visualizar WatchPanel, clique na lista do Object Inspector;
•	ExNavigator (classe TExNavigator): navegador que controlará a consulta;
•	ExpertSystem (classe TExpertSystem): o componente principal que tudo centraliza;

	Os componentes são unidos através da propriedade ExpertSystem. A base de conhecimento do sistema especialista será indicada em tempo de execução através da caixa de diálogo OpenDialog. O código a seguir mostra o momento que um arquivo foi escolhido e deve ser carregado no sistema:

//Procedimento: TfrmMain.mnuOpenClick(Sender: TObject);
ExpertSystem.FileName := OpenDialog.FileName; 

	Observe que não houve preocupação com a propriedade VarCode do objeto ExpertPrompt, mas ela é vital. É a propriedade que indica o código da variável (lembre o último capítulo) cujo menu de entrada será construído. Vamos atualizar este valor em tempo de execução.
	Como é comum em componentes, as classes da Expert SINTA VCL possuem diversos eventos. Um dos eventos vitais é OnPrompt, da classe TExpertSystem. Este evento é disparado sempre que o sistema necessita de informações sobre os valores de uma determinada variável, variável esta que não pode ser deduzida de nenhuma regra. A forma mais comum é através de um componente TExpertPrompt (no capítulo 4 veremos outra). A definição de  OnPrompt é:

OnPrompt = TVarBasedEvent;

	TVarBasedEvent é um evento de dois parâmetros: "Sender", o objeto que invocou o evento (como nos demais componentes do Delphi) e "V", a variável necessária cujo significado varia de acordo com o contexto. No caso de OnPrompt, indica  a variável da qual desejamos obter informações. Utilizamos "V" no nosso run-time no seguinte modo:

procedure TfrmMain.ExpertSystemPrompt(Sender: TObject; V: Integer);
begin
   LabelQuestion.VarCode := V;
   ExpertPrompt.VarCode := V;
end;

	Utiliza-se "V" para atualizar LabelQuestion e ExpertPrompt. O evento OnPrompt também paralisa (automaticamente) a consulta, modificando internamente o valor da propriedade WaitingAnswer, de TExpertSystem. A consulta não prossegue enquanto WaitingAnswer for verdadeira. Cabe ao programador do aplicativo decidir quando a resposta foi dada para que a máquina de inferência prossiga.
	No nosso run-time, isto ocorre através do botão OK. Clicar em OK equivale a confirmar a entrada. Logo, o seguinte código deve ser acrescentado:

procedure TfrmMain.btnOKClick(Sender: TObject);
begin
   ExpertPrompt.UpdateBase;
   ExpertSystem.WaitingAnswer := false;
end;

	O método UpdateBase de TExpertPrompt lê a(s) entrada(s) marcada(s) e atualiza as instâncias da variável relacionada ao objeto através da propriedade VarCode. Após esta atualização, a máquina de inferência é desbloqueada (WaitingAnswer := false).

A hora do resultado

Agora devemos nos preocupar com a exibição de resultados. Observe que frmResults é exatamente o mesmo formulário utilizado no shell Expert SINTA. Ele consiste de quatro componentes principais, dispostos em um objeto TNotebook:

•	PanelVarName (classe TPanel): exibirá o nome da variável objetivo em questão.
•	ValuesGrid (classe TValuesGrid): irá exibir as instâncias da variável objetivo. Observe que a propriedade AutomaticUpdate foi assinalada como falsa para diminuir o processamento realizado pelo sistema especialista;
•	ConsultTree (classe TConsultTree): irá exibir todos os passos feitos durante a consulta;
•	AllVars (classe TAllVars): árvore com as instâncias de todas as variáveis;
•	RuleView (classe TRuleView): novamente, um objeto para exibição de regras. É importante deixar um objeto deste tipo acessível para melhor compreensão da árvore exibida em ConsultTree;

	À exceção de RuleView, todos os demais objetos precisam ser atualizados através da chamada de um método. O evento ideal para isto é OnShowResults de TExpertSystem. OnShowResults também é do tipo TVarBasedEvent, o que significa que um de seus parâmetros é uma variável. Esta é a variável objetivo do momento (pode haver outras, dependendo do sistema). O código de OnShowResults em nosso exemplo é:

procedure TfrmMain.ExpertSystemShowResults(Sender: TObject; V: Integer);
begin
   with frmResults do begin
      PanelVarName.Caption := ExpertSystem.VarName(v);
      ValuesGrid.VarCode := v;
      ValuesGrid.RefreshValues;
      ConsultTree.CreateTree(ExpertSystem);
      AllVars.CreateTree(ExpertSystem);
      Notebook.PageIndex := 0;
      TabSet.TabIndex := 0;
      ShowModal;
   end;
end;

	PanelVarName é um objeto de frmResults que exibirá o nome de uma dada variável, no caso "V". A propriedade VarCode de ValuesGrid também recebe "V", e um método complementar, RefreshValues, deve ser invocado. ConsultTree e AllVars invocam o método CreateTree utilizando o sistema especialista ExpertSystem como parâmetro.
	Como última observação, no evento OnClose do formulário principal, frmMain, temos a seguinte linha de código:

ExpertSystem.AbortConsultation;

	Este comando aborta qualquer consulta que esteja em andamento. Caso contrário, a janela só fechará ao término da consulta.
	Diversas outras funções são realizadas neste exemplo. Pode-se citar a inibição ou bloqueio de elementos de interface no começo e no fim de uma consulta, a chamada de um arquivo de ajuda a partir da janela de resultados, entre outras. Para maiores detalhes, consulte o código completo do Expert SINTA RunTime e a Parte III deste manual.


Capítulo
3


Controlando a Entrada de Dados


	Nem só de aquisição interativa de informações comandada através do encadeamento para trás vive a máquina de inferência da Expert SINTA VCL. Neste capítulo curto, veremos outra alternativa de entrada de dados, provando-se que existem diversas possibilidades de aplicação da biblioteca. Cabe ao programador arranjá-las como achar melhor. E nada melhor como uma boa carta de vinhos para estimulá-lo!

Vinhos para uma refeição

Imagine-se à mesa em um restaurante futurista. À sua frente, uma tela espera ordens para recomendar um vinho de acordo com a refeição planejada. Marcando comodamente alguns itens, seu ajudante cibernético decide de imediato a melhor escolha.
	Neste exemplo, nada seria mais irritante que responder cada questão em separado, como normalmente ocorre em uma consulta conduzida pelo shell Expert SINTA. Mas não estamos mais no shell, há uma linguagem de programação toda à nossa disposição, domesticada pela presença da Expert SINTA VCL. Vinhos para uma refeição é um sistema especialista de demonstração, não devendo ser comparado a especialistas profissionais, mas pode lhe dar boas idéias de como montar uma aplicação final.

Na linha de montagem

O formulário onde a consulta ocorre deve dispor elementos de entrada de dados. Como são diversas as variáveis, aqui optou-se por dividi-las em grupos afins, formando uma espécie de ajudante passo-a-passo comum em programas Windows.
	Para cada variável de entrada, foi colocado um objeto TExpertPrompt com o respectivo código de variável, bem como um TLabelQuestion. Note que objetos TLabelQuestion bem que poderiam ser substituídos por labels comuns, mas usar TLabelQuestion traz a vantagem de reaproveitar o que foi feito no shell, permitindo que mudanças feitas na base reflitam tanto em uma consulta feita no shell como nesta aplicação.
	A única exceção foi  feita para a pergunta referente ao tipo de molho encontrado na refeição. Tínhamos duas opções: como a base original já dividia a questão do molho em duas variáveis (tem molho e tipo de molho), não seria desejável mudar. O fato de não ter molho necessariamente implica que não devemos perguntar mais sobre o tipo do mesmo. Logo, poderíamos deixar a pergunta sobre o tipo de molho fora do formulário principal e invocá-la somente se necessária, ao estilo do shell, ou disponibilizá-la de imediato e lembrar que não é necessária uma resposta caso a refeição não tenha molho. É o que ocorre em formulários do nosso cotidiano.
	Neste caso, a mensagem que aparece na aplicação é diferente daquele que aparece no shell. Como no shell a pergunta só é feita se necessária, ela pode (e deve) permanecer como "Que tipo de molho é usado na refeição?". Mas na aplicação, deve ser algo como "Em caso afirmativo, qual molho?". Observe com atenção a diferença, é uma espécie de ajuste a situações ligeiramente distintas. Sempre leve em consideração questões como esta quando estiver projetando sua interface final com o usuário. Pense em quais variáveis devem ser informadas de antemão e quais podem ser adquiridas de forma interativa elegantemente. No nosso exemplo, optou-se por uma entrada prévia de toda a informação necessária, mas exemplos que lidam com grande volume de variáveis, especialmente aqueles que lidam com dados usualmente raros ou difíceis de obter, tendem a adotar uma abordagem mista.
	Agora, vamos ao código. Desejamos instanciar estas variáveis antes do início da consulta. Na verdade, com a Expert SINTA VCL nunca podemos alterar instâncias de variáveis fora de uma consulta. Como proceder então? Realizando as alterações no início da consulta. O evento OnStart de TExpertSystem é útil especialmente nestas ocasiões:

procedure TForm1.ExpertSystemStart(Sender: TObject);
begin
   ExpertPrompt1.UpdateBase;
   ExpertPrompt2.UpdateBase;
   ExpertPrompt3.UpdateBase;
   ExpertPrompt4.UpdateBase;
   ExpertPrompt5.UpdateBase;
   ExpertPrompt6.UpdateBase;
   ExpertPrompt7.UpdateBase;
   ExpertPrompt8.UpdateBase;
   ExpertPrompt9.UpdateBase;
end;

	Os nove TExpertPrompt do formulário correspondem às nove informações necessárias para uma recomendação de vinho. No início da aplicação, estes estão dispostos na janela de forma que podemos marcar as opções à vontade. Isto não significa de forma alguma que estamos alterando as instâncias das respectivas variáveis. Tais alterações só ocorrerão pela chamada do método UpdateBase de cada TExpertPrompt. O momento ideal para isto é o início da consulta, disparado pelo clique no botão "Consultar". Note que não foi necessário lidar com a propriedade WaitingAnswer de TExpertSystem.
	A janela de resultados (frmResults) e os demais eventos funcionam de forma análoga ao exemplo do capítulo 2, o run-time.


Capítulo
4


A Expert SINTA VCL e 
Bancos de Dados


	Um sistema especialista tem como principal tarefa automatizar um processo de tomada de decisões. Dentro deste objetivo, nada mais adequado que possuir um conjunto de dados já cadastrados e deixar para o sistema a tarefa de calcular os resultados utilizando como fonte de informações estes dados. Assim, relatórios para diversos casos podem ser obtidos neste processamento de registros. Vejamos maneiras fáceis de integração de bases de dados a projetos de sistemas especialistas criados com a Expert SINTA VCL, na versão batch do nosso conhecido SECAJU.

SECAJU – Batch Consultation

Para quem trabalha com o shell Expert SINTA, o sistema SECAJU é familiar. O SECAJU é um sistema simples de diagnóstico de pragas e doenças do cajueiro. Através de perguntas dirigidas ao responsável pela plantação, uma série de prováveis pragas e doenças é apresentada ao final de uma consulta. Suponhamos que levantaram-se dados de diversos agricultores pelo interior do Ceará e agora desejamos submeter ao SECAJU estas informações. Seria imensamente tedioso o processo de resposta a cada consulta e anotação/impressão de resultados. Com a Expert SINTA VCL, é possível criar com facilidade um projeto em Delphi ou C++ Builder que utilize estas informações e gere um relatório com apenas poucas linhas de programação.

Na linha de montagem

	Os principais objetos utilizados neste projeto são:

•	ExpertSystem (classe TExpertSystem): sistema especialista com as regras do SECAJU;
•	MemoResults (classe TMemo): é aqui que o "relatório" de resultados será "impresso";
•	DebugPanel (classe TDebugPanel): depurador que irá exibir, a título de ilustração, o comportamento da máquina de inferência. Não tem uma utilidade direta na aplicação, mas é interessante notar como a máquina de inferência atua em consultas sucessivas;
•	btnStart (classe TButton): dispara a seqüência de consultas;

Para esta aplicação, foram criadas duas tabelas: Clientes.db e Casos.db. A tabela de clientes tem a função somente de armazenar o nome dos clientes que consultarão o SECAJU. Em Casos.db, cada registro contém informações sobre o estado da plantação de cada cliente, um registro para cada (relacionado pelo campo cd_cliente – código do cliente).
Os demais campos de Casos.db correspondem a variáveis do domínio do SECAJU que devem ser fornecidas pelo usuário (para saber que variáveis são respondidas por usuário, abra a base de conhecimento no Expert SINTA e utilize a opção Depurar  Dependências...). Assim, o campo Cbcatd corresponde à variável "castanha broqueada com amêndoa totalmente destruída", Gnroi à "galeria nos ramos ou inflorescências", Inflorescencias à "inflorescências" e assim vai até conter todas as variáveis.
Por que esta ordem foi escolhida? Primeiro vejamos como as consultas são disparadas: ao clicarmos o botão btnStart, as seguinte ações ocorrem:

procedure TForm1.btnStartClick(Sender: TObject);
begin
   memoResults.Clear;
   TableCasos.First;
   while not TableCasos.EOF do begin
      ExpertSystem.StartConsultation;
      TableCasos.Next;
   end;
   ShowMessage('Todas as consultas finalizadas!');
end;

Ou seja, a tabela de casos é varrida e uma consulta é iniciada (e finalizada) para cada registro. Os valores correntes são lidos quando necessários, ou seja, através do evento OnPrompt:

procedure TForm1.ExpertSystemPrompt(Sender: TObject; V: Integer);
begin
   ExpertSystem.AttribVarFromBinary(V,IntToStr(GetValueFromTable(V)));
   ExpertSystem.WaitingAnswer := false;
end;

A variável em demanda V receberá suas instâncias de acordo com o que for indicado no registro corrente. Por questões de modularidade, resolvemos colocar o procedimento que mapeia um campo da tabela no valor desejado em separado:

function TForm1.GetValueFromTable(V: integer): integer;

   function VarMap(V: integer): integer;
   begin
      Result := V - 4;
   end;

begin
   Result := TableCasos.Fields[VarMap(V)].AsInteger;
end;
 
	Como funciona? Desejamos saber o(s) valor(es) de V, onde V é o código da variável. A subfunção VarMap indica qual a posição do campo com o(s) valor(es) de V dentro do registro corrente. A complexidade desta função variará de acordo com seu sistema especialista e o modo como a tabela foi organizada. No caso do SECAJU Batch Consultation, a variável do campo número 1, "castanha broqueada...", possui código 5 (o primeiro campo é o campo número 0: "código do cliente"). A variável do campo 2, código 6; do campo 3, código 7. É claro que isto foi feito porque sabíamos dos códigos de cada variável (veja o capítulo I para saber como extrair estes códigos). O SECAJU também favorece um mapeamento fácil, porque todas as variáveis necessárias em uma pergunta correspondem às variáveis de código no intervalo 05 a 36. Se, por exemplo, a variável 25 não fosse uma variável de pergunta, mas calculada pelo sistema, obviamente não apareceria na tabela. Assim, a função VarMap precisaria ser reformulada:

function VarMap(V: integer): integer;
begin
   if V < 25 then
      Result := V – 4
   else
      Result := V – 5; //saltando a variável #25
end;

	Outras combinações podem ser necessárias dependendo do seu sistema.
	Quanto aos valores dos campos, também fica a cargo do programador a escolha da codificação que será feita. A mais direta é utilizar o código do valor em cada campo. Ou seja, se, para dado caso, a variável "inflorescências" tem como valor "murchas ou secas" (código 29 no arquivo BCM), o campo Inflorescencias teria valor 29. Mas isto traria problemas quanto a variáveis multivaloradas. No nosso exemplo, utilizamos uma combinação binária. Por exemplo, suponhamos que temos uma variável que pode assumir quatro valores. Sua combinação binária é um somatório baseado em potências de dois: se escolhemos o primeiro e o quarto valor, a combinação será 2^(1-1)^ + 2^(4-1)^ = 9. Se escolheu-se o segundo, terceiro e o quarto, a combinação será 2^(2-1)^ + 2^(3-1)^ + 2^(4-1)^ = 14. Assim podemos indicar um conjunto de valores para uma variável através de um único campo. O método AttribVarFromBinary, de TExpertSystem, faz o resto do trabalho (consulte a referência ao final deste manual caso precise de maiores informações sobre este método). Você pode saber a posição de cada valor para uma variável através da exportação de códigos (utilize o shell para isso) ou simplesmente abrindo a janela de variáveis no shell Expert SINTA: os valores de cada variável aparecerão ordenados. Consulte a documentação do shell para maiores informações.
	MAS ATENÇÃO: uma desvantagem clara desta abordagem de codificação é a dependência da ordem dos valores. Se você precisar mudar a ordem e estiver utilizando este método, não esqueça de modificar as tabelas. Para os valores DESCONHECIDO, SIM e NÃO, o método AttribVarFromBinary utiliza as constantes –1, –2 e –3, respectivamente. Para saber mais sobre as constantes utilizadas na Expert SINTA VCL, examine o arquivo ExConsts.pas. Outra desvantagem é a impossibilidade do uso de graus de confiança nesta entrada codificada.
	Finalmente, no evento OnPrompt, não esquecer de ExpertSystem.WaitingAnswer := false após a resposta, indicando à máquina de inferência que a mesma já foi obtida.


Além da 
Expert SINTA VCL

A orientação a objetos do Object Pascal permite o acréscimo elegante de novas funcionalidades à Expert SINTA VCL.

Capítulo 5 – Estendendo a Expert SINTA VCL
Os passos essenciais para criação de novos componentes que se integrem à biblioteca de forma transparente e possam ser distribuídos para outros desenvolvedores. Apresentando um novo componente: TListExpertPrompt!

Capítulo 6 – Interfaces: comunicação entre os objetos
Uma característica interessante é a resposta automática de certos componentes em relação a mudanças no sistema especialista associado. Como implementar estas atualizações automáticas em componentes criados por você?


Capítulo
5


Estendendo a Expert SINTA VCL


Para aqueles que não estão satisfeitos com as funcionalidades fornecidas pelos componentes da Expert SINTA VCL, é possível, de maneira fácil, a criação de novos componentes utilizando-se o conjunto de estruturas de dados implementado. Com o uso de "objetos de interface", objetos que respondem automaticamente a eventos disparados pelo sistema especialista envolvido podem ser construídos, facilitando seu uso por terceiros.

O básico dos objetos Expert SINTA

Uma melhor compreensão do funcionamento interno dos componentes padrões da biblioteca é bastante útil. Estude a implementação descrita nos arquivos fonte (*.PAS) pelos quais a biblioteca foi instalada. Para tal, procure não se preocupar com a classe TExpertSystem, mas com os controles de interface gráfica implementados no arquivo ExCtrls.Pas. De TExpertSystem, deve-se compreender bem sua interface de métodos e propriedades (o mesmo vale para as estruturas de dados encontradas em ExDataSt.Pas). Além disso, é importante algum conhecimento da estrutura de dados utilizada na representação de conhecimento. Informações detalhadas sobre TExpertSystem e sobre estruturas de dados podem ser encontradas no manual de referência, último capítulo do presente documento. Outro requisito indispensável é o entendimento de programação de componentes em Object Pascal.
Neste capítulo, vamos criar ExCtrls2.Pas, um arquivo contendo um novo componente de interface gráfica. Mas, antes de prosseguirmos, vale ressaltar: de forma alguma é essencial a criação de novos componentes para a implementação de aplicações mais flexíveis. Muito pode ser feito simplesmente com chamadas aos métodos das classes básicas da Expert SINTA VCL e o bom uso de seus eventos.

Um substituto para TExpertPrompt

Cansado daquele mesmo TExpertPrompt de sempre? Que tal implementarmos um novo objeto de entrada de dados, talvez mais adequados a suas necessidades?
	Nesta seção vamos estender um componente TListBox (consulte a documentação da Visual Componente Library da Borland para maiores informações). Esta list box permitirá que marquemos opções de resposta para uma dada variável, inclusive com opção de marcação de vários itens (para variáveis multivaloradas). Com fins de simplicidade, não serão incluídas opções para entrada de graus de confiança para as respostas. Vamos batizar este componente de TListExpertPrompt.
	Observação: para melhor entendimento deste capítulo, talvez seja mais útil que o leitor primeiramente instale este componente e compreenda sua utilidade.
 
Métodos e propriedades

Qual serão os parâmetros para nosso novo componente? Assim como em TExpertPrompt, os parâmetros básicos são o sistema especialista e a variável cujos valores de entrada serão disponibilizados. Como é padrão no Object Pascal, criam-se funções que manipularão estas propriedades. 
As propriedades adicionadas a TCustomListBox serão:

•	ExpertSystem (classe TExpertSystem): o sistema especialista ligado a este componente. O método de manipulação, seguindo a nomenclatura tradicional do Object Pascal, será SetExpertSystem. A variável interna será FExpertSystem. Para maiores informações sobre propriedades e convenções de nomenclatura, consulte a documentação do Delphi;
	
•	VarCode (tipo integer): o código da variável cujos valores de entrada serão disponibilizados para escolha;

Um método necessário é aquele que lê os valores marcados e atualiza a base de fatos do sistema. Vamos chamá-lo (assim como em TExpertPrompt) de método UpdateBase. Os métodos adicionais de TListBox se resumem em:

•	procedure UpdateBase: a partir dos valores marcados na lista, atualiza-se a base de fatos do sistema especialista respectivo;

Uma primeira declaração deste nosso novo componente seria:

TListExpertPrompt = class(TCustomListBox)
   private
      FVarCode: integer;
      FExpertSystem: TExpertSystem;
	FVarBinary: boolean; //A ser explicada posteriormente
   protected
      procedure SetExpertSystem(ES: TExpertSystem);
      procedure SetVarCode(vc: integer);
   public
      function UpdateBase: boolean;
   published
      property ExpertSystem: TExpertSystem 
         read FExpertSystem write SetExpertSystem;
      property VarCode: integer read FVarCode write SetVarCode;
end;

	Vamos ver como a propriedade VarCode é aproveitada em TListExpertPrompt:

procedure TListExpertPrompt.SetVarCode(vc: integer);
begin
   FVarCode := vc;
   if (FExpertSystem <> nil) and (vc <> 0) and (Parent <> nil) and
      (not FExpertSystem.EmptyBase) then
   begin
      FExpertSystem.Vars.Seek(vc);
      if FExpertSystem.Vars.Blind then begin
         VarCode := 0;
         Exit;
      end;
      if FExpertSystem.Vars.Numeric then begin
         VarCode := 0;
         raise EExpertSystem.Create('Este prompt não exibe valores' 
                                    + ' de variáveis numéricas!');
      end;
      MultiSelect := FExpertSystem.Vars.Multi;
      FExpertSystem.ValuesList(vc, TStringList(Items));
      {Variável do tipo Sim/Não}
      FVarBinary := (Items.Objects[0] = nil); 
      FVarCode := vc;
   end
   else
      if vc = 0 then Clear;
end;

	Primeiramente, FVarCode recebe o valor passado pela propriedade (vc). A primeira condição verifica se existe um componente ExpertSystem associado e se este já apresenta uma base de conhecimento carregada. Esta verificação é bastante comum nos componentes da Expert SINTA VCL (veja o arquivo ExCtrls.Pas).  As duas próximas condições verificam se a variável realmente existe e se ela não é numérica (este componente não é feito para entrada de variáveis numéricas).
	Finalmente, a propriedade MultiSelect da list box é ajustada de forma a permitir a seleção de mais de um item caso a variável seja multivalorada. A parte mais difícil já está implementada em TExpertSystem: através do método ValuesList, pode-se preencher uma lista TStringList com os valores, ordenados por suas posições, de uma variável. Consulte mais informações sobre TExpertSystem.ValuesList na referência encontrada ao final deste documento. ValuesList preenche a lista de retorno (Items) tanto com as strings dos valores como com ponteiros para as estruturas de dados que armazenam estes valores na memória. Para variáveis binárias, as strings retornadas são "Sim" e "Não", mas nenhum ponteiro é retornado (Items.Objects[i] = nil, para todo i dentro da faixa de valores).
	FVarBinary é um valor interno utilizado para saber posteriormente se esta variável é binária ou não.
	O método UpdateBase é implementado do seguinte modo:

function TListExpertPrompt.UpdateBase: boolean;
var
   i, total: integer;
   bin_value: integer;
begin
   if (FVarCode = 0) or (FExpertSystem = nil) or 
      FExpertSystem.BrokenSequence or
      (not FExpertSystem.ExecutionMode) or
      ((not FExpertSystem.WaitingAnswer) and 
      (FExpertSystem.Wait or FExpertSystem.Trace))
      then
      Result := false
   else begin
      if FVarBinary then begin
         if Selected[0] then
            FExpertSystem.AttribVarFromBinary(FVarCode, YES)
         else
            FExpertSystem.AttribVarFromBinary(FVarCode, NO);
      end
      else begin
         total := Items.Count - 1;
         bin_value := 0;
         for i := 0 to total do
             if Selected[i] then bin_value := bin_value + 
                                              trunc(exp(ln(2)*i));
         if bin_value = 0 then bin_value := UNKNOWN;
         FExpertSystem.AttribVarFromBinary(FVarCode, bin_value);
      end;
      Result := true
   end;
end;

	A condição inicial verifica se: existe uma variável selecionada em VarCode; o sistema especialista está em consulta; no momento o usuário não está voltando para a pergunta anterior (propriedade BrokenSequence) e se não está em pausa, esperando uma resposta (WaitingAnswer e outros).
	Para variáveis binárias, a ação é bem simples: se o primeiro item é o selecionado, a resposta SIM é acrescentada na base de fatos pelo método AttribVarFromBinary. Se o segundo item é o selecionado, a resposta é NÃO.
	Para as demais variáveis, criamos o número equivalente ao somatório das potências de dois respectivas aos itens selecionados, conforme exige o método AttribVarFromBinary. Desta forma, com um só comando atualizamos a base de fatos. Para maiores informações sobre AttribVarFromBinary, consulte a referência ao final deste documento.
	E quanto ao método SetExpertSystem? Basicamente, poderíamos ter definido a propriedade ExpertSystem como:

property ExpertSystem: TExpertSystem read FExpertSystem 
                                     write FExpertSystem;

	Porém, assim como em vários componentes da Expert SINTA VCL, seria interessante realizar alterações automáticas em TListExpertPrompt quando definimos um sistema especialista. Por exemplo, em TExpertPrompt, quando dizemos qual o sistema especialista relacionado a um objeto desta classe, onde o valor de VarCode já foi definido, o componente automaticamente exibe sua lista de valores. Quando o sistema especialista é destruído ou uma nova base de conhecimento é carregada, as alterações se refletem imediatamente em TExpertPrompt. O próximo capítulo irá lidar com a questão de troca de mensagens entre componentes de forma a permitir ao usuário de seus componentes o uso dos mesmos sem preocupações de garantir a integridade relacional.


Capítulo
6


Interfaces: 
comunicação entre os objetos


Quando vemos um objeto como TDebugPanel em ação, notamos claramente que existe uma espécie de troca de mensagens entre seu objeto ExpertSystem relacionado e o mesmo. A máquina de inferência, ao avançar, indica ao depurador sua nova posição. O depurador, ao receber um valor para ExpertSystem, efetua uma espécie de "cadastro" no sistema especialista de forma a poder ser "reencontrado" pelo sistema quando atualizações se fazem necessárias.
A comunicação entre objetos da Expert SINTA VCL se faz necessária por dois motivos: para sabermos atualizar componentes relacionados quando necessário e para indicar a estes componentes quando outro objeto de seu interesse foi destruído. Para tal, utilizam-se o que este manual convencionou de objetos de interface da Expert SINTA VCL.
Para uma boa compreensão desta seção, recomenda-se razoável conhecimento em polimorfismo no contexto de orientação a objeto.

O que são interfaces?

Em primeiro lugar, não confunda o conceito de interface utilizado aqui com o conceito utilizado pela tecnologia COM. No Delphi 3.0, temos objetos de interface, mas estes não estão relacionados de forma alguma com a Expert SINTA VCL.
	Para que um objeto A invoque métodos do objeto B e vice-versa, é necessário que A faça referência a B, bem como o oposto. Isto ocorre diversas vezes na Expert SINTA VCL: um objeto TExpertSystem possui uma lista apontando para diversos controles a ele relacionados, como por exemplo, o objeto TRuleView descrito no primeiro capítulo deste documento. Quando carregamos uma nova base de conhecimento no objeto de sistema especialista, este percorre sua lista de controles relacionados e chama métodos de atualização. Ou seja, de alguma forma, um objeto TRuleView aponta para um dado objeto TExpertSystem que, por sua vez, aponta para o TRuleView original.
	Como implementar este relacionamento? Observe que o comportamento de atualização poderia ser uma característica herdada de um objeto genérico. Por exemplo, ao invés de uma lista de ponteiros TRuleView, a classe TExpertSystem teria uma lista de, digamos, TExpertControl. Esta classe teria um método abstrato, digamos, RefreshLink. Por sua vez, TRuleView seria uma classe derivada de TExpertControl, para implementar esta interface em comum, e de TListBox, de onde herdaria as funcionalidades gráficas. E outra possibilidade se abre: TExpertSystem poderia aceitar qualquer objeto derivado de TExpertControl, como um objeto implementado por terceiros, não somente classes previamente estabelecidas. Estes novos objetos precisariam apenas chamar um método de TExpertSystem que os "registrassem" junto ao sistema especialista, incluindo-os em sua lista de controles relacionados.
	Mas, existe um grande problema. O Object Pascal, linguagem utilizada no Delphi, não apresenta o recurso de herança múltipla! Como fazer então para evitar este problema?
	Uma alternativa é fazer de TExpertControl um objeto de comunicação intermediária entre TExpertSystem e um controle real. Um componente da Expert SINTA VCL, TRuleView, digamos, apontaria para um objeto TExpertControl e este apontaria de volta para a lista de regras. O objeto TExpertSystem relacionado apontaria para TExpertControl, conforme a figura 3:


Figura 3 - Comunicação por "interfaces"
 
	Esta abordagem, utilizada pela Expert SINTA VCL, contorna o problema da falta de herança múltipla à medida do possível. Note que a nomenclatura foi mudada de TExpertControl para TExCtrlInterface para enfatizar que este é apenas um objeto de interface, não o controle em si.

Devo sempre implementar uma nova interface?

As interfaces são utilizadas por componentes que respondem automaticamente a dadas alterações do sistema especialista relacionado, o que é uma decisão do desenvolvedor destes componentes. Como vimos no primeiro capítulo, nem todos os componentes da Expert SINTA VCL são assim e nem sempre vamos desejar implementar um componente que possua uma interface relacionada. Por exemplo, TConsultTree e TAllVars são duas classes que não possuem uma interface respectiva.
	A regra geral é: se você deseja que seu componente seja alertado de determinados eventos do sistema especialista e implementar funções de atualização, então você também necessitará implementar uma interface, o que na realidade é bem simples. Caso contrário, não há necessidade em sequer prosseguir a leitura deste capítulo.
 
Como criar interfaces

A declaração de TExCtrlInterface é:

TExCtrlInterface = class
   protected
      OwnerControl: TObject;
   public
      Kind: byte;
      constructor Create(AKind: byte; AOwnerControl: TObject);
      procedure   Clear; virtual; abstract;
      procedure   RefreshLink(Sender:TExpertSystem);virtual;abstract;
      procedure   DestroyLink; virtual; abstract;
   end;

	Para compreender esta definição, temos que compreender a funcionalidade de um objeto de interface.
	Em primeiro lugar, como descrito em tópicos anteriores deste capítulo, o objeto de interface deve apontar para o objeto "real" que se deseja integrar à Expert SINTA VCL. Este objeto é, na declaração de TExCtrlInterface, o ponteiro OwnerControl. O relacionamento é feito no construtor da interface.
	E é no construtor que também se inicializa um parâmetro essencial: Kind. No módulo ExConsts.Pas, temos as seguintes constantes:

{Interfaces}
   {Para objetos que exibem instâncias de variáveis}
   I_INSTANCE_VIEW = 0;
   {Para objetos que exibem dados relativos a variáveis, como uma  
    pergunta ou os seus possíveis valores}
   I_VARIABLE_VIEW = 1;
   {Para objetos que exibem regras ou trechos de regras}
   I_KB_VIEW = 2;
   {Para objetos que refletem o status da máquina de inferência, como
    um navegador}
   I_STATUS_VIEW = 3;

	De acordo com o valor de Kind, TExpertSystem realizará mudanças nos objetos apontados nos eventos necessários. Os diferente efeitos para diferentes valores de Kind são:

•	I_INSTANCE_VIEW: os objetos são atualizados sempre que novas instâncias de variáveis surgem ou são desfeitas. Isto ocorre basicamente quando uma regra é aceita (e suas conclusões – parte ENTÃO – efetuadas) ou quando o usuário volta para uma pergunta anterior (o que desfaz algumas instâncias). Classes da Expert SINTA VCL correspondentes a esta descrição são TWatchPanel e TValuesGrid;
	
•	I_VARIABLE_VIEW: note bem a diferença – interfaces com Kind igual a I_INSTANCE_VIEW respondem a eventos que lidam com instâncias de variáveis. Estas correspondem a objetos que lidam com propriedades de variáveis. Por "propriedades" de uma variável se entende: lista de valores possíveis e perguntas relacionadas. Classes da Expert SINTA VCL correspondentes a esta descrição são TExpertPrompt e TLabelQuestion;
	
•	I_KB_VIEW:  manipulam objetos que lidam com regras ou trechos de regras. Por exemplo, nossos conhecidos TRuleView e TDebugPanel;
	
•	I_STATUS_VIEW: a máquina de inferência de um sistema especialista pode estar em vários estados: desativada, em consulta, em pausa, voltando para a pergunta anterior. Se seu componente precisa responder a estes eventos, utilize este tipo. O exemplo típico é TExNavigator;

	O método abstrato RefreshLink deve ser implementado por uma classe de interface derivada de TExCtrlInterface, como veremos na continuação de nosso exemplo, o TListExpertPrompt. De um modo geral, a função de cada método é:

•	Clear: invocado quando não há informações a serem exibidas. Em um TRuleView, isto eqüivaleria a limpar a list box. Em um TExpertPrompt, a esconder todas as opções. Em um TLabelQuestion, a limpar o caption do label. Ou seja, assim como os demais métodos da interface, a ação é totalmente dependente do objeto "real" apontado pela interface;
	
•	RefreshLink: atualiza as informações a pedido do sistema especialista relacionado;
	
•	DestroyLink: chamado pelo destrutor do sistema especialista para avisar que o objeto TExpertSystem relacionado não existe mais;

Exemplo prático

Para a real compreensão de como se deve criar e utilizar uma interface para um componente em desenvolvimento, continuemos com o exemplo do capítulo 5, o TListExpertPrompt.
	Vamos definir a interface TListExpertPromptInterface: 

TListExpertPromptInterface = class(TExCtrlInterface)
public
   procedure Clear; override;
   procedure RefreshLink(Sender: TExpertSystem); override;
   procedure DestroyLink; override;
end;

	Como os métodos da classe base TExCtrlInterface são abstratos, devemos implementá-los na interface derivada:

•	O método Clear deve limpar a visualização de dados:

procedure TListExpertPromptInterface.Clear;
begin
   TListExpertPrompt(OwnerControl).VarCode := 0;
end;

•	O  método RefreshLink deve atualizar os dados. No caso de TListExpertPromptInterface, isto é  feito preenchendo-se os itens da lista (TListExpertPrompt(OwnerControl).Items) com os valores da dada variável (VarCode). O método TExpertSystem.ValuesList (ver referência ao final deste documento) faz esta operação automaticamente.

procedure TListExpertPromptInterface.RefreshLink(Sender:TExpertSystem)
begin
   TListExpertPrompt(OwnerControl).ExpertSystem.
      ValuesList(TListExpertPrompt(OwnerControl).VarCode,                                                           
                 TStringList(TListExpertPrompt(OwnerControl).Items));
end;

•	O método DestroyLink é basicamente o mesmo para todos os tipos de interface, indicando que o sistema especialista não é mais válido:

procedure TListExpertPromptInterface.DestroyLink;
begin
   TListExpertPrompt(OwnerControl).ExpertSystem := nil;
end;

	Observe que o objeto OwnerControl é um ponteiro TObject. Assim, o cast explícito é necessário.
	A segunda parte consiste na integração da interface com o componente. Em TListExpertPrompt, a primeira medida ser tomada é quanto à criação da interface.

constructor TListExpertPrompt.Create(AOwner: TComponent);
begin
   inherited Create(AOwner);
   ExpertPromptInterface := 
         TListExpertPromptInterface.Create(I_VARIABLE_VIEW, Self);
end;

	O próximo ponto importante é a associação ao sistema especialista respectivo. É aqui que entra o método SetExpertSystem, associado à propriedade ExpertSystem, conforme foi definido no capítulo anterior.

procedure TListExpertPrompt.SetExpertSystem(ES: TExpertSystem);
begin
   if (FExpertSystem <> ES) and (FExpertSystem <> nil) then
       FExpertSystem.RemoveControl(ExpertPromptInterface);
   FExpertSystem := ES;
   if ES <> nil then begin
      ES.AddControl(ExpertPromptInterface);
      if FVarCode <> 0 then SetVarCode(FVarCode);
   end
   else
      VarCode := 0;
end;

	Observe que a primeira preocupação é verificar se este objeto TListExpertPrompt está associado a algum outro sistema especialista (FExpertSystem <> nil). Se verdadeiro, o método RemoveControl de TExpertSystem encarregar-se-á de "desalocar" a interface respectiva da lista de controles relacionados no sistema especialista.
	Após esta verificação, a interface é associada ao sistema especialista pelo método AddControl de TExpertSystem. A atualização da exibição de dados é feita logo em seguida, o que em TListExpertPrompt corresponde ao método SetVarCode. Claro, tudo isto é feito levando-se em conta que o sistema especialista existe. Se o valor passado for nulo (ES = nil), isto deve eqüivaler a limpar a exibição de informações. Nenhum sistema especialista estará associado à interface implementada em TListExpertPrompt.
	Finalmente, deve-se saber desassociar a interface do sistema especialista e destruí-la quando no destrutor do componente:

destructor TListExpertPrompt.Destroy;
begin
   if FExpertSystem <> nil then
      FExpertSystem.RemoveControl(ExpertPromptInterface);
   ExpertPromptInterface.Free;
   inherited Destroy;
end;

Últimas observações

Quase todas as interfaces derivam de TExCtrlInterface e implementam os métodos abstratos nela definidos. Há uma exceção: a interface de depuradores, como TDebugPanel.
	TExDebugInterface é definida em ExSystem.Pas como:

TExDebugInterface = class(TExCtrlInterface)
protected
    function  GetActiveTrace: boolean; virtual; abstract;
    procedure SetActiveTrace(ac: boolean); virtual; abstract;
public
    property  ActiveTrace: boolean read GetActiveTrace 
                                   write SetActiveTrace;
    procedure Activate; virtual; abstract;
    procedure Update(rule: integer; order: integer; 
              is_tail: boolean); virtual; abstract;
end;

	Além dos métodos e propriedades de TExCtrlInterface, esta interface acrescenta as seguintes funcionalidades:

•	ActiveTrace: indica que a máquina de inferência deve esperar por um passo do depurador para poder continuar;
	
•	Activate: quando invocado, paralisa a máquina de inferência se ActiveTrace é verdadeira ou de acordo com indicações do sistema especialista;
	
•	Update(rule, order, is_tail): informa ao depurador que máquina de inferência está na regra rule, na order-ésima premissa (ou conclusão) da mesma, sabendo-se que é uma premissa se is_tail é verdadeira e uma conclusão se is_tail é falsa ;

Ao associar um novo depurador, deve-se, além de associá-lo ao sistema especialista pelo método AddControl, indicar que este é o depurador do sistema:

ES.DebugControl := TMyExDebugInterface(MyExDebugInterface);

	Ou seja, a implementação atual de TExpertSystem só aceita um depurador por vez. Para maiores detalhes, veja a implementação de TDebugPanel.SetExpertSystem em ExCtrls.Pas.


Manual de Referência

Este seção descreve a funcionalidade das principais classes que formam a Expert SINTA Visual Component Library de forma a servir de referência. Para uma compreensão melhor do relacionamento entre estas classes, consulte as seções anteriores deste documento. 

Capítulo 7 – Estrutura de Dados
Relação das classes que formam a estrutura de dados utilizada na representação do conhecimento.

Capítulo 8 – Os Componentes Visuais
Os métodos e as propriedades dos onze componentes da Expert SINTA VCL.


Capítulo
7


Estrutura de Dados


Nesta seção, descrevemos as classes que formam a estrutura de dados representando a base de conhecimento. Embora não seja absolutamente necessária a compreensão das mesmas, estas podem ser úteis na criação de componentes personalizados para comunicação com o sistema especialista. Também é um exemplo funcional de implementação de regras de produção.
Cada definição de classe pode ser encontrada pelo seguinte cabeçalho, onde cada linha possui seu significado:


TMinhaClasse
Unidade: Exemplo
TObject  TObjetoDerivado  ...  TMinhaClasse


	Antes de compreender uma classe, tenha certeza de que compreende suas classes de base. As classes de base não citadas aqui pertencem à Visual Component Library da Borland. Consulte a documentação do Borland Delphi ou C++ Builder se precisar.

Visão Geral


	Para melhor compreender a estrutura que representa as regras de produção, vejamos o seguinte exemplo (extraído da base SECAJU):


	SE		galerias nos ramos ou inflorescências = Sim
	OU		inflorescências = murchas ou secas
	E		brotações novas murchas = Sim
	E		orifícios laterais nos ramos ou inflorescências = Sim
	ENTÃO	praga = broca das pontas CNF 90%


Figura 4 - Exemplo de regra de produção

	Chamemos as conclusões de uma regra de cabeças da regra e os antecedentes de  caudas. Esta notação é proveniente da linguagem PROLOG, na qual as conclusões encabeçam as cláusulas.

	Para o projetista do conhecimento que cria bases utilizando o Expert SINTA, o seguinte critério para definições de assertivas deve ser seguido:

( I ). A estrutura de cada cauda (premissa) deve obedecer ao seguinte modelo: 

<atributo>	<operador>	<valor>	<conectivo>	
			
onde:

•	atributo é uma variável capaz de assumir uma ou múltiplas instanciações no decorrer da consulta à base de conhecimentos. Cabe ao desenvolvedor definir o tipo de atributo. Um atributo é uma entidade capaz de armazenar listas de valores cujo significado depende do contexto da base. Por exemplo, um atributo doença, no SECAJU, é capaz de ser instanciado a qualquer um ou vários elementos da lista de valores pré-definida [mofo preto, oidio, antracnose]. Números também podem ser atribuídos a variáveis;

•	operador é um elo de ligação entre o atributo e o valor da premissa que define o tipo de comparação a ser realizada. São operadores relacionais: =, >, <=, <>, entre outros;

•	valor é um item de uma lista a qual foi previamente criada e relacionada a um atributo. Como sabemos, a interface visual do Expert SINTA foi idealizada de modo a minimizar o trabalho do responsável pela implementação da base de conhecimentos. Para tanto, o Expert SINTA exige que os atributos sejam definidos antes de criarmos uma regra que o utilize. No momento da criação de um atributo, também é possível que seja definida uma lista de valores que constituirá o universo ao qual suas instanciações devem necessariamente pertencer. As listas criadas podem ser utilizadas para construção de menus, caso o sistema especialista necessite efetuar uma pergunta ao usuário referente àquele determinado atributo. Um valor também pode ser um número, caso o atributo o permita;

•	conectivo é um dos seguintes elementos utilizados na lógica clássica: E, OU. Sua função é unir a sentença ao conjunto de premissas que formam a seção de antecedentes de uma regra.

Esta estrutura é representada através de uma árvore binária da classe TTails (descrita a seguir).

( II ). A estrutura de cada cabeça (conclusão) deve obedecer ao seguinte modelo:

<atributo>	=	<valor>	<grau de confiança>	

onde:

•	atributo equivale ao mesmo atributo usado em caudas;

•	"=" é um operador de atribuição e não de igualdade. Ou seja, o atributo, nas cabeças de regra, é sempre instanciado a um valor. Dependendo se a variável pode ou não acumular múltiplas instanciações, o novo valor substituíra o antigo ou será empilhado com os demais.

•	valor equivale ao mesmo valor utilizado em caudas;

•	grau de confiança é uma porcentagem indicando a confiabilidade daquela conclusão específica da regra. O grau de confiança varia de 0% a 100%.

	O seguinte diagrama exibe todas as árvores binárias que formam a estrutura de dados de uma base de conhecimento. Observe que a mesma pode ser apresentada com um esquema relacional:


Figura 5 - Principais entidades que formam a estrutura de dados
	Ao programar a nível de estrutura de dados, procure pensar como se estivesse utilizando tabelas de um banco de dados relacional. Extraímos informações de cada "tabela" (árvore binária) de um "registro" corrente (aqui denominado de nó) com atributos (propriedades). Para conseguir informações, por exemplo, de uma variável, deve-se navegar pela "tabela" Vars de um objeto TExpertSystem usando comandos como Seek (para ir direto ao ponto), MoveFirst, MoveLast, MoveNext, MovePrevious. A função Blind indica se existe algum "registro" corrente (funcionaria mais ou menos como um "EOF" - end of file).
	Por exemplo, usando a regra mostrada anteriormente, podemos organizar a estrutura de dados do seguinte modo (observe que os códigos são gerados durante a construção da base pelo shell Expert SINTA):

Vars
Código	Nome	Multivalorada?	Numérica?	
1	galerias nos ramos ou inflorescências	Não	Não	
2	inflorescências	Sim	Não	
3	brotações novas murchas	Não	Não	
4	orifícios laterais nos ramos ou inflorescências	Não	Não	
5	praga	Sim	Não	

Values
Código	Código da variável	Nome	Posição	
1	2	murchas ou secas	1	
2	2	existem lesões necróticas	2	
3	5	broca das pontas	1	
4	5	besourinho vermelho	2	
(existem lesões necróticas e besourinho vermelho foram colocados apenas para ilustrar o comportamento de mais de um valor para uma mesma variável)

Tails
Código da regra	Código da variável	Operador	Código do valor	Conectivo	Negação	
1	1	=	-2	OU	Não	
1	2	=	1	E	Não	
1	3	=	-2	E	Não	
1	4	=	-2	ENTÃO	Não	
O valor -2 é especial e corresponde ao SIM lógico. Para maiores detalhes, ver a descrição da classe TTails.

Heads
Código da regra	Código da variável	Código do valor	CNF	
1	5	3	0.9	

As Classes

	A seguir, a descrição das "tabelas" mais importantes e demais classes de estrutura de dados.

TCodeNameIndexTree
Unidade: ExDataSt
TObject  TSingleIndexTree  TCodeNameIndexTree

Descrição
	
Classe de base para a estrutura de dados. Não é usada diretamente por TExpertSystem. É uma árvore binária de dois índices, onde seus nós armazenam um código mais uma determinada string (um nome). Os nós são indexados tanto por código como por nome.

Propriedades

Propriedade	Visibilidade	Default	Documentada em	
Blind	public		TSingleIndexTree	
Code	public		TSingleIndexTree	
CurrentKey	public	PRIMARY_KEY	TCodeNameIndexTree	
Name	public		TCodeNameIndexTree	
RecordCount	public		TSingleIndexTree	

CurrentKey: integer
Índice atual usado pelos métodos Move... e pelas propriedades. Os valores possíveis são: PRIMARY_KEY e BY_NAME;

Name: string
Nome do nó corrente da árvore;

Métodos

Método	Visibilidade	Documentado em	
Delete	public	TSingleIndexTree	
GetFreeCode	public	TSingleIndexTree	
Insert	public	TCodeNameIndexTree	
MoveFirst	public	TSingleIndexTree	
MoveLast	public	TSingleIndexTree	
MoveNext	public	TSingleIndexTree	
MovePrevious	public	TSingleIndexTree	
Seek	public	TCodeNameIndexTree	
SeekByName	public	TCodeNameIndexTree	

procedure Insert(c: integer; name: string); 
Insere um nó novo. Nesta árvore, o nó é um código mais um nome. Os  nós são ordenados por "c" ou "name";

procedure Seek(key: integer); override;
Procura por um nó de código "key". Se bem-sucedido, a propriedade Blind é definida como falso. Caso contrário, como verdadeiro. O índice corrente é mudado para PRIMARY_KEY, índice por código;

procedure SeekByName(name: string); virtual; 
Procura por um nó de nome "name". Se bem-sucedido, a propriedade Blind é definida como falso. Caso contrário, como verdadeiro. O índice corrente é mudado para BY_NAME, índice por nome;


TFormulas
Unidade: ExDataSt
TObject  TSingleIndexTree  TCodeNameIndexTree  TFormulas

Descrição
	
Qualquer associação (atribuição, comparação) de um valor a uma variável numérica é considerada como função do usuário. Essa função é interpretada pelo parser da máquina de inferência. É nessa árvore que as expressões são guardadas.

Propriedades

Propriedade	Visibilidade	Default	Documentada em	
Blind	public		TSingleIndexTree	
Code	public		TFormulas	
CurrentKey	public	PRIMARY_KEY	TCodeNameIndexTree	
Name	public		TFormulas	
RecordCount	public		TSingleIndexTree	

Code: integer
Código arbitrário para relacionar a fórmula a uma cabeça ou cauda de regra;

Name: string
A expressão a ser analisada pelo parser interno;


THeads
Unidade: ExDataSt
TObject  TSingleIndexTree  THeads

Descrição
	
Representa as cabeças (conclusões) de uma regra. De acordo com o que foi descrito no começo desta seção, uma conclusão é formada por três atributos: código da variável + código do valor (ou fórmula) + grau de confiança daquela assertiva. Lembre-se que a operação de atribuição ("=") está implícita.

Propriedades

Propriedade	Visibilidade	Default	Documentada em	
Blind	public		TSingleIndexTree	
Code	public		THeads	
CNF	public		THeads	
RecordCount	public		TSingleIndexTree	
ValCode	public		THeads	
VarCode	public		THeads	

Code: integer
Código da regra associada. A ordem das conclusões é definida pela ordem na qual elas aparecem na árvore. Ao usarmos o método Seek(x), o nó corrente passa a ser a primeira conclusão da regra de código x. Com o método MoveNext, é possível iterar por todas as conclusões;

CNF: single
Grau de confiança (confidence factor) da atribuição. Varia de 0 a 1;

ValCode: integer
Código do valor (ou fórmula matemática) que está sendo atribuído a uma variável;

VarCode: integer
Código da variável que está sendo instanciada por uma atribuição;

Métodos

Método	Visibilidade	Documentado em	
Delete	public	TSingleIndexTree	
GetFreeCode	public	TSingleIndexTree	
Insert	public	THeads	
MoveFirst	public	TsingleIndexTree	
MoveLast	public	TsingleIndexTree	
MoveNext	public	TsingleIndexTree	
MovePrevious	public	TsingleIndexTree	
Seek	public	TsingleIndexTree	

procedure Insert(c: integer; c_var: integer; c_val: integer; cnf: single);
Insere um novo nó, onde "c" é o código da regra relacionada, "c_var" o código da variável em atribuição, "c_val" o código do valor (ou fórmula) atribuído, "cnf" o grau de confiança da conclusão;


THelpContexts
Unidade: ExDataSt
TObject  TSingleIndexTree  THelpContexts

Descrição
	
São informações sobre possíveis contextos de ajuda de um dado valor de uma variável objetivo, de forma a chamar um arquivo de ajuda no tópico desejado, dependendo da variável. O shell Expert SINTA usa esses valores na janela de exibição de resultados.

Propriedades

Propriedade	Visibilidade	Default	Documentada em	
Blind	public		TSingleIndexTree	
Code	public		THelpContexts	
Context	public		THelpContexts	
RecordCount	public		TSingleIndexTree	

Code: integer
Código relacionado a um dado valor;

Context: integer
Número do contexto de ajuda dentro do arquivo de ajuda;

Métodos

Método	Visibilidade	Documentado em	
Delete	public	TSingleIndexTree	
GetFreeCode	public	TSingleIndexTree	
Insert	public	THelpContexts	
MoveFirst	public	TSingleIndexTree	
MoveLast	public	TSingleIndexTree	
MoveNext	public	TSingleIndexTree	
MovePrevious	public	TSingleIndexTree	
Seek	public	TSingleIndexTree	

procedure Insert(c: integer; con: integer);
Insere um novo nó, onde "c" é o código do valor relacionado, "con" é o número do contexto de ajuda;


TInfos
Unidade: ExSystem
Este tipo de dado é um registro (record), não uma classe

Descrição
	
Informações gerais sobre a base de conhecimento, como o seu nome, nome dos autores, texto de abertura, entre outras.

Propriedades

Propriedade	Visibilidade	Default	Documentada em	
Authors			TInfos	
HelpFile			TInfos	
Name			TInfos	
Overture			TInfos	
ShowIntro			TInfos	

Authors: string
O(s) nome(s) do(s) autor(es);

HelpFile: string
Arquivo de ajuda relacionado, utilizado principalmente para exibir informações sobre resultados atingidos;

Name: string
Nome do sistema especialista criado;

Overture: TStringList
Texto explicativo que pode ser utilizado no início de uma consulta;

ShowIntro: boolean
Informa se uma tela de abertura com o texto contido em Overture deve ser exibida. O shell exibe esta tela automaticamente, mas, caso se esteja criando um projeto com a Expert SINTA VCL, cabe ao implementador criá-la ou não;


TQuestions
Unidade: ExDataSt
TObject  TSingleIndexTree  TQuestions

Descrição
	
São as strings utilizadas, por exemplo, em TLabelQuestion para exibir o texto de uma pergunta definida pelo criador da base. Essas strings são usadas para deixar mais claro ao usuário qual a intenção de uma dada pergunta. São mais úteis sob o Expert SINTA, que não possui um editor de janelas. Em um ambiente como o Borland Delphi, podem ser substiuídas por labels comuns. Porém, com o uso de perguntas definidas na própria base de conhecimento, o trabalho pode ser mais automatizado, como é mostrado no Expert SINTA RunTime (código fonte - para Borland Delphi - incluso no pacote da Expert SINTA VCL).

Propriedades

Propriedade	Visibilidade	Default	Documentada em	
Blind	public		TSingleIndexTree	
Code	public		TQuestions	
Question	public		TQuestions	
RecordCount	public		TSingleIndexTree	
UseCNF	public		TQuestions	
Why	public		TQuestions	

Code: integer
Código da pergunta, relacionado ao código de uma dada variável;

Question: string
A pergunta propriamente dita;

UseCNF: boolean
Propriedade usada pelo desenvolvedor para identificar se tal pergunta fica boa ou não com entradas que permitem fatores de confiança. Por exemplo, em perguntas do tipo "Existem chances de chuva amanhã?" são adequadas com o uso de CNFs, enquanto "Qual o seu sexo?" não. Tal propriedade é usada pela classe TExpertPrompt e por qualquer um que queira construir controles de entrada de valores para uma base de conhecimento em execução;

Why: TStringList
Um pequeno texto de uso facultativo que explica qual a importância daquela pergunta. Leve em consideração que definir um valor para esta propriedade implicar em copiar strings para um TStringList interno, e não usar o valor atribuído como ponteiro;

Métodos

Método	Visibilidade	Documentado em	
Delete	public	TSingleIndexTree	
GetFreeCode	public	TSingleIndexTree	
Insert	public	TQuestions	
MoveFirst	public	TSingleIndexTree	
MoveLast	public	TSingleIndexTree	
MoveNext	public	TSingleIndexTree	
MovePrevious	public	TSingleIndexTree	
Seek	public	TSingleIndexTree	

procedure Insert(c: integer; q: string; w: TStringList; uc: boolean);
Insere um novo nó, onde "c" é o código da variável relacionada, "q" é o texto da pergunta em si, "w" é uma lista de strings com a explicação respectiva (para nenhuma explicação, passe nil - NULL - como valor), "uc" é o indicativo do uso ou não de grau de confiança;


TRules
Unidade: ExDataSt
TObject  TSingleIndexTree  TCodeNameIndexTree  TValues  TRules

Descrição
	
Reúne informações gerais sobre a regra, a saber: o nome e sua posição em relação às demais. O conteúdo (premissas e conclusões) está em árvores do tipo TTails e THeads. Para maiores detalhes, consulte as classes de base e os comentários no começo deste capítulo.

Propriedades

Propriedade	Visibilidade	Default	Documentada em	
Blind	public		TSingleIndexTree	
Code	public		TSingleIndexTree	
CurrentKey	public	PRIMARY_KEY	TValues	
Name	public		TCodeNameIndexTree	
Position	public		TValues	
RecordCount	public		TSingleIndexTree	
VarCode	public		TValues	

Position: integer
Indica a posição da regra em relação às demais. Não confundir com a propriedade Code, tão somente um código de controle criado pelo Expert SINTA. TRules é um reaproveitamento de TValues, onde Position esconde a propriedade original de TValues. Use o método SeekByVar para procurar por Position;

Métodos

Método	Visibilidade	Documentado em	
Delete	public	TSingleIndexTree	
GetFreeCode	public	TSingleIndexTree	
Insert	public	TRules	
MoveFirst	public	TSingleIndexTree	
MoveLast	public	TSingleIndexTree	
MoveNext	public	TSingleIndexTree	
MovePrevious	public	TSingleIndexTree	
Seek	public	TValues	
SeekByName	public	TValues	
SeekByVar	public	TRules	

procedure Insert(c: integer; name: string; position: integer; dummy: integer); 
Insere um novo nó. Nesta árvore, o nó representa uma regra (código + nome + posição; o último valor não é usado) . Os  nós são ordenados por c, name ou position;

procedure SeekByVar(c_var: integer); 
Encontrar o primeiro nó cuja posição é "c_var". O índice corrente é mudado para BY_VAR, índice por código de variável, interpretada em TRules como código de posição. 


TSingleIndexTree
Unidade: ExDataSt
TObject  TSingleIndexTree

Descrição
	
Classe de base para a estrutura de dados. Não é usada diretamente por TExpertSystem. É uma árvore binária de um único índice, onde seus nós armazenam somente uma informação: um código, sendo este código utilizado para sua indexação.

Propriedades

Propriedade	Visibilidade	Default	Documentada em	
Blind	public		TSingleIndexTree	
Code	public		TSingleIndexTree	
RecordCount	public		TSingleIndexTree	

Blind: boolean
Indica se existe ou não algum nó corrente. Por exemplo, quando procuramos um nó e ele não existe, esta propriedade retorna true. A grosso modo, pode ser comparado com uma propriedade EOF de uma tabela de banco de dados;

Code: integer
Código do nó corrente da árvore;

RecordCount: integer
Retorna o total de nós da árvore;

Métodos

Método	Visibilidade	Documentado em	
Delete	public	TSingleIndexTree	
GetFreeCode	public	TSingleIndexTree	
Insert	public	TSingleIndexTree	
MoveFirst	public	TSingleIndexTree	
MoveLast	public	TSingleIndexTree	
MoveNext	public	TSingleIndexTree	
MovePrevious	public	TSingleIndexTree	
Seek	public	TSingleIndexTree	

procedure Delete; virtual;
Deleta da árvore o nó atual;

function GetFreeCode: integer;
Retorna um código que pode ser utilizado em um novo nó de forma a não coincidir com os nós já existentes;

procedure Insert(c: integer); virtual;
Insere um novo nó. Nesta árvore, o nó é simplesmente um código qualquer. Os  nós são ordenados por "c";

procedure MoveFirst; virtual;
Move-se para o primeiro nó da árvore, de acordo com o índice atual (esta árvore possui somente um índice, por chave, mas suas descendentes podem ter outros);

procedure MoveLast; virtual;
Move-se para o último nó da árvore, de acordo com o índice atual (esta árvore possui somente um índice, por chave, mas suas descendentes podem ter outros);

procedure MoveNext; virtual;
Move-se para o próximo nó da árvore, de acordo com o índice atual (esta árvore possui somente um índice, por chave, mas suas descendentes podem ter outros);

procedure MovePrevious; virtual;
Move-se para o nó anterior da árvore, de acordo com o índice atual (esta árvore possui somente um índice, por chave, mas suas descendentes podem ter outros);

procedure Seek(key: integer); virtual;
Procura por um nó de código "key". Se bem-sucedido, a propriedade Blind é definida como falso. Caso contrário, como verdadeiro;


TTails
Unidade: ExDataSt
TObject  TSingleIndexTree  TTails

Descrição
	
Representa as caudas (premissas) de uma regra. De acordo com o que foi descrito no começo desta seção, uma premissa é formada por quatro atributos: código da variável + operador + código do valor (ou fórmula matemática) + conectivo para próxima premissa.

Propriedades

Propriedade	Visibilidade	Default	Documentada em	
Blind	public		TSingleIndexTree	
Code	public		TTails	
Conect	public		TTails	
Neg	public		TTails	
Operator	public		TTails	
RecordCount	public		TsingleIndexTree	
ValCode	public		TTails	
      VarCode	public		TTails	

Code: integer
Código da regra associada. A ordem das premissas é definida pela ordem na qual elas aparecem na árvore. Ao usarmos o método Seek(x), o nó corrente passa a ser a primeira premissa da regra de código x. Com o método MoveNext, é possível iterar por todas as premissas;

Conect: integer
Tipo de conectivo que liga a premissa corrente à próxima premissa. A última premissa tem como conectivo o valor ENTÃO. Os valores de conectivo são: cnAND (E: valor 0), cnOR (OU: valor 1), cnTHEN (ENTÃO: valor 2);

Neg: boolean
Quando verdadeira, inverte o sentido do operador ("=" é interpretado com "<>", "<" como ">=", etc.)

Operator: string
Operador que especifica a o tipo de comparação entre uma variável e um valor. Os operadores possíveis são "=", ">", "<", ">=", "<=", "<>"

ValCode: integer
Código do valor (ou fórmula matemática) que está sendo comparada com uma variável;

VarCode: integer
Código da variável que está sendo comparada com um valor;

Métodos

Método	Visibilidade	Documentado em	
Delete	public	TSingleIndexTree	
GetFreeCode	public	TSingleIndexTree	
Insert	public	TTails	
MoveFirst	public	TSingleIndexTree	
MoveLast	public	TSingleIndexTree	
MoveNext	public	TSingleIndexTree	
MovePrevious	public	TSingleIndexTree	
Seek	public	TSingleIndexTree	

procedure Insert(c: integer; c_var: integer; op: string; c_val: integer; conec: integer; neg: boolean);
Insere um novo nó, onde "c" é o código da regra relacionada, "c_var" o código da variável em comparação, "op" o operador de comparação, "c_val" o código do valor (ou fórmula) comparada, "conec" o conectivo para próxima premissa e "neg" o indicativo de negação. 


TTargetNode
Unidade: ExDataSt
TObject  TTargetNode

Descrição
	
Os items da lista TTargets são objetos do tipo TTargetNode. Estes items guardam informações sobre os objetivos do sistema especialista, enquanto TTargets define sua ordem de busca.

Propriedades

Propriedade	Visibilidade	Default	Documentada em	
Code	public		TTargetNode	
ShowResults	public		TTargetNode	

Code: integer
O código da variável relacionada;

ShowResults: boolean
Se falso, o evento OnShowResults não é disparado quando esse objetivo é encontrado;


TTargets
Unidade: ExDataSt
TObject  TList  TTargets

Descrição
	
Os objetivos do sistema especialista. Note que não se usa uma árvore ordenada, pois os itens devem estar em uma lista cuja ordem depende apenas da ordem de entrada.

Propriedades

Propriedade	Visibilidade	Default	Documentada em	
Capacity	public		TList	
Count	public		TList	
Items	public		TList	
List	public		TList	
Nodes	public		TTargets	

Nodes[Index: integer]: TTargetNode
Lê os valores da lista. Use no lugar da propriedade Items (usada para as outras operações: deleção, inserção, etc.) para ler valores diretamente como TTargetNode, sem necessidade de casting;

Para informações sobre os métodos, consulte a documentação de TList no Borland Delphi.


TValues
Unidade: ExDataSt
TObject  TSingleIndexTree  TCodeNameIndexTree  TValues

Descrição
	
A representação dos valores relacionados às variáveis. Os valores especiais são UNKNOWN (Desconhecido: valor -1), YES (Sim: -2), NO (Não: -3). Os nós aqui são ordenados tanto pelo código e nome como também pelo código da variável relacionado a este valor. Para maiores detalhes, consulte as classes de base e os comentários no começo deste capítulo.

Propriedades

Propriedade	Visibilidade	Default	Documentada em	
Blind	public		TSingleIndexTree	
Code	public		TSingleIndexTree	
CurrentKey	public	PRIMARY_KEY	TValues	
Name	public		TCodeNameIndexTree	
Position	public		TValues	
RecordCount	public		TSingleIndexTree	
VarCode	public		Tvalues	

CurrentKey: integer
Índice atual usado pelos métodos Move... e pelas propriedades. Os valores possíveis são: PRIMARY_KEY, BY_NAME e BY_VAR;

Position: integer
Lembrando que o nó atual representa um valor do Expert SINTA, essa propriedade indica a posição na qual o item do valor aparece em um TExpertPrompt;

VarCode: integer
Relaciona o nó atual (que representa um valor) a uma variável;

Métodos

Método	Visibilidade	Documentado em	
Delete	public	TsingleIndexTree	
GetFreeCode	public	TsingleIndexTree	
Insert	public	Tvalues	
MoveFirst	public	TsingleIndexTree	
MoveLast	public	TsingleIndexTree	
MoveNext	public	TsingleIndexTree	
MovePrevious	public	TsingleIndexTree	
Seek	public	Tvalues	
SeekByName	public	Tvalues	
SeekByVar	public	Tvalues	

procedure Insert(c: integer; name: string; c_var: integer; position: integer); 
Insere um novo nó. Nesta árvore, o nó representa uma variável (código + nome + código da variável a qual pertence + posição no qual deve aparecer em um TExpertPrompt). Os  nós são ordenados por "c", "name" ou "c_var";

procedure Seek(key: integer); 
Encontrar o primeiro nó de código "key". Se bem-sucedido, a propriedade Blind é definida como falso. Caso contrário, como verdadeiro. O índice corrente é mudado para PRIMARY_KEY, índice por código;

procedure SeekByName(name: string; v: integer); 
Procura por um nó de nome "name", pertecente à variável "v". Se bem-sucedido, a propriedade Blind é definida como falso. Caso contrário, como verdadeiro. O índice corrente é mudado para BY_NAME, índice por nome;

procedure SeekByVar(c_var: integer); 
Encontrar o primeiro nó cujo código de variável é "c_var". O índice corrente é mudado para BY_VAR, índice por código de variável. Itere pela árvore usando MoveNext para encontrar o valor desejado, identificando-o pelas demais propriedades;


TVarBasedEvent
Unidade: ExSystem
Este tipo de dado é um procedure, não uma classe

Descrição
	
Este tipo de procedure é utilizada para determinados eventos existentes na classe TExpertSystem. O primeiro parâmetro corresponde ao parâmetro padrão de um evento Delphi: o objeto que disparou o evento. O segundo parâmetro é uma informação complementar, cujo significado dependerá do evento em si. Para ver exemplos de utilização de TVarBasedEvent, consulte a documentação da classe TExpertSystem no próximo capítulo.

{Eventos relativos ao sistema especialista, onde V é um código de
 variável}
TVarBasedEvent = procedure (Sender: TObject; V: integer) 
                 of object;


TVarListExtraTree
Unidade: ExDataSt
TObject  TSingleIndexTree  TVarListExtraTree

Descrição
	
As instâncias das variáveis durante uma consulta são guardadas em duas árvores: VarListTree e VarListExtraTree. A segunda guarda as instâncias de valores não representados por códigos (não estão na árvore Values nem são do tipo Sim / Não / DESCONHECIDO). Até o momento, os únicos valores deste tipo que o Expert SINTA suporta são valores numéricos. Valores DESCONHECIDO para variáveis numéricas são guardadas em VarListTree.

Propriedades

Propriedade	Visibilidade	Default	Documentada em	
Blind	public		TSingleIndexTree	
Code	public		TSingleIndexTree	
CNF	public		TVarListExtraTree	
RecordCount	public		TSingleIndexTree	
Value	public		TVarListExtraTree	

Code: integer
Código de uma variável. Para cada instância que uma variável possui, existe um nó correspondente na árvore. Com o método Seek encontra-se a primeira instância de uma variável. Como MoveNext, itera-se pelos nós para encontrar outras possíveis instâncias;

CNF: single
O grau de confiança da instância;

Value: string
O valor atribuído. Seu significado depende da variável associada. Com variáveis numéricas, note que é necessário converter de string para real sempre que necessário;

Métodos

Método	Visibilidade	Documentado em	
Delete	public	TSingleIndexTree	
GetFreeCode	public	TSingleIndexTree	
Insert	public	TVarListExtraTree	
MoveFirst	public	TSingleIndexTree	
MoveLast	public	TSingleIndexTree	
MoveNext	public	TSingleIndexTree	
MovePrevious	public	TSingleIndexTree	
Seek	public	TSingleIndexTree	

procedure Insert(c: integer; v: string; cnf: single);
Insere um novo nó, onde "c" é o código da variável, "v" é o valor (instância), "cnf" é o grau de confiança da instância;


TVarListTree
Unidade: ExDataSt
TObject  TSingleIndexTree  TVarListTree

Descrição
	
As instâncias das variáveis durante uma consulta são guardadas em duas árvores: VarListTree e VarListExtraTree. A primeira guarda as instâncias de valores que estão presentes na árvore Values e valores Sim/Não, além do valor DESCONHECIDO.

Propriedades

Propriedade	Visibilidade	Default	Documentada em	
Blind	public		TSingleIndexTree	
Code	public		TVarListTree	
CNF	public		TVarListTree	
RecordCount	public		TSingleIndexTree	
ValCode	public		TVarListTree	

Code: integer
Código de uma variável. Para cada instância que uma variável possui, existe um nó correspondente na árvore. Com o método Seek encontra-se a primeira instância de uma variável. Como MoveNext, itera-se pelos nós para encontrar outras possíveis instâncias;

CNF: single
O grau de confiança da instância;

ValCode: integer
O código do valor relacionado à variável, ou seja, a instância da variável;

Métodos

Método	Visibilidade	Documentado em	
Delete	public	TSingleIndexTree	
GetFreeCode	public	TSingleIndexTree	
Insert	public	TVarListTree	
MoveFirst	public	TSingleIndexTree	
MoveLast	public	TSingleIndexTree	
MoveNext	public	TSingleIndexTree	
MovePrevious	public	TSingleIndexTree	
Seek	public	TSingleIndexTree	

procedure Insert(c: integer; v: integer; cnf: single);
Insere um novo nó, onde "c" é o código da variável, "v" é o código do valor (instância), "cnf" é o grau de confiança da instância;


TVars
Unidade: ExDataSt
TObject  TSingleIndexTree  TCodeNameIndexTree  TVars

Descrição
	
A representação das variáveis. Para maiores detalhes, consulte as classes de base e os comentários no começo deste capítulo.

Propriedades

Propriedade	Visibilidade	Default	Documentada em	
Blind	public		TSingleIndexTree	
Code	public		TSingleIndexTree	
CurrentKey	public	PRIMARY_KEY	TCodeNameIndexTree	
Multi	public		TVars	
Name	public		TCodeNameIndexTree	
Numeric	public		TVars	
RecordCount	public		TSingleIndexTree	

Multi: boolean
Propriedade que indica se o nó corrente da árvore representa uma variável multivalorada ou  não;

Numeric: boolean
Propriedade que indica se o nó corrente da árvore representa uma variável numérica ou  não;

Métodos

Método	Visibilidade	Documentado em	
Delete	public	TSingleIndexTree	
GetFreeCode	public	TSingleIndexTree	
Insert	public	TVars	
MoveFirst	public	TSingleIndexTree	
MoveLast	public	TSingleIndexTree	
MoveNext	public	TSingleIndexTree	
MovePrevious	public	TSingleIndexTree	
Seek	public	TCodeNameIndexTree	
SeekByName	public	TCodeNameIndexTree	

procedure   Insert(c: integer; name: string; num: boolean; multi: boolean);
Insere um novo nó. Nesta árvore, o nó representa uma variável (código + nome + indicativo de numérica ou não + indicativo de multivalorada ou não). Os  nós são ordenados por "c" ou "name";


	


Capítulo
8


Os Componentes Visuais


Nesta seção, são descritos os componentes visuais acessíveis pelo ambiente de programação utilizado na construção de sistemas especialistas baseados na Expert SINTA VCL. De cada componente (classe), são comentados propriedades, métodos e eventos, onde a nomenclatura procura seguir as convenções estabelecidas no Object Pascal. Vale também lembrar que esta documentação refere-se à Expert SINTA VCL para Win32 (Delphi 2.0 ou superior). Componentes para Windows 3.1 podem variar um pouco dos aqui descritos. Classes de interface (TExCtrlInterface, TExDebugInterface) são descritas em detalhes no capítulo 6.
Outra observação: nem todas as propriedades, métodos e eventos herdados de componentes da VCL do Delphi são apresentados aqui. Para uma lista completa, consulte a documentação das classes base de cada componente da Expert SINTA VCL na documentação do Borland Delphi.

As Classes

	As classes seguintes são apresentadas em ordem alfabética, mas é bom sempre lembrar que todos os componentes, de alguma forma, estão relacionados a TExpertSystem:

TAllVars 
Unidade: ExCtrls
TObject  TPersistent  TComponent  TControl  TWinControl  TCustomTreeView  TAllVars

Descrição
	
Para exibir todos valores de todas as variáveis, use este componente. Note que variáveis ainda não instanciadas não são apresentadas. DICA: pode-se usar este componente como alternativa ao TWatchPanel. Use o evento OnChange de TExpertSystem para atualizá-lo (mais o desempenho não é o mesmo, pois toda a árvore é redesenhada).

Propriedades

Propriedade	Visibilidade	Default	Documentada em	
Align	published	alNone	TControl	
BorderStyle	published	bsSingle	TCustomListBox	
Color	published	clWindow	TWinControl	
Ctrl3D	published	True	TWinControl	
Cursor	published	crDefault	TControl	
DragCursor	published	crDrag	TControl	
DragMode	published	dmManual	TControl	
Enabled	published	True	TControl	
Font	published		TControl	
Height	published		TControl	
HelpContext	published	0	TWinControl	
HideSelection	published	True	TCustomTreeView	
Hint	published		TControl	
Items	published		TCustomTreeView	
Left	published		TControl	
Name	published		TComponent	
ParentColor	published	False	TControl	
ParentCtrl3D	published	True	TWinControl	
ParentFont	published	True	TControl	
ParentShowHint	published	True	TControl	
PopupMenu	published		TControl	
ReadOnly	published	True	TCustomTreeView	
ShowButtons	published	True	TCustomTreeView	
ShowHint	published	False	TControl	
ShowLines	published	True	TCustomTreeView	
ShowRoot	published	True	TCustomTreeView	
TabOrder	published		TWinControl	
TabStop	published	False	TWinControl	
Tag	published	0	TComponent	
Top	published		TControl	
Visible	published	True	TControl	
Width	published		TControl	

Métodos

Método	Visibilidade	Documentado em	
CreateTree	public	TAllVars	
Demais métodos	public/protected	TCustomTreeView	

procedure CreateTree(ES: TExpertSystem)
Preenche o objeto com a árvore representando todos os valores e variáveis;

Demais métodos
Consulte a documentação do Delphi/C++ Builder;

Eventos

Eventos	Visibilidade	Documentado em	
OnChange	published	TCustomTreeView	
OnChanging	published	TCustomTreeView	
OnClick	published	TControl	
OnCollapsed	published	TCustomTreeView	
OnCollapsing	published	TCustomTreeView	
OnCompare	published	TCustomTreeView	
OnDblClick	published	TControl	
OnDeletion	published	TCustomTreeView	
OnDragDrop	published	TControl	
OnDragOver	published	TControl	
OnEndDrag	published	TControl	
OnEnter	published	TWinControl	
OnExit	published	TWinControl	
OnExpanded	published	TCustomTreeView	
OnExpanding	published	TCustomTreeView	
OnGetImageIndex	published	TCustomTreeView	
OnGetSelectedIndex	published	TCustomTreeView	
OnKeyDown	published	TWinControl	
OnKeyPress	published	TWinControl	
OnKeyUp	published	TWinControl	
OnMouseDown	published	TControl	
OnMouseMove	published	TControl	
OnMouseUp	published	TControl	
OnStartDrag	published	TControl	


TConsultTree
Unidade: ExCtrls
TObject  TPersistent  TComponent  TControl  TWinControl  TCustomTreeView  TConsultTree 

Descrição
	
É neste componente que se exibem os passos realizados por uma consulta. Normalmente, no evento OnShowResults de um dado TExpertSystem, um objeto destes recebe, através do método CreateTree, informações para criar uma árvore de pesquisa.

Propriedades

Propriedade	Visibilidade	Default	Documentada em	
Align	published	alNone	TControl	
BorderStyle	published	bsSingle	TCustomListBox	
Color	published	clWindow	TWinControl	
Ctrl3D	published	True	TWinControl	
Cursor	published	crDefault	TControl	
DragCursor	published	crDrag	TControl	
DragMode	published	dmManual	TControl	
Enabled	published	True	TControl	
Font	published		TControl	
Height	published		TControl	
HelpContext	published	0	TWinControl	
HideSelection	published	True	TCustomTreeView	
Hint	published		TControl	
Left	published		TControl	
Name	published		TComponent	
ParentColor	published	False	TControl	
ParentCtrl3D	published	True	TWinControl	
ParentFont	published	True	TControl	
ParentShowHint	published	True	TControl	
PopupMenu	published		TControl	
ReadOnly	published	True	TCustomTreeView	
ShowButtons	published	True	TCustomTreeView	
ShowHint	published	False	TControl	
ShowLines	published	True	TCustomTreeView	
ShowRoot	published	True	TCustomTreeView	
TabOrder	published		TWinControl	
TabStop	published	False	TWinControl	
Tag	published	0	TComponent	
Top	published		TControl	
Visible	published	True	TControl	
Width	published		TControl	

Métodos

Método	Visibilidade	Documentado em	
Clear	public	TConsultTree	
CreateTree	public	TConsultTree	
Demais métodos	public/protected	TCustomTreeView	

procedure CreateTree(ES: TExpertSystem)
Preenche o objeto com a árvore de consulta. ATENÇÃO: uma vez descarregadas as informações sobre o histórico da consulta, elas se perderão. Se precisar exibir a árvore em mais de uma TConsultTree, copie os nós como faria em um TCustomOutline (Win16) ou TCustomTreeView (Win32);

procedure Clear
Limpa todo o conteúdo da árvore;

Demais métodos
Consulte a documentação do Delphi/C++ Builder;

Eventos

Eventos	Visibilidade	Documentado em	
OnChange	published	TCustomTreeView	
OnChanging	published	TCustomTreeView	
OnClick	published	TControl	
OnCollapsed	published	TCustomTreeView	
OnCollapsing	published	TCustomTreeView	
OnCompare	published	TCustomTreeView	
OnDblClick	published	TControl	
OnDeletion	published	TCustomTreeView	
OnDragDrop	published	TControl	
OnDragOver	published	TControl	
OnEndDrag	published	TControl	
OnEnter	published	TWinControl	
OnExit	published	TWinControl	
OnExpanded	published	TCustomTreeView	
OnExpanding	published	TCustomTreeView	
OnGetImageIndex	published	TCustomTreeView	
OnGetSelectedIndex	published	TCustomTreeView	
OnKeyDown	published	TWinControl	
OnKeyPress	published	TWinControl	
OnKeyUp	published	TWinControl	
OnMouseDown	published	TControl	
OnMouseMove	published	TControl	
OnMouseUp	published	TControl	
OnStartDrag	published	TControl	


TDebugPanel
Unidade: ExCtrls
TObject  TPersistent  TComponent  TControl  TWinControl  TCustomListBox  TCustomRuleView  TDebugPanel

Descrição
	
Uma listbox que funciona como depurador, exibindo como item destacado o ponto no qual a máquina de inferência está durante uma consulta. O controle do depurador é feito pelo objeto TExpertSystem associado (métodos Pause, Resume e ExecuteStep).

Propriedades

Propriedade	Visibilidade	Default	Documentada em	
Align	published	alNone	TControl	
BorderStyle	published	bsSingle	TCustomListBox	
Color	published	clWindow	TWinControl	
Ctrl3D	published	True	TWinControl	
Cursor	published	crDefault	TControl	
DragCursor	published	crDrag	TControl	
DragMode	published	dmManual	TControl	
Enabled	published	True	TControl	
ExpertSystem	published		TDebugPanel	
ExtendedSelect	published	True	TCustomListBox	
Font	published		TControl	
Height	published		TControl	
HelpContext	published	0	TWinControl	
Hint	published		TControl	
IntegralHeight	published	False	TCustomListBox	
ItemHeight	published		TCustomListBox	
Left	published		TControl	
MultiSelect	published	False	TCustomListBox	
Name	published		TComponent	
ParentColor	published	False	TControl	
ParentCtrl3D	published	True	TWinControl	
ParentFont	published	True	TControl	
ParentShowHint	published	True	TControl	
PopupMenu	published		TControl	
ShowHint	published	False	TControl	
TabOrder	published		TWinControl	
TabStop	published	False	TWinControl	
TabWidth	published	0	TCustomListBox	
Tag	published	0	TComponent	
Top	published		TControl	
Visible	published	True	TControl	
Width	published		TControl	

ExpertSystem: TExpertSystem
Sistema especialista cuja máquina de inferência será acompanhada;

Métodos

Método	Visibilidade	Documentado em	
Activate	public	TDebugPanel	
FillRules	public	TRuleView	
SetHorizontalBar	public	TRuleView	
Update	public	TDebugPanel	
Demais métodos	public/protected	TCustomListBox	

procedure Activate
Aciona o depurador, ocasionando espera na máquina de inferência. Este método é chamado pelo sistema especialista associado através de uma interface do tipo TDebugPanelInterface;

procedure Update(rule: integer; order: integer; is_tail: boolean)
Indica ao depurador que a máquina de inferência está na regra de código "rule", no item de número "order", onde "is_tail" indica se este item está nas conclusões (cabeças de regra - "is_tail" é falso ) ou conclusões (caudas de regra - "is_tail" é verdadeiro). Este método é chamado pelo sistema especialista associado através de uma interface do tipo TDebugPanelInterface;

Demais métodos
Consulte a documentação do Delphi/C++ Builder

Eventos

Eventos	Visibilidade	Documentado em	
OnClick	published	TControl	
OnDblClick	published	TControl	
OnDragDrop	published	TControl	
OnDragOver	published	TControl	
OnEndDrag	published	TControl	
OnEnter	published	TWinControl	
OnExit	published	TWinControl	
OnKeyDown	published	TWinControl	
OnKeyPress	published	TWinControl	
OnKeyUp	published	TWinControl	
OnMouseDown	published	TControl	
OnMouseUp	published	TControl	
OnStartDrag	published	TControl	


TExNavigator 
Unidade: ExCtrls
TObject  TPersistent  TComponent  TControl  TWinControl  TCustomControl  TConsultPanel  TExNavigator

Descrição
	
O navegador TExNavigator exibe botões que permitem mudar o status da consulta (por exemplo, iniciá-la, voltar para a pergunta anterior, dar uma pausa, finalizar). Os botões são disponibilizados ou não de acordo com o status da máquina de inferência.
	DICA: para construir um navegador personalizado, pode utilizar controles (botões, por exemplo) para disparar métodos como Pause, StartEngine, etc. no objeto TExpertSystem desejado e eventos como OnPause, OnStart para disponibilizar, ou inibir, esses controles.

Propriedades

Propriedade	Visibilidade	Default	Documentada em	
Align	published	alNone	TControl	
BevelInner	published	bvNone	TCustomPanel	
BevelOuter	published	bvRaised	TCustomPanel	
BevelWidth	published	1	TCustomPanel	
BorderStyle	published	bsSingle	TCustomListBox	
BorderWidth	published	0	TCustomListBox	
btnBack	public		TExNavigator	
btnGo	public		TExNavigator	
btnPause	public		TExNavigator	
btnStep	public		TExNavigator	
btnStop	public		TExNavigator	
Color	published	clWindow	TWinControl	
Ctrl3D	published	True	TWinControl	
Cursor	published	crDefault	TControl	
DragCursor	published	crDrag	TControl	
DragMode	published	dmManual	TControl	
Enabled	published	True	TControl	
ExpertSystem	published		TExNavigator	
Height	published		TControl	
HelpContext	published	0	TWinControl	
Hint	published		TControl	
Left	published		TControl	
Locked	published	False	TCustomPanel	
Name	published		TComponent	
ParentColor	published	False	TControl	
ParentCtrl3D	published	True	TWinControl	
PopupMenu	published		TControl	
TabOrder	published		TWinControl	
TabStop	published	False	TWinControl	
Tag	published	0	TComponent	
Top	published		TControl	
Visible	published	True	TControl	
VisibleButtons	published	[nbGo, nbBack, nbStep, nbPause, nbStop]	TExNavigator	
Width	published		TControl	

btnGo, btnBack, btnStep, btnPause, btnStop: TSpeedButton
São os botões que correspondem às funções, respectivamente, de Iniciar/Continuar, Voltar para a pergunta anterior, Executar passo, Pausa, Abortar consulta;

ExpertSystem: TExpertSystem
O sistema associado ao navegador;

VisibleButtons: TNavigatorVisibleButtons ([nbGo, nbBack, bnStep, nbPause, nbStop])
Se você não desejar, por exemplo, que o botão de pausa e passo não apareçam, use essa propriedade;

Métodos

Método	Visibilidade	Documentado em	
DisableAll	public	TExNavigator	
UpdateButtons	public	TExNavigator	
SetDefaults	public	TExNavigator	
Demais métodos	public/protected	TCustomPanel	

procedure DisableAll
Desabilita todos os botões;

procedure UpdateButtons
Abilita/desabilita os botões de acordo com o estado da máquina de inferência;

procedure SetDefaults
Abilita/desabilita os botões para o estado de partida da máquina de inferência;

Demais métodos
Consulte a documentação do Delphi/C++ Builder;

Eventos

Eventos	Visibilidade	Documentado em	
OnDragDrop	published	TControl	
OnDragOver	published	TControl	
OnEndDrag	published	TControl	
OnEnter	published	TWinControl	
OnExit	published	TWinControl	
OnMouseDown	published	TControl	
OnMouseMove	published	TControl	
OnMouseUp	published	TControl	
OnResize	published	TCustomPanel	
OnStartDrag	published	TControl	


TExpertPrompt
Unidade: ExCtrls
TObject  TPersistent  TComponent  TControl  TWinControl  TScrollingWinControl  TScrollBox  TExpertPrompt

Descrição
	
Monta um menu de checkboxes para entrada de dados automaticamente baseado em uma variável de uma base de conhecimento. Também atualiza automaticamente as instâncias da variável em uma consulta de acordo com o que foi marcado pelo usuário.

Propriedades

Propriedade	Visibilidade	Default	Documentada em	
Align	published	alNone	TControl	
AutoScroll	published	True	TScrollingWinControl	
AutoSize	published	False	TExpertPrompt	
BorderStyle	published	bsSingle	TCustomListBox	
BoundsRect	public		TControl	
Brush	public, read-only		TWinControl	
ClientHeight	public		TControl	
ClientOrigin	public, read-only		TWinControl	
ClientRect	public, read-only		TWinControl	
ClientWidth	public		TControl	
Color	published	clWindow	TWinControl	
ControlCount	public, read-only		TWinControl	
Controls	public, read-only		TWinControl	
Ctrl3D	published	True	TWinControl	
Cursor	published	crDefault	TControl	
DragCursor	published	crDrag	TControl	
DragMode	published	dmManual	TControl	
Enabled	published	True	TControl	
ExpertSystem	published		TExpertPrompt	
Font	published		TControl	
Height	published		TControl	
HelpContext	published	0	TWinControl	
Hint	published		TControl	
HorzScrollBar	published		TScrollingWinControl	
Left	published		TControl	
MaxHeight	published	300	TExpertPrompt	
MaxWidth	published	400	TExpertPrompt	
MinHeight	published	0	TExpertPrompt	
MinWidth	published	0	TExpertPrompt	
Name	published		TComponent	
Parent	public		TControl	
ParentColor	published	False	TControl	
ParentCtrl3D	published	True	TWinControl	
ParentFont	published	True	TControl	
ParentShowHint	published	True	TControl	
PopupMenu	published		TControl	
ShowErrorMessages	published		TExpertPrompt	
ShowHint	published	False	TControl	
TabOrder	published		TWinControl	
TabStop	published	False	TWinControl	
TabWidth	published	0	TCustomListBox	
Tag	published	0	TComponent	
Top	published		TControl	
VarCode	published	0	TExpertPrompt	
VertScrollBar	published		TScrollingWinControl	
Visible	published	True	TControl	
Width	published		TControl	

AutoSize: boolean
Quando verdadeira, o prompt ajusta automaticamente seu tamanho de acordo com as opções apresentadas;

ExpertSystem: TExpertSystem
O sistema que fornece a base de conhecimento ao ExpertPrompt;

MaxHeight: integer
Altura máxima que o prompt pode atingir quando AutoSize é verdadeira;

MaxWidth: integer
Largura máxima que o prompt pode atingir quando AutoSize é verdadeira;

MinHeight: integer
Altura mínima que o prompt pode atingir quando AutoSize é verdadeira;

MinWidth: integer
Largura mínima que o prompt pode atingir quando AutoSize é verdadeira;

ShowErrorMessages: boolean
Quando verdadeira, o ExpertPrompt exibirá mensagem de erro em caso de entrada inválida;

VarCode: integer
Variável que irá fornecer os valores para a construção das opções;

Métodos

Método	Visibilidade	Documentado em	
ArrangeTextOptions	public	TExpertPrompt	
BuildOptions	public	TExpertPrompt	
UpdateBase	public	TExpertPrompt	
Demais métodos	public/protected	TScrollBox	

procedure ArrangeTextOptions
Semelhante a BuildOptions, mais é usado apenas para os menus de opções de checkboxes (ou seja, para entradas de dados não numéricas). Sua função é basicamente reposicionar os controles dentro do TExpertPrompt. É um pouco mais rápido;

procedure BuildOptions
Força a exibição de opções no TExpertPrompt (é automática quando se muda a propriedade ExpertSystem ou VarCode). Pode ser usada para, por exemplo, reposicionar os controles filhos quando mudamos o tamanho do TExpertPrompt.;

procedure UpdateBase
Atualiza a consulta com os valores dados como entrada nas opções do prompt.

Demais métodos
Consulte a documentação do Delphi/C++ Builder

Eventos

Eventos	Visibilidade	Documentado em	
OnClick	published	TControl	
OnDblClick	published	TControl	
OnDragDrop	published	TControl	
OnDragOver	published	TControl	
OnEndDrag	published	TControl	
OnEnter	published	TWinControl	
OnExit	published	TWinControl	
OnMouseDown	published	TControl	
OnMouseMove	published	TControl	
OnMouseUp	published	TControl	
OnResize	published	TScrollBox	


TExpertSystem 
Unidade: ExSystem
TObject  TPersistent  TComponent  TExpertSystem

Descrição
	
Classe principal, onde estão incluídas a máquina de inferência e a estrutura de dados que representa a base de conhecimento. Os outros componentes da Expert SINTA VCL sempre referenciam algum objeto TExpertSystem direta ou indiretamente.

Propriedades

Propriedade	Visibilidade	Default	Documentada em	
ExecutionMode	public		TExpertSystem	
FileName	published		TExpertSystem	
Formulas	public		TExpertSystem	
Heads	public		TExpertSystem	
HelpContexts	public		TExpertSystem	
Info	public		TExpertSystem	
Name	published		TComponent	
Questions	public		TExpertSystem	
ReportEnding	published	True	TExpertSystem	
Rules	public		TExpertSystem	
Tag	published	0	TComponent	
Tails	public		TExpertSystem	
Targets	public		TExpertSystem	
Trace	public		TExpertSystem	
Trail	public		TExpertSystem	
Values	public		TExpertSystem	
VarList	public		TExpertSystem	
VarListExtra	public		TExpertSystem	
Vars	public		TExpertSystem	
WaitingAnswer	public		TExpertSystem	

ExecutionMode: boolean
Se verdadeira, indica que uma consulta está em andamento. Quando falsa, a máquina de inferência não está sendo utilizada e, portanto, não há nenhuma consulta em andamento, significando que muitas operações, como criar instâncias de variáveis, não podem ser efetuadas.

FileName: string
Indica o nome do arquivo BCM correspondente à base do sistema especialista utilizado. Quando o valor desta propriedade é mudado, a base é imediatamente aberta. Se outra estava sendo utilizada, será fechada automaticamente;

Formulas: TFormulas
Estrutura de dados representando as fórmulas matemáticas utilizadas nas regras. Para maiores informações, consulte o capítulo anterior;

Heads: THeads
Estrutura de dados representando as conclusões de cada regra. Para maiores informações, consulte o capítulo anterior;

HelpContexts: THelpContexts
Estrutura de dados representando os valores de contexto de ajuda ligados aos valores das variáveis-objetivo. Para maiores informações, consulte o capítulo anterior;

Info: TInfos
Informações sobre a base. Para maiores informações, consulte o capítulo anterior;

Questions: TQuestions
Estrutura de dados representando as mensagens utilizadas como perguntas ao usuário final. Para maiores informações, consulte o capítulo anterior;

ReportEnding: boolean
Se verdadeira, uma mensagem aparecerá ao final de uma consulta indicando seu término ("Fim da Consulta"). Se desejar que esta mensagem não apareça, atribua false a esta propriedade;

Rules: TRules
Estrutura de dados representando as regras como um todo. Para maiores informações, consulte o capítulo anterior;

Tails: TTails
Estrutura de dados representando as premissas de cada regra. Para maiores informações, consulte o capítulo anterior;

Targets: TTargets
Estrutura de dados representando a lista de variáveis-objetivo a serem procuradas. Para maiores informações, consulte o capítulo anterior;

Trace: boolean
Indica se a base está no modo de depuração;

Trail: TQueue
Fila de inteiros onde todas as operações realizadas pela máquina de inferência são registradas. Estas operações são armazenadas em duplas, ou seja, a cada operação realizada pela máquina de inferência corresponde a dois inteiros a mais adicionados ao final de Trail. O significado destes inteiros (digamos, cod_1_ e cod_2_) varia de acordo com as informações a seguir:

Status de cod_1_	Significado	
cod_1_ > 0	a máquina de inferência está passando pela premissa de ordem cod_2_ da regra cod_1_.	
cod_1_ = 0	a máquina de inferência realizou uma pergunta sobre a variável cod_2_.	
cod_1_ = -1	a regra cod_2_ foi aprovada.	
cod_1_ = -2	a regra cod_2_ foi rejeitada.	
cod_1_ = -3	a máquina de inferência iniciou a busca pela variável cod_2_.	

	Para melhor compreender o significado de Trail, veja o método AddTrail na unidade ExSystem (método não documentado aqui) e o código de TConsultTree. 

Values: TValues
Estrutura de dados representando os valores que uma variável pode assumir (não confundir com as instâncias assumidas por uma variável durante uma consulta). Para maiores informações, consulte o capítulo anterior.

VarList: TVarListTree
Lista de instâncias assumidas por variável univaloradas e multivaloradas. Inclui também instâncias DESCONHECIDAS de todos os tipos de variáveis. Você provalmente preferirá utilizar o método VarInstances do que lidar com esta propriedade diretamente;

VarListExtra: TVarListExtraTree
Lista de instâncias assumidas por variáveis numéricas. Não inclui instâncias DESCONHECIDAS. Você provalmente preferirá utilizar o método VarInstances do que lidar com esta propriedade diretamente;

Vars: TVars
Estrutura de dados representando as variáveis da base de conhecimento Para maiores informações, consulte o capítulo anterior;

WaitingAnswer: boolean
Quando verdadeira, paralisa a máquina de inferência no momento que uma pergunta deve ser feita. Utilizada para permitir entrada do usuário enquanto a máquina de inferência está em pausa;

Métodos

Método	Visibilidade	Documentado em	
AbortConsultation	public	TExpertSystem	
AttribVar	public	TExpertSystem	
AttribVarFromBinary	public	TExpertSystem	
AttribVarFromPosition	public	TExpertSystem	
BackToLastQuestion	public	TExpertSystem	
BookmarkVar	public	TExpertSystem	
ConectName	public	TExpertSystem	
DefaultPrompt	public	TExpertSystem	
ExecuteStep	public	TExpertSystem	
LoadFromFile	public	TExpertSystem	
PauseEngine	public	TExpertSystem	
RestoreVarFromBookmark	public	TExpertSystem	
ResumeEngine	public	TExpertSystem	
SaveToFile	public	TExpertSystem	
StartConsultation	public	TExpertSystem	
ValCode	public	TExpertSystem	
ValName	public	TExpertSystem	
ValuesList	public	TExpertSystem	
VarCode	public	TExpertSystem	
VarInstances	public	TExpertSystem	
VarName	public	TExpertSystem	

procedure  AbortConsultation
Se uma consulta está sendo efetuada, este método a cancelará;

procedure AttribVar(V: integer; Value: string; CNF: single; Is_a_code: boolean)
Inclui na lista de variáveis instanciadas a variável "V", bem como ajusta esses valores em caso de repetição ou quando a variável não é multivalorada. "Value" é o código do valor correspondente ou o valor numérico convertido para string. Códigos especiais, referentes a valores como SIM, NÃO e DESCONHECIDO são passados através de suas constantes convertidas para strings (por exemplo, IntToStr(YES), IntToStr(NO)). Se o valor sendo passado não deve ser interpretado com código de valor, mas como o próprio valor (o que é o normal com variáveis numéricas), o último parâmetro, "Is_a_code", deve ser falso. "CNF" é o grau de confiança da atribuição, variando de 0 a 1. Assim, se desejamos atribuir à variável 5 o valor de código 2 com grau de confiança 80%, invocamos este método do seguinte modo:

	AttribVar("5", "2", 0.8, true)

	Atribuir o valor SIM à variável 18, grau de confiança 100%:

	AttribVar("18", IntToStr(YES), 1, true)

	Atribuir 47,6 à variável numérica 39, grau de confiança 60%:

	AttribVar("39", "47.6", 0.6, false)

	Atribuir DESCONHECIDO à variável numérica 39, grau de confiança 100%

	AttribVar("39", IntToStr(UNKNOWN), 1, true)

procedure AttribVarFromBinary(V, VBin: integer)
Atribui diversos valores para uma dada variável "V". Estes valores estão identificados todos em um único número, o parâmetro "VBin". Isto permite a descrição de valores de uma consulta de forma compactada, conforme visto no exemplo do capítulo 4 deste documento.
Este método se baseia na "posição" relativa de cada valor em relação à variável. A posição de um valor pode ser visualizada e modificada no shell Expert SINTA: no editor de variáveis, simplesmente observe a lista de valores correspondente à variável selecionada. A ordem na qual eles aparecem na lista é a eqüivalente às suas posições (esta ordem é utilizada, por exemplo, em TExpertPrompt, o qual exibe opções de resposta na mesma ordem). Para mudar a posição dos valores, consulte a documentação do shell. 
	A figura a seguir, exibindo a janela de variáveis correspondente a um sistema de diagnóstico de pragas e doenças do cajueiro (SECAJU), deixa claro que, para a variável "doença", os valores "oidio", "antracnose" e "mofo preto" possuem posições 1, 2 e 3, respectivamente.


Figura 6 - Entrada de Variáveis no Shell

	Outra forma fácil de extrair as posições de cada valor é utilizar a opção ArquivoExportarCódigos... no shell.
Obs.:  Ao contrário dos códigos de variáveis, as posições podem mudar se o projetista da base de conhecimento assim desejar! Assim, fique certo de que estas mudanças não afetarão seu projeto.
Seguindo nosso exemplo, para atribuirmos o primeiro e o segundo valor à variável "doença", utilizamos o método AttribVarFromBinary do seguinte modo:

AttribVarFromBinary(2, 3)

O primeiro parâmetro diz respeito ao código da variável "doença" (2). O segundo é um valor montado a partir da combinação do primeiro e segundo valores. Esta combinação é baseada em dígitos binários. Os valores selecionados de "doença", dentre os valores disponíveis (três, no total) podem ser indicados pela seqüência 

011

que em números decimais eqüivale a 3. Da mesma forma, para uma variável com cinco valores, onde o segundo, o quarto e o quinto valores serão atribuídos, a seqüência eqüivalente é

11010

ou 25, em decimal. Para um bom exemplo de transformação do uso deste método, consulte o método UpdateBase de TListExpertPrompt da unidade de demonstração XLib comentada parcialmente nos capítulos 5 e 6 do presente documento. 

Obs.: Para atribuir os valores padrão DESCONHECIDO, SIM e NÃO, não utilize variáveis baseadas na posição, mas sim as constantes UNKNOWN, YES e NO, conforme presente na unidade ExConsts. Obviamente, este método não pode ser utilizado para variáveis numéricas, as quais não apresentam listas de valores (e suas respectivas posições).
Obs.: Por questões de simplicidade, este método não permite a escolha de um grau de confiança para cada valor atribuído. O valor 100% é assumido.

procedure AttribVarFromPosition(V, Pos: integer; CNF: single)
Uma forma variante de AttribVarFromBinary. Antes de compreender este método, leia as informações necessárias do método citado.
	Com AttribVarFromPosition, o valor situado na posição "Pos" é atribuído à variável "V" com o grau de confiança "CNF". Para obter a posição de uma variável, consulte o método AttribVarFromBinary.

procedure BackToLastQuestion
Volta para a última pergunta realizada pela máquina de inferência durante a busca ao objetivo atual;

procedure BookmarkVar
Guarda o estado atual das árvores internas de estruturas de dados Vars e Values em um "bookmark" interno. Somente um "bookmark" existe por sistema especialista. Com o método RestoreVarFromBookmark, as árvores Vars e Values são reposicionadas de acordo com a configuração salva.
	Cuidado ao usar BookmarkVar/RestoreVarFromBookmark. A boa prática é utilizá-los dentro de um mesmo procedimento, com a garantia de que as chamadas a outros métodos (entre a ação de guardar o status de Vars e Values no "bookmark" e a ação de restaurá-los) não invoquem novamente BookmarkVar. Por exemplo:

procedure Test(ExSys: TExpertSystem);
begin
   ExSys.Vars.MoveLast;
   ExSys.BookmarkVar;
   ShowMessage('Última variável: ' + ExSys.Vars.Name);
   ExSys.Vars.MoveFirst;
   ShowMessage('Primeira variável: ' + ExSys.Vars.Name);
   DoSomething(ExSys);
   ExSys.RestoreVarFromBookmark;
   ShowMessage('Última variável: ' + ExSys.Vars.Name);
end;

	O procedimento DoSomething citado acima não deve chamar o método BookmarkVar de ExSys sob a pena de que o último comando de Test não corresponda mais à realidade.

function ConectName(C: integer): string
Retorna o nome (string) corresponde ao código do conectivo passado como parâmetro. Este código corresponde às constantes cnAnd (E), cnOr (OU) e cnThen (ENTÃO) contidas na unidade ExConsts.

procedure DefaultPrompt(V: integer)
Exibe a entrada de dados default para a variável "V".

procedure ExecuteStep
Se a máquina de inferência estiver em pausa, executa um passo.

function LoadFromFile(FN: string)
Carrega uma base de conhecimento de arquivo BCM, cujo caminho é indicado pela string "FN". Retorna falso caso o carregamento não seja bem sucedido. Eqüivale à propriedade FileName.

procedure PauseEngine
Coloca a máquina de inferência no estado de pausa, modificando o valor da propriedade Trace para verdadeiro;

procedure RestoreVarFromBookmark
Coloca o estado das árvores Vars e Values de acordo com o que foi indicado no "bookmark" interno. Para maiores informações, consulte a documentação do método BookmarkVar desta mesma classe;

procedure ResumeEngine
Tira a máquina de inferência do estado de pausa, modificando o valor da propriedade Trace para falso;

function SaveToFile(FN: string)
Salva uma arquivo de base de conhecimento, cujo caminho é indicado pela string "FN". Nenhuma checagem de integridade referencial entre as árvores da estrutura de dados é feita (algo que o shell Expert SINTA, por exemplo, realiza, mas por outras formas). Retorna falso caso o salvamento não seja bem sucedido. 

procedure StartConsultation
Inicia a consulta.

functionValCode(Vr: integer; V: string): integer
Retorna o código do valor de nome "V" associado à variável de código "Vr".

functionValName(V: integer; C: integer): string
Retorna o nome do valor de código "C" associado à variável de código "V".

procedureValuesList(V: integer; ListOfValues: TStringList)
Preenche uma TStringList passada como parâmetro com a lista de valores possíveis de uma variável "V". Essa lista de valores é eqüivalente àquela que aparece quando selecionamos uma variável no editor de variáveis do shell Expert SINTA.

function VarCode(V: string): integer
Retorna o código de uma variável de nome "V".

procedure VarInstances(V: integer; ValuesList, CNFsList: TStringList)
Preenche uma TStringList passada como parâmetro (ValuesList) com todas as instâncias atuais de uma variável V em um dado instante de uma consulta, bem como seus respectivos graus de confiança em uma segunda TStringList (CNFsList). As instâncias são dispostas em ordem descrescente de grau de confiança e ordem crescente de posição em relação à variável (para saber mais de posições de um valor em uma variável, consulte o método AttribVarFromBinary desta mesma classe).
	Não se deve confundir uma lista de instâncias com uma lista de valores. Valores são fixos, determinados durante a construção da base de conhecimento e podem ser obtidos com o método ValuesList. Instâncias são os valores atribuídos a uma variável durante uma consulta. Portanto, só faz sentir invocar o método VarInstances se uma consulta estiver em andamento.

function VarName(C: integer): string
Retorna o nome de uma variável de código "C".

Eventos

Eventos	Visibilidade	Documentado em	
AfterCheckPassword	published	TExpertSystem	
OnChange	published	TExpertSystem	
OnExecuteStep	published	TExpertSystem	
OnFindTarget	published	TExpertSystem	
OnFinish	published	TExpertSystem	
OnPause	published	TExpertSystem	
OnPrompt	published	TExpertSystem	
OnResume	published	TExpertSystem	
OnShowResults	published	TExpertSystem	
OnStart	published	TExpertSystem	

AfterCheckPassword: TNotifyEvent
Evento chamado imediamente após a entrada de senha (quando necessária) e antes do carregamento do resto da base de conhecimento;

OnChange: TVarBasedEvent
Evento chamado quando uma instância é acrescentada (ou retirada) à variável V passada como parâmetro;

OnExecuteStep: TNotifyEvent
Evento chamado quando um passo é executado no modo de depuração;

OnFindTarget: TVarBasedEvent
Evento chamado imediatamente antes da busca de um dado objetivo passado como parâmetro. Útil para inicializar variáveis numa espécie de encadeamento para frente;

OnFinish: TNotifyEvent
Evento chamado logo após todas as finalizações necessárias da máquina de inferência. A consulta já foi completamente fechada antes deste evento ocorrer, inclusive com a destruição das instâncias existentes. Assim, se planeja utilizar algum resultado da consulta, é melhor fazê-lo pelo evento OnShowResults;

OnPause: TNotifyEvent
Evento chamado quando uma consulta entra no modo de depuração. Pode ser usado, por exemplo, para exibir um depurador;

OnPrompt: TVarBasedEvent
Evento chamado quando a máquina de inferência precisa saber do usuário o(s) valor(es) para a variável V passada como parâmetro. Um prompt padrão pode ser usado com o método DefaultPrompt;

OnResume: TNotifyEvent
Evento chamado quando a máquina de inferência sai do modo de depuração e volta para a consulta normal. Pode ser usado, por exemplo, para esconder um depurador;

OnShowResults: TVarBasedEvent
Evento chamado quando o objetivo (passado como parâmetro) é encontrado ou quando se esgotam todas as possibilidades de achá-lo;

OnStart: TNotifyEvent
Evento chamado imediamente antes da busca do primeiro objetivo. Útil para inicializar variáveis numa espécie de encadeamento para frente;


TLabelQuestion
Unidade: ExCtrls
TObject  TPersistent  TComponent  TControl  TGraphicControl  TCustomLabel  TLabelQuestion

Descrição
	
Label que exibe o texto de uma pergunta associada a uma variável.

Propriedades

Propriedade	Visibilidade	Default	Documentada em	
Align	published	alNone	TControl	
Alignment	published	taLeftJustify	TCustomLabel	
AutoSize	published	True	TCustomLabel	
Caption	published		TCustomLabel	
Color	published	clWindow	TWinControl	
Cursor	published	crDefault	TControl	
DragCursor	published	crDrag	TControl	
DragMode	published	dmManual	TControl	
Enabled	published	True	TControl	
ExpertSystem	published		TLabelQuestion	
FocusControl	published		TCustomLabel	
Font	published		TControl	
Height	published		TControl	
HelpContext	published	0	TWinControl	
Hint	published		TControl	
Left	published		TControl	
Name	published		TComponent	
ParentColor	published	False	TControl	
ParentFont	published	True	TControl	
ParentShowHint	published	True	TControl	
PopupMenu	published		TControl	
ShowAccelChar	published	True	TCustomLabel	
ShowHint	published	False	TControl	
Tag	published	0	TComponent	
Top	published		TControl	
Transparent	published	False	TCustomLabel	
VarCode	published	0	TLabelQuestion	
Visible	published	True	TControl	
Width	published		TControl	
WordWrap	published	False	TCustomLabel	

ExpertSystem: TExpertSystem
O sistema associado ao label;

VarCode: integer
O código da variável cuja pergunta será exibida;

Métodos

Método	Visibilidade	Documentado em	
BuildCaption	public	TLabelQuestion	
Demais métodos	public/protected	TCustomLabel	

procedure BuildCaption
Força a mudança de caption de acordo com a variável selecionada. Observação: o caption é mudado automaticamente já com a mudança da variável (propriedade VarCode);

Demais métodos
Consulte a documentação do Delphi/C++ Builder;

Eventos

Eventos	Visibilidade	Documentado em	
OnClick	published	TControl	
OnDblClick	published	TControl	
OnDragDrop	published	TControl	
OnDragOver	published	TControl	
OnEndDrag	published	TControl	
OnMouseDown	published	TControl	
OnMouseMove	published	TControl	
OnMouseUp	published	TControl	
OnStartDrag	published	TControl	


TRuleView
Unidade: ExCtrls
TObject  TPersistent  TComponent  TControl  TWinControl  TCustomListBox  TCustomRuleView  TRuleView

Descrição
	
Este componente é uma listbox que exibe as regras de uma determinada base de conhecimento associada a um componente TExpertSystem.

Propriedades

Propriedade	Visibilidade	Default	Documentada em	
Align	published	alNone	TControl	
BorderStyle	published	bsSingle	TCustomListBox	
Color	published	clWindow	TWinControl	
Ctrl3D	published	True	TWinControl	
Cursor	published	crDefault	TControl	
DragCursor	published	crDrag	TControl	
DragMode	published	dmManual	TControl	
Enabled	published	True	TControl	
ExpertSystem	published		TRuleView	
ExtendedSelect	published	True	TCustomListBox	
Font	published		TControl	
HeadFilter	public		TRuleView	
Height	published		TControl	
HelpContext	published	0	TWinControl	
Hint	published		TControl	
IntegralHeight	published	False	TCustomListBox	
ItemHeight	published		TCustomListBox	
Left	published		TControl	
MultiSelect	published	False	TCustomListBox	
Name	published		TComponent	
ParentColor	published	False	TControl	
ParentCtrl3D	published	True	TWinControl	
ParentFont	published	True	TControl	
ParentShowHint	published	True	TControl	
PopupMenu	published		TControl	
ShowHint	published	False	TControl	
TabOrder	published		TWinControl	
TabStop	published	False	TWinControl	
TabWidth	published	0	TCustomListBox	
Tag	published	0	TComponent	
Top	published		TControl	
UseFilter	published	False	TRuleView	
Visible	published	True	TControl	
Width	published		TControl	

ExpertSystem: TExpertSystem
Sistema especialista cujas regras serão exibidas;

HeadFilter: TSingleIndexTree
Caso não se deseje exibir todas as regras, é possível selecionar somente aquelas que apresentem uma determinada variável em suas conclusões (como ocorre na opção Visualizar do shell Expert SINTA). Para isso, acrescente a esta árvore o(s) código(s) das variáveis que irão corresponder às regras desejadas;

UseFilter: boolean
Para que a seleção de regras feita com HeadFilter seja efetuada, esta propriedade deve ter o valor true.

Métodos

Método	Visibilidade	Documentado em	
FillRules	public	TRuleView	
SetHorizontalBar	public	TRuleView	
Demais métodos	public/protected	TCustomListBox	

procedure FillRules
Força a exibição das regras na TRuleView (é automática quando se muda a propriedade TExpertSystem);

procedure SetHorizontalBar
Força a exibição de uma barra de rolagem horizontal (não é padrão do Windows);

Demais métodos
Consulte a documentação do Delphi/C++ Builder

Eventos

Eventos	Visibilidade	Documentado em	
OnClick	published	TControl	
OnDblClick	published	TControl	
OnDragDrop	published	TControl	
OnDragOver	published	TControl	
OnEndDrag	published	TControl	
OnEnter	published	TWinControl	
OnExit	published	TWinControl	
OnKeyDown	published	TWinControl	
OnKeyPress	published	TWinControl	
OnKeyUp	published	TWinControl	
OnMouseDown	published	TControl	
OnMouseMove	published	TControl	
OnMouseUp	published	TControl	
OnStartDrag	published	TControl	


TValuesGrid
Unidade: ExCtrls
TObject  TPersistent  TComponent  TControl  TWinControl  TCustomControl  TCustomGrid  TDrawGrid  TStringGrid  TValuesGrid

Descrição
	
Mostra no formato de um grid todos os valores de uma variável, incluindo os fatores de confiança. Pode ser usado como alternativa ao TWatchPanel.

Propriedades

Propriedade	Visibilidade	Default	Documentada em	
Align	published	alNone	TControl	
AutomaticUpdate	published	False	TValuesGrid	
BorderStyle	published	bsSingle	TCustomListBox	
Cells	public		TStringGrid	
Col	public		TCustomGrid	
ColCount	published	5	TCustomGrid	
Color	published	clWindow	TWinControl	
Cols	public		TStringGrid	
ColWidths	public		TCustomGrid	
Ctrl3D	published	True	TWinControl	
Cursor	published	crDefault	TControl	
DefaultColWidth	published	64	TCustomGrid	
DefaultDrawing	published	True	TCustomGrid	
DefaultRowHeight	published	24	TCustomGrid	
DragCursor	published	crDrag	TControl	
DragMode	published	dmManual	TControl	
Enabled	published	True	TControl	
ExpertSystem	published		TValuesGrid	
FixedColor	published	clBtnFace	TCustomGrid	
FixedCols	published	1	TCustomGrid	
FixedRows	published	1	TCustomGrid	
Font	published		TControl	
GridLineWidth	published	1	TCustomGrid	
Height	published		TControl	
HelpContext	published	0	TWinControl	
Hint	published		TControl	
Left	published		TControl	
Name	published		TComponent	
Options	published	[goFixedVertLine, goFixedHorzLine, goVertLine, goHorzLine, goRangeSelect]	TCustomGrid	
ParentColor	published	False	TControl	
ParentCtrl3D	published	True	TWinControl	
ParentFont	published	True	TControl	
ParentShowHint	published	True	TControl	
PopupMenu	published		TControl	
Row	public		TCustomGrid	
RowCount	published		TCustomGrid	
RowHeights	public		TCustomGrid	
Rows	public		TStringGrid	
ScrollBars	published	ssBoth	TCustomGrid	
Selection	public		TCustomGrid	
ShowHint	published	False	TControl	
TabOrder	published		TWinControl	
TabStop	published	False	TWinControl	
TabWidth	published	0	TCustomListBox	
Tag	published	0	TComponent	
Top	published		TControl	
VarCode	published	0	TValuesGrid	
Visible	published	True	TControl	
Width	published		TControl	

AutomaticUpdate: boolean
Quando verdadeira, o grid atualiza automaticamente a exibição das instâncias de sua variável quando necessário;

ExpertSystem: TExpertSystem
Sistema especialista cujos valores de determinada variável serão exibidos;

VarCode: integer
Variável que irá fornecer os valores para a construção das opções;

Métodos

Método	Visibilidade	Documentado em	
RefreshValues	public	TValuesGrid	
Demais métodos	public/protected	TScrollBox	

procedure RefreshValues
Atualiza os valores exibidos no grid. Não é necessário chamar caso a propriedade AutomaticUpdate seja verdadeira;

Demais métodos
Consulte a documentação do Delphi/C++ Builder;

Eventos

Eventos	Visibilidade	Documentado em	
OnClick	published	TControl	
OnColumnMoved	published	TDrawGrid	
OnDblClick	published	TControl	
OnDragDrop	published	TControl	
OnDragOver	published	TControl	
OnDrawCell	published	TDrawGrid	
OnEndDrag	published	TControl	
OnEnter	published	TWinControl	
OnExit	published	TWinControl	
OnGetEditMask	published	TDrawGrid	
OnGetEditText	published	TDrawGrid	
OnKeyDown	published	TWinControl	
OnKeyPress	published	TWinControl	
OnKeyUp	published	TWinControl	
OnMouseDown	published	TControl	
OnMouseUp	published	TControl	
OnRowMoved	published	TDrawGrid	
OnSelectCell	published	TDrawGrid	
OnSetEditText	published	TDrawGrid	
OnStartDrag	published	TControl	
OnTopLeftChanged	published	TDrawGrid	


TWatchPanel
Unidade: ExCtrls
TObject  TPersistent  TComponent  TControl  TWinControl  TCustomControl  TCustomPanel  TWatchPanel

Descrição
	
Este componente consiste de dois painéis, exibindo variáveis no painel superior e valores no inferior. Tais valores são atualizados automaticamente durante uma consulta ao sistema especialista associado.

Propriedades

Propriedade	Visibilidade	Default	Documentada em	
AddAll	published	True	TWatchPanel	
Align	published	alNone	TControl	
BevelInner	published	bvNone	TCustomPanel	
BevelOuter	published	bvRaised	TCustomPanel	
BevelWidth	published	1	TCustomPanel	
BorderStyle	published	bsSingle	TCustomListBox	
BorderWidth	published	0	TCustomListBox	
Color	published	clWindow	TWinControl	
Ctrl3D	published	True	TWinControl	
Cursor	published	crDefault	TControl	
DragCursor	published	crDrag	TControl	
DragMode	published	dmManual	TControl	
Enabled	published	True	TControl	
ExpertSystem	published		TWatchPanel	
Font	published		TControl	
Height	published		TControl	
HelpContext	published	0	TWinControl	
Hint	published		TControl	
labelVars	public		TWatchPanel	
labelValues	public		TWatchPanel	
Left	published		TControl	
listVars	public		TWatchPanel	
listValues	public		TWatchPanel	
Locked	published	False	TCustomPanel	
Name	published		TComponent	
ParentColor	published	False	TControl	
ParentCtrl3D	published	True	TWinControl	
PopupMenu	published		TControl	
ShowHint	published	False	TControl	
TabOrder	published		TWinControl	
TabStop	published	False	TWinControl	
Tag	published	0	TComponent	
Top	published		TControl	
ValuesListHeight	published	50	TWatchPanel	
Visible	published	True	TControl	
Width	published		TControl	

AddAll: boolean
Se verdadeira, o painel mostrará todas as variáveis automaticamente ao indicarmos o sistema especialista (TExpertSystem) correspondente;

ExpertSystem: TExpertSystem
Associa um sistema especialista ao objeto em questão;

labelVars: TLabel
listVars: TListBox
labelValues: TLabel
listValues: TListBox
Componentes internos ao painel Watch;

ValuesListHeight: integer
Define a altura da lista de valores (o painel inferior). Ele permanece fixo quando o tamanho do WatchPanel muda;

Métodos

Método	Visibilidade	Documentado em	
AddNewItem	public	TWatchPanel	
Clear	public	TWatchPanel	
DeleteItem	public	TWatchPanel	
FillVars	public	TWatchPanel	
Update	public	TWatchPanel	
Demais métodos	public/protected	TCustomPanel	

procedure AddNewItem(V: integer)
Adiciona ao painel de variáveis a variável de código "V";

procedure Clear
Limpa todo o painel de variáveis;

procedure DeleteItem(V: integer; Name: string)
Delete do WatchPanel a variável de código "V". Se for especificado um nome diferente de vazio (''), será deletada a variável de nome "Name" (o código será ignorado);

procedure FillVars
Insere todas as variáveis do sistema ExpertSystem no painel;

procedure Update
Atualiza a exibição de valores da variável selecionada no painel superior. Realizada automaticamente pelo sistema ExpertSystem associado;

Demais métodos
Consulte a documentação do Delphi/C++ Builder;

Eventos

Eventos	Visibilidade	Documentado em	
OnClick	published	TControl	
OnDblClick	published	TControl	
OnDragDrop	published	TControl	
OnDragOver	published	TControl	
OnEndDrag	published	TControl	
OnEnter	published	TWinControl	
OnExit	published	TWinControl	
OnMouseDown	published	TControl	
OnMouseMove	published	TControl	
OnMouseUp	published	TControl	
OnResize	published	TCustomPanel	
OnStartDrag	published	TControl	


TWhyDialog
Unidade: ExDlgWhy
TObject  TPersistent  TComponent  TWhyDialog

Descrição
	
Caixa de diálogo que exibe uma explicação/ajuda sobre determinada pergunta;

Propriedades

Propriedade	Visibilidade	Default	Documentada em	
Name	published		TComponent	
Tag	published	0	TComponent	

Métodos

Método	Visibilidade	Documentado em	
Execute	public	TWhyDialog	
Demais métodos	public/protected	TComponent	

function Execute(V: integer; ES: TExpertSystem): boolean
Se a variável "V" tiver uma explicação associada, ela será exibida. Caso contrário, o sistema montará uma explicação de acordo com o estado atual da máquina de inferência do sistema "ES". A função retorna falso se a caixa de diálogo não for exibida;

Demais métodos
Consulte a documentação do Delphi/C++ Builder;

Eventos

Este componente não possui eventos.


Sobre o Projeto

Descrição da equipe responsável pela implementação e manutenção da Expert SINTA VCL e do shell Expert SINTA.

A Equipe
Membros da equipe de Sistemas INTeligente Aplicados da UFC.

Agradecimentos
Agradecimentos aos demais colaboradores deste trabalho.


Sobre
o Projeto 


A Equipe


O Projeto Expert SINTA foi iniciado em maio de 1995 no Laboratório de Inteligência Artificial (LIA), localizado no Departamento de Computação da Universidade Federal do Ceará (UFC). Desde então, o grupo Sistemas INTeligentes Aplicados (SINTA) vem desenvolvendo atividades relacionadas a aplicações práticas de técnicas de Inteligência Artificial. 
Com o foco principal em sistemas especialistas, criou-se uma ferramenta de uso geral para criação de bases de conhecimento, o Expert SINTA. Esta ferramenta é utilizada para criação de sistemas dentro da própria UFC, bem como para o uso em aulas sobre Inteligência Artificial. O Expert SINTA também foi apresentado com sucesso na VI Semana de Informática, promovida pela UFBA, na seção de ferramentas do Simpósio Brasileiro de Engenharia de Software/96 e em eventos internos da Universidade Federal do Ceará, resultando em comentários favoráveis de diversas regiões do Brasil. 
Para satisfazer mais necessidades profissionais, foi criado o programa Desenvolvimento Integrado de Sistemas Especialistas (DISE), envolvendo a tecnologia utilizada no Expert SINTA e ferramentas de programação de terceiros. Desta forma, procura-se preencher todos os requisitos para a construção de sistemas especialistas de bom nível, além de atuar junto à comunidade acadêmica com ferramentas didáticas. Como fruto deste trabalho, veio a Expert SINTA Visual Component Library (Expert SINTA VCL), apresentada na seção de ferramentas do Simpósio Brasileiro de Engenharia de Software/97.
Entre todos os integrantes que participam ou participaram do grupo, temos: 

•	João Fernando Lima Alcântara foi programador auxiliar, além de colaborar com conselhos e modificações da ferramenta. Também desenvolveu projetos de sistemas especialistas com o uso do Expert SINTA; 
	
•	José Helano Matos Nogueira, orientador do grupo original e grande divulgador do nosso trabalho em diversas outras entidades de ensino superior. Atualmente não mais participa diretamente do grupo; 
	
•	Rafael Castro de Andrade, estudante da Universidade Estadual do Ceará (UECE), principal responsável por testes, além de várias outras críticas. Também atuou como desenvolvedor de sistemas especialistas em conjunto com o grupo; 
	
•	Ricardo Bezerra de Andrade e Silva, programador-chefe do Expert SINTA e junto a Ricardo Silvestre projetou as características básicas da ferramenta. É o responsável pela manutenção do programa, bem como pela implementação da Expert SINTA Visual Component Library; 
	
•	Ricardo Sousa Silvestre, criador da idéia original do shell Expert SINTA, sendo um dos programadores e projetistas da primeira versão da ferramenta; 
	
•	Shirlene Correia de Holanda, aluna da Odontologia, desenvolveu um projeto de sistema especialista para patologias bucais junto ao grupo; 
	
•	Tarcísio Cavalcante Pequeno, criador do grupo e principal responsável;


Sobre
o Projeto 

Agradecimentos

O grupo gostaria de agradecer às seguintes pessoas:

Financiamento

	CAPES
	CNPq

A equipe de desenvolvimento do SECAJU

	Quélzia Maria Silva Melo  (Doutora em Entomologia)
	Antonio Lindemberg M. Mesquita (Mestre em Entomologia)
	Lianna Maria Saraiva Teixeira (Mestre em Fitossanidade)
	Francisco das Chagas O. Freire (Doutor em Fitopatologia).

Demais colaboradores

	Genaro de Azevedo França
	Marcelo Antonio Thomaz de Aragão
	Marcelino Cavalcante Pequeno

e a todos os usuários do Expert SINTA.


Expert SINTA Visual Component Library
© 1995-98 Laboratório de Inteligência Artificial - LIA
http://www.lia.ufc.br
