# Supplementary material for: Implementation of expert systems to support the functional evaluation of stand-to-sit activity
Source: Biomed Eng Online. 2014 Jul 21;13:98. doi: 10.1186/1475-925X-13-98 (PMC4120714; doi:10.1186/1475-925X-13-98)
Supplement: Additional file 2 — Shell Expert Sinta. [file 1475-925X-13-98-S2.zip › Leia-me.doc]

Expert SINTA versão 1.1b

Esse documento contém informações adicionais sobre o pacote incluindo a presente versão do Expert SINTA, documentação e exemplos.

1 Instalação

Para utilizar a presente versão do Expert SINTA, simplesmente copie os arquivos desse pacote em uma pasta da sua preferência (como C:\EXSINTA). O pacote consiste dos seguinte arquivos:

- LEIA-ME.DOC (o presente documento);
- EXSINTA.EXE (Expert SINTA, o programa principal. A versão incluída é para Windows 95/Windows NT ou superior);
- EXSINTA.HLP (ajuda do Expert SINTA);
- EXSINTA.CNT (conteúdo da ajuda do Expert SINTA, utilizado indiretamente pelo EXSINTA.HLP);
- EXSINTA.TIP (dicas sobre o uso do Expert SINTA)
- SECAJU.BCM (exemplo de sistema especialista desenvolvido com o Expert SINTA);
- SECAJU.HLP (ajuda do SECAJU);

2 Sobre o Expert SINTA

O *software* foi desenvolvido por bolsistas da CAPES e CNPq ligados ao Laboratório de Inteligência Artificial (LIA), unidade localizada na Universidade Federal do Ceará. Os responsáveis pelo programa formam o grupo Sistemas INTeligentes Aplicados (SINTA). Maiores informações na ajuda on-line (EXSINTA.HLP)

3 Histórico

- novembro de 95: primeiro protótipo (beta 1) em Visual BASIC. Incluía opções básicas de consulta e criação de bases. Utilizava arquivos Microsoft Access para armazenar as bases de conhecimento. MUUUUUITO lento devido ao arquivo Access;
- março de 96: versão beta 2, com alterações radicais. Adaptação para a Borland Delphi, uso de um formato próprio de arquivo para armazenar as bases de conhecimento, implementação completa do editor de bases e depurador. Desempenho bem superior;
- julho de 96: versão beta 3, correção de (muitos) bugs, refinamentos para melhoria de performance, elaboração da ajuda on-line, inclusão de opções de configuração, vários aperfeiçoamentos de interface;
- setembro de 96: versão 1.0, correção de bugs no editor de variáveis e outros menores, finalização da implementação de variáveis numéricas, inclusão de um botão para voltar à pergunta anterior, finalização da ajuda on-line, modificações no botão "Por que?", pequenas mudanças na interface;
- setembro de 96: versão 1.0a, correção de um bug em relação às variáveis, evitando a falha de reconhecimento de uma variável se existirem algumas com letras maiúsculas e outras com minúsculas. Agora também foi permitida a mudança do nome de uma variável de maiúsculas para minúsculas. O cálculo do fator de confiança na conjunção (E) foi alterado;
- 07 de outubro de 96: versão 1.0b, correção de um bug sério na máquina de inferência, que ficava imprevisível quando um regra chamava outra antes de ser concluída. Dois pequenos bugs foram retirados da opção de criar uma nova regra a partir de um modelo: quando alguma regra era mudada de posição, um novo modelo criado nem sempre correspondia à regra escolhida. E, quando existiam variáveis numéricas em uma regra que se usou como modelo, as fórmulas matemáticas que eram comparadas ou atribuídas a uma variável numérica ficavam "ligadas" (as mudanças na regras nova mudavam também a regra de modelo). Esses erros foram retirados;
- 21 de outubro de 1996: versão 1.1. Correções de pequenos bugs, inclusão de várias outras características, como mudar (finalmente!) a ordem na qual os valores aparecem durante as perguntas e resultados, personalização dos cálculos dos fatores de confiança, facilidades para simular encadeamento para frente e a possibilidade de permitir ou não a entrada de graus de confiança em uma pergunta. Obviamente, os arquivos criados com o Expert SINTA 1.1 não podem ser abertos no ExSINTA 1.0x;
- 11 de março de 1997: versão 1.1a. Pequenos bugs retirados, melhorias na interface (principalmente mudanças no editor de regras), acréscimo de nova ferramenta para depuração (analisador de dependências). O formato do arquivo é o mesmo da versão 1.1.
- 27 de agosto de 1997: versão 1.1b. Algumas alterações na interface, reorganização do código fonte para adaptar-se à nova biblioteca Expert SINTA VCL, um conjunto de componentes distríbuídos em separado para a programação de sistemas especialistas, com a qual o próprio Expert SINTA foi implementado. A versão de manutenção mais atual data de 02 de maio de 98.

4 Licença de uso

O Expert SINTA 1.1b é um *freeware*. SINTA-se à vontade em distribuir o programa entre os amigos e familiares :-). No entanto, os autores se reservam o direito de exigir notificações dos interessados em distribuição do *software* em massa, através de meios como CD-ROM, disponibilização em BBS e sites Internet. O programa deve ser sempre distribuído com todos os arquivos que formam este pacote (ver seção 1).

Não é autorizado o uso do sistema SECAJU em nenhuma hipótese, a não ser com o intuito de demonstração do Expert SINTA ou para autoaprendizado.

5 Extensões do Expert SINTA

- Para maior flexibilidade na criação de sistemas especialistas, não deixe de ver a Expert SINTA Visual Component Library. Esta é uma biblioteca de componentes que permite a criação de *front-ends* para as bases de conhecimento geradas com o Expert SINTA valendo-se de uma linguagem de programação para ambiente Windows, como o Borland Delphi e o Borland C++ Builder. Se você é um programador e está interessado, consulte a homepage do projeto para maiores informações;
- O Expert SINTA também possui um *runtime*, um programa criado especialmente para a execução de consultas às bases de conhecimento sem opções de edição. Desta forma, o usuário final não se confunde com menus e botões a mais e há menos custos de distribuição, pois o *runtime* é menor que o Expert SINTA completo. Consulte a homepage abaixo;

Críticas e sugestões podem (e devem) ser enviadas para o e-mail bezerra@lia.fc.br

A página WWW do Expert SINTA é http://www.lia.ufc.br/~bezerra/exsinta/

OS AUTORES NÃO SE RESPONSABILIZAM POR NENHUM DANO OCASIONAL CAUSADO PELO USO DO SOFTWARE ACIMA CITADO. O PRODUTO É FORNECIDO "COMO ESTÁ" (*AS IS*), OU SEJA, NENHUM SUPORTE TÉCNICO É GARANTIDO. É VETADO O USO DO SOFTWARE PARA USO COMERCIAL, A NÃO SER ATRAVÉS DE NEGOCIAÇÕES COM OS AUTORES.

Expert SINTA © 1995-1998 / Laboratório de Inteligência Artificial LIA/UFC.

**http://www.lia.ufc.br**
