# Supplementary material for: Implementation of expert systems to support the functional evaluation of stand-to-sit activity
Source: Biomed Eng Online. 2014 Jul 21;13:98. doi: 10.1186/1475-925X-13-98 (PMC4120714; doi:10.1186/1475-925X-13-98)
Supplement: Additional file 2 — Shell Expert Sinta. [file 1475-925X-13-98-S2.zip › manual(1).zip!/manual.doc]

LIA
Laboratório de Inteligência Artificial
http://www.lia.ufc.br


Expert SINTA versão 1.1
Uma ferramenta visual para criação de sistemas especialistas

manual do usuário

Índice


Bem-Vindo ao Expert SINTA

O que são sistemas especialistas e o que o Expert SINTA é capaz de fazer por você.	


	Sistemas especialistas são programas de computador que procuram atingir soluções de determinados problemas do mesmo modo que especialistas humanos, se estiverem sob as mesmas condições. Apesar das limitações das máquinas, é possível, hoje, a construção de sistemas especialistas com alto grau de desempenho, dependendo da complexidade de sua estrutura e do grau de abrangência desejado.

	A arquitetura mais comum de sistemas especialistas é a que envolve regras de produção (production rules). Essas regras são simplesmente um conjunto de condições no estilo SE... ENTÃO..., com a possibilidade de inclusão de conectivos lógicos relacionando os atributos no escopo do conhecimento e o uso de probabilidades, como vemos no exemplo a seguir:

	SE 	carne = vermelha
	E   	cor preferida = tinto
	OU	temperatura = ambiente
	ENTÃO
	    	melhor vinho = exemplo CNF 70;

	Um sistema de bom tamanho tem em torno de centena de regras (considerando aqueles que utilizam regras). Visando uma maior viabilidade econômica na implementação de um sistema especialista, e considerando-se que diversos sistemas compartilham uma máquina de inferência e outras características comuns de ambiente, foram criadas ferramentas, shells, aptas a realizar muito do trabalho necessário para transpor um sistema especialista para um computador. Essas ferramentas permitem que o criador do sistema preocupe-se somente com a representação do conhecimento do especialistas, deixando para a shell a tarefa de interpretar o conhecimento representado e executá-lo em uma máquina, além de permitir depurações e explicações de como o computador chegou àquela(s) conclusão(ões). A principal função de uma shell é simplificar ao máximo o trabalho de implementação de um sistema especialista e permitir seu uso por qualquer pessoa sem conhecimentos de informática.

	O Expert SINTA é uma ferramenta computacional que utiliza técnicas de Inteligência Artificial para geração automática de sistemas especialistas. Esta ferramenta utiliza um modelo de representação do conhecimento baseado em regras de produção e probabilidades, tendo como objetivo principal simplificar o trabalho de implementação de sistemas especialistas através do uso de uma máquina de inferência compartilhada, da construção automática de telas e menus, do tratamento probabilístico das regras de produção e da utilização de explicações sensíveis ao contexto da base de conhecimento modelada. Um sistema especialista baseado em tal tipo de modelo é bastante útil em problemas de classificação. O usuário responde a uma sequência de menus, e o sistema encarrega-se-á de fornecer respostas que se encaixem no quadro apontado pelo usuário. Como exemplos, temos sistemas de diagnósticos médicos e configuração de redes de computadores.

	Entre outras características inerentes ao Expert SINTA, temos: 

		utilização do encadeamento para trás (backward chaining);
		utilização de fatores de confiança;
		ferramentas de depuração;
		possibilidade de incluir ajudas on-line para cada base.

	O Expert SINTA procura ser uma ferramenta de criação geral. Dúvidas e sugestões podem ser enviadas ao serviço de suporte:


Laboratório de Inteligência Artificial - LIA


E-mails:	bezerra@lia.ufc.br
	jnando@lia.ufc.br
	helano@lia.ufc.br
	adm@lia.ufc.br


Universidade Federal do Ceará - UFC

Capítulo I - Planejando um sistema especialista

Como se deve planejar um sistema especialista no Expert SINTA, algumas definições importantes da área e como entender o funcionamento de um sistema construído.
	Esse capítulo procura trazer informações básicas sobre a idéia por trás dos sistemas especialistas e alguns conceitos da área de Inteligência Artificial. Também é explicado o funcionamento interno do Expert SINTA, o modo como representa o conhecimento e o uso de probabilidades na sua máquina de inferência. Aqueles que já estão familiarizados aos assuntos abordados a seguir podem examinar rapidamente o seu conteúdo. Os iniciantes deverão dispensar um pouco mais de tempo.	Esse capítulo procura trazer informações básicas sobre a idéia por trás dos sistemas especialistas e alguns conceitos da área de Inteligência Artificial. Também é explicado o funcionamento interno do Expert SINTA, o modo como representa o conhecimento e o uso de probabilidades na sua máquina de inferência. Aqueles que já estão familiarizados aos assuntos abordados a seguir podem examinar rapidamente o seu conteúdo. Os iniciantes deverão dispensar um pouco mais de tempo.


1.1 Arquitetura de um sistema especialista no Expert SINTA

	Os sistemas especialistas (SE) gerados no Expert SINTA seguem a arquitetura abaixo:


Figura 1.1 - Arquitetura simplificada do 
Expert SINTA

onde:

		base de conhecimentos representa a informação (fatos e regras) que um especialista utiliza, representada computacionalmente; 

		editor de bases é o meio pelo qual a shell permite a implementação das bases desejadas; 

		máquina de inferência é a parte do SE responsável pelas deduções sobre a base de conhecimentos; 

		banco de dados global são as evidências apontadas pelo usuário do sistema especialista durante uma consulta.

	O objetivo do Expert SINTA é simplificar ao máximo as etapas de criação de um SE completo. Para tanto, já oferece uma máquina de inferência básica, fundamentada no encadeamento para trás (backward chaining).

	O encadeamento para trás destaca-se em problemas nos quais há um grande número de conclusões que podem ser atingidas, mas o número de meios pelos quais elas podem ser alcançadas não é grande (um sistema de regras de alto grau de fan out), e em problemas nos quais não se pode reunir um número aceitável de fatos antes de iniciar-se a busca por respostas. O encadeamento para trás também é mais intuitivo para o desenvolvedor, pois é fundamentada na recursão, um meio elegante e racional de programação, para onde a própria Programação em Lógica se direcionou. Em nenhum momento, porém, deixa-se de reconhecer que o encadeamento para frente (foreward chaining) possui vantagens em determinadas ocasiões. É possível, entretanto, simular o encadeamento para frente, como será visto no capítulo II, Implementando o sistema.

1.2 Utilizando regras de produção

1.2.1 Visão geral

	As regras de produção são populares por possuírem as seguintes vantagens:

		Modularidade: cada regra, por si mesma, pode ser  considerada como uma peça de  conhecimento independente;

		Facilidade de edição (uma conseqüência da modularidade): novas regras podem ser acrescentadas e antigas podem ser modificadas com relativa independência;

		Transparência do sistema: garante maior legibilidade da base de conhecimentos.

	Portanto, é preciso ter em mente que a modularidade de um sistema baseado nessa arquitetura permite a construção passo-a-passo da base de conhecimentos, ou seja, é possível realizar vários testes com apenas um subconjunto de regras concluído. Obviamente, sabe-se que menos regras implicam geralmente em um menor número de casos abrangidos. 

1.2.2 As regras no Expert SINTA

	Vejamos o seguinte exemplo (extraído da base SECAJU, um exemplo que acompanha o Expert SINTA):


premissasdaregrapremissasdaregra
	SE		galerias nos ramos ou inflorescências
	OU		inflorescências = murchas ou secas
conclusõesconclusões	E		brotações novas murchas
	E		orifícios laterais nos ramos ou inflorescências
	ENTÃO	praga = broca das pontas [90%]


 Figura 1.2 - Regra de Produção

	Denomina-se os conseqüentes de uma regra como as cabeças da regra e os antecedentes, caudas. Esta notação é proveniente da linguagem PROLOG, na qual as conclusões encabeçam as cláusulas.

	Para o projetista do conhecimento que cria bases utilizando o Expert SINTA, o seguinte critério para definições de assertivas deve ser seguido:

( I ). A estrutura de cada cauda (premissa) deve obedecer ao seguinte modelo: 

<conectivo>	<atributo>	<operador>	<valor>

			
onde:

		conectivo é um dos seguintes elementos utilizados na lógica clássica: NÃO, E, OU. Sua função é unir a sentença ao conjunto de premissas que formam a seção de antecedentes de uma regra.

		atributo é uma variável capaz de assumir uma ou múltiplas instanciações no decorrer da consulta à base de conhecimentos. Cabe ao desenvolvedor definir o tipo de atributo. Um atributo é uma entidade totalmente abstrata, capaz de armazenar listas de valores cujo significado depende do contexto da base. Por exemplo, um atributo doença, no SECAJU, é capaz de ser instanciado a qualquer um ou vários elementos da lista de valores pré-definida [mofo preto, oidio, antracnose]. Números também podem ser atribuídos a variáveis;

		operador é um elo de ligação entre o atributo e o valor da premissa que define o tipo de comparação a ser realizada. São operadores relacionais: =, >, <=, <>, entre outros;

		valor é um item de uma lista a qual foi previamente criada e relacionada a um atributo. Como veremos posteriormente, a interface visual do Expert SINTA foi idealizada de modo a minimizar o trabalho do responsável pela implementação da base de conhecimentos. Para tanto, o Expert SINTA exige que os atributos sejam definidos antes de criarmos uma regra que o utilize. No momento da criação de um atributo, também é possível que seja definida uma lista de valores que constituirá o universo ao qual suas instanciações devem necessariamente pertencer. As listas criadas podem ser utilizadas para construção de menus, caso o sistema especialista necessite efetuar uma pergunta ao usuário referente àquele determinado atributo. Um valor também pode ser um número, caso o atributo o permita.

 ( II ). A estrutura de cada cabeça (conclusão) deve obedecer ao seguinte modelo:

<atributo>	=	<valor>	<grau de confiança>


onde:

		atributo eqüivale ao mesmo atributo usado em caudas;

		“=” é um operador de atribuição e não de igualdade. Ou seja, o atributo, nas cabeças de regra, é sempre instanciado a um valor. Dependendo se a variável pode ou não acumular múltiplas instanciações, o novo valor substituíra o antigo ou será empilhado com os demais.

		valor eqüivale ao mesmo valor utilizado em caudas;

		grau de confiança é uma porcentagem indicando a confiabilidade daquela conclusão específica da regra. O grau de confiança varia de 0% a 100%.

	Veja a seção 1.4 para maiores detalhes sobre graus de confiança.

1.3 Acompanhando a execução de um sistema

1.3.1 Conhecimento procedural x conhecimento declarativo

	Quando uma pessoa tem conhecimento de algum fato, certamente ela poderá extrair tudo o que souber sobre aquele fato quando bem entender. Porém, na Inteligência Artificial, existe um problema a mais quanto ao uso do conhecimento. 

	Supõe-se que o conhecimento por si só já é o suficiente para a resolução de problemas. Essa é a idéia por trás do conhecimento declarativo: não há preocupações quanto ao seu uso, somente quanto à sua posse e especificação, e ela já garantirá o alcance dos objetivos desejados. 

	Porém, a realidade é bem mais problemática. Um computador não é capaz de decidir qual a próxima informação que ele utilizará para o desenvolvimento de uma atividade. Faltam às máquinas um modo menos metódico e linear de ação. É necessário que especifiquemos uma estratégia de uso do seu “saber”. Mas, qual é o problema, quando a máquina possui o potencial necessário? Vejamos um exemplo:

	Todos nós sabemos calcular o fatorial de um número. O fatorial de zero é um, e o fatorial dos demais números positivos é simplesmente ele multiplicado pelo fatorial do seu antecessor (como quando dizemos que o fatorial de 5 é 5 vezes o fatorial de 4). Ao colocarmos essa representação no computador, podemos representar simbolicamente por

	Fatorial (n) = n x Fatorial(n - 1)
	Ah, e é claro: Fatorial(0) = 1

	O conhecimento está aí: agora devemos ditar ao computador o meio pelo qual ele utilizará tais afirmativas. Uma estratégia bastante comum é simplesmente percorrer as informações na ordem: inicialmente, a primeira; depois, se necessário a seguinte, e assim vai. Mas, se desejarmos calcular o prosaico fatorial de 2? Para a máquina, fatorial(2) = 2 x fatorial(1). E quanto é o fatorial de 1? Nesse caso, devemos voltar à primeira informação de como se calcula o fatorial, Fatorial (n) = n x Fatorial(n - 1). Assim chegamos que fatorial(1) é 1 vezes fatorial de zero. E o fatorial de zero? Observe que, como temos um mecanismo fixo de extração de dados, não podemos avaliar a segunda informação enquanto não terminarmos a primeira. Assim, simplesmente o computador calcularia que o fatorial de zero é zero vezes o fatorial de menos um! E continuaria eternamente com essa regra.

	Esse tipo de conhecimento que depende de uma regra de extração chamamos de conhecimento procedural. Nota-se que, se simplesmente invertêssemos a ordem das informações, chegaríamos a um resultado (sempre antes de calcular um fatorial de um número, a máquina verificaria se esse número é zero)! Por isso, o uso do conhecimento é uma questão maior que simplesmente uma descrição do saber.

1.3.2 O método de extração procedural de conhecimento do Expert SINTA

	O Expert SINTA utiliza o encadeamento para trás, o modo mais comum de utilização de um sistema especialista. O projetista deve incluir na definição da base quais os atributos que devem ser encontrados (ou seja, os objetivos - goals - do sistema especialista). A máquina de inferência encarrega-se de encontrar uma atribuição para o atributo desejado nas conclusões das regras (após o ENTÃO...). Obviamente, para que a regra seja aprovada, suas premissas devem ser satisfeitas, obrigando à máquina a encontrar os atributos das premissas para que possam ser julgadas, acionando um encadeamento recursivo. Caso o atributo procurado não seja encontrado em nenhuma conclusão de regra, uma pergunta direta é feita ao usuário.

1.3.3 Exemplo de encadeamento para trás

Sejam as seguintes regras um sistema especialista para “decidir se devo ou não ir à praia amanhã”.

REGRA 1
SE  	amanhã pode chover = Não
E	tenho dinheiro suficiente = Sim
E	tenho tempo suficiente = Sim
ENTÃO devo ir à praia = Sim

REGRA 2
SE  	amanhã pode chover = Sim
OU	tenho dinheiro suficiente = Não
OU	tenho tempo suficiente = Não
ENTÃO devo ir à praia = Não

REGRA 3
SE	o serviço de meteorologia disse que vai chover amanhã = Sim
ENTÃO	amanhã pode chover = Não

REGRA 4
SE	não vou sair hoje = Sim
E	nenhuma emergência ocorrer = Sim
ENTÃO	tenho dinheiro suficiente = Sim

REGRA 5
SE	minha namorada ligar = Sim
ENTÃO	não vou sair hoje = Não

REGRA 6
SE	meu orientador passar trabalho extra = Sim
ENTÃO	tenho tempo suficiente = Não

	Obs.: Note que as regras acima já estão na sintaxe de regras aceita pelo Expert SINTA, o que demonstra a sua facilidade de entendimento. Os valores atribuídos não precisam ser somente Sim e Não. Veja o capítulo II, Implementando o sistema, para maiores explicações.

	Examinando o pequeno sistema especialista acima, claramente distinguimos que o seu objetivo é determinar o valor da variável devo ir à praia. Então, iniciamos a busca por ela. O Expert SINTA procura as regras nas quais a variável sendo procurada no momento pode receber um valor se a regra for aceita (ou seja, quando ela aparece após o ENTÃO). Em seguida, a máquina de inferência verifica se a regra vale ou não. No exemplo dado, o sistema avalia a regra 1 para poder determinar se devo ir à praia ou não. Mas, para isso, temos que determinar se todas as premissas (amanhã pode chover = Não, tenho dinheiro suficiente = Sim, tenho tempo suficiente = Sim) são verdadeiras. Para descobrir se amanhã pode chover, tenho que repetir o processo, avaliando a regra 3. Agora tenho que saber se a meteorologia disse sobre a possibilidade de chuva amanhã. Como não existe nenhuma regra que possa concluir o que a meteorologia disse, o Expert SINTA realiza uma pergunta ao usuário, do tipo “A meteorologia afirmou se amanhã pode chover (Sim/Não)?” (claro, o computador não irá criar uma frase, essa é uma das tarefas do criador da base de conhecimento - veja o capítulo II, Implementando o sistema, para maiores detalhes).

	Se a meteorologia disser que amanhã vai chover, então primeira premissa da regra 1 passará no teste (quem confia nos serviços meteorológicos?). Avaliando a segunda premissa e repetindo o processo do encadeamento para trás, chegamos à regra 4. Mas, para descobrir se eu vou sair hoje ou não, é preciso recorrer à regra 5 (observa-se a forte idéia de recursão no backward chaining). A regra 5 leva a uma pergunta, pela qual concluiremos se tal regra vai ser aprovada. Se realmente for aprovada, a primeira premissa da regra 4 é verdadeira, fazendo com que verifiquemos agora a segunda premissa. Quando todas as premissas da primeira regra forem avaliadas, podemos determinar se a regra 1 foi aprovada ou não. Caso contrário, passamos para a próxima regra que possa concluir um valor para a variável devo ir à praia.
1.3.4 Variáveis univaloradas x variáveis multivaloradas

	Outra questão deve ser levada em conta durante o acompanhamento da execução de um sistema especialista. Uma única variável pode receber vários valores em uma única consulta ao sistema. É muito comum, por exemplo, em sistemas de diagnóstico médico, onde o paciente pode apresentar mais de uma doença. Portanto, é importante saber lidar com variáveis que podem ter apenas uma instanciação (univalorada) ou múltiplas (multivaloradas). Para saber como indicar ao Expert SINTA quais são as variáveis multivaloradas e univaloradas, consulte o capítulo II, Implementando o sistema.

	Quando a máquina de inferência está atrás de encontrar instanciações para uma variável univalorada, ela irá procurar até encontrar um valor ou até esgotar todas as possibilidades da base de conhecimento. Se, por algum motivo, durante a busca de uma outra variável, uma variável univalorada receber um valor quando já possuía outro, esse valor antigo será descartado e o novo vigorará.

	A busca de valores para variáveis multivaloradas prossegue até que toda a base de conhecimento seja explorada. Os valores permanecem acumulados. É nessa hora que é preciso ter cuidado com contradições presentes na base. O Expert SINTA, na presente versão, não faz verificações de inconsistências lógicas.

Obs.: variáveis numéricas são tratadas como univaloradas, sempre.

1.3.5 Leitura adicional: conhecimento monotônico x conhecimento não-monotônico

	Digamos que você estacionou o seu carro em frente ao local de trabalho. Evidentemente, ao retornar, você saberá o local onde deixou o seu carro e saberá que o utilizará para voltar para casa. Mas, em um intervalo de trabalho, alguém lhe diz que seu carro foi roubado! Agora você sabe que não o encontrará onde o tinha deixado.

	No dia-a-dia, enfrentamos situações que modificam as nossas certezas. O que antes tínhamos por certo agora estamos convictos que não são mais realidade. Isso porque nós pensamos em um tipo de lógica não-monotônica, ou seja, podemos receber informações que contrarie as que já possuímos.

	No tratamento tradicional de informações da Inteligência Artificial, o conhecimento monotônico, ou seja, aquele que ao acrescentarmos novas informações nunca fica contraditório, inconsistente, é o comumente usado. O Expert SINTA trata o conhecimento de forma essencialmente monotônica.

1.4 Fatores de confiança

1.4.1 Visão geral

	No exemplo dado na seção 1.3.3, as variáveis recebiam apenas valores determinísticos (“sim” e “não”). Porém, é comum que não queiramos determinar com exatidão o valor que cada variável pode receber.

	Sabemos que o conhecimento humano não é determinístico. Não há especialista que sempre se encontre em condições de afirmar determinada conclusão com certeza absoluta. Graus de confiança são freqüentemente atribuídos às suas respostas,  principalmente quando existe mais de uma. Este, sem dúvida, é um dos mais fortes pontos críticos na elaboração de uma representação computacional do saber humano. 

	Vejamos a dificuldade em representar a confiabilidade das informações:

		Especialistas humanos não se sentem confortáveis em pensar em termos de probabilidade. Suas estimativas não precisam corresponder àquelas definidas matematicamente;

		Tratamentos rigorosamente matemáticos de probabilidade utilizam informações nem sempre disponíveis ou simplificações que não são claramente justificáveis em aplicações práticas.

	Na verdade, existem duas correntes de pensamento: aquela que utiliza fórmulas estatísticas rigorosas, com teoria das probabilidades, e aquela que utiliza uma abordagem da teoria das possibilidades sobre os fatores de certeza, ou seja, mais generalizada e sem uma base matemática forte. O Expert SINTA utiliza atualmente uma abordagem possibilista.


1.4.2 O cálculo de probabilidades no Expert SINTA

	Vejamos como se atribuem fatores de confiança na presente versão do Expert SINTA:

	Caso 1: Quando queremos saber o valor final atribuído às variáveis na conclusão de um regra.

	Seja c1 o grau de confiança atribuído ao resultado final da premissa de uma regra r. Na conclusão de r, devemos ter expressões como var = value CNF c2, onde var é uma variável, value é um termo qualquer que pode ser atribuído a uma variável, c2 é um real pertencente ao intervalo [0; 100] que representa o grau de confiança da atribuição. Mas, c2 é apenas uma referência, pois o valor final é dependente do resultado da premissa. Assim sendo, realizar-se-á a operação var = value CNF c1. c2.

Exemplo de aplicação:

SE 		fumagina = sim
ENTÃO 	suspeita de praga = mosca branca, grau de confiança (CNF) 70%.

	Assim sendo, supondo que o grau de confiança da igualdade fumagina = sim é 80%, teremos que à variável suspeita de praga será atribuído o valor mosca branca, com o respectivo grau de confiança 0.80 * 0.70 = 0.56 = 56%.

	Caso 2: Cálculo do grau de confiança com o operador E.

	Se possuímos duas igualdades var1 = value1 e var2 = value2, com os respectivos graus de confiança c1 e c2, temos que a sentença var1 = value1 E var2 = value2 retornará como valor de confiança c1 x c2.

Exemplo de aplicação:

SE 	estados das folhas = esfarelam facilmente 
E	presença de manchas irregulares = sim...

	Se o grau de confiança da igualdade estados das folhas = esfarelam facilmente é 80% e o grau de confiança da igualdade presença de manchas irregulares = sim é 70%, temos que a conjunção das duas sentenças retornará um valor CNF de 56%, pois esse é o produto dos dois valores.

	Caso 3: Cálculo do grau de confiança com o operador OU.

	Se possuímos duas igualdades var1 = value1 e var2 = value2, com os respectivos graus de confiança c1 e c2, temos que a sentença var1 = value1 OU var2 = value2 retornará como valor de confiança c1 + c2 - c1 x c2.


Exemplo de aplicação:

SE  	besouros vermelhos = sim 
OU 	larvas marrons = sim ...	

	Se o grau de confiança da igualdade besouros vermelhos = sim é 80% e o grau de confiança da igualdade larvas marrons = sim é 70%, temos que a disjunção das duas sentenças retornará um valor CNF de 0.70 + 0.80 - 0.70 * 0.80 = 1.50 - 0.56 = 0.94 = 94%.

	Caso 4: Quando uma variável recebe duas vezes o mesmo valor em pontos diferentes da consulta.

	Em momentos diferentes de uma consulta, uma mesma variável var pode receber o mesmo valor v, sendo que até à penúltima instanciação ela possuía grau de confiança c1, e a última atribuiu um CNF c2. Sendo assim, temos que o valor final de confiança para var = v será dado pela fórmula ca + cn - ca * cn, onde ca representa o grau de confiança antes da última mudança e cn o último grau de confiança atribuído.

Exemplo de aplicação:

	A variável doença possuía valor mofo preto com grau de confiança 60%. Após a aplicação de outras regras chegou-se a uma outra atribuição doença = mofo preto, desta vez com CNF 50%.

	O cálculo se dá de maneira semelhante à aplicação da regra OU: doença terá como um dos valores mofo preto, com respectivo grau de confiança 0.60 + 0.50 - 0.60 * 0.50 = 1.10 - 0.30 = 0.80 = 80%.

	Obs.: O sistema admite 50% como valor mínimo de confiança para que uma igualdade seja considerada verdadeira, mas esse valor pode ser modificado. O intervalo de grau de confiança varia de 0 a 100.

	Obs2.: Observe que as funções para conjunção e disjunção utilizadas seguem a Teoria dos Possibilidades, não envolvendo nenhum tratamento estatístico mais aprofundado.

	Obs3.: É possível mudar as fórmulas utilizadas. Consulte o capítulo II, Implementando o sistema.

1.5 Lidando com o desconhecido

	Vamos relembrar o exemplo dado na seção 1.3.3. Se respondermos que o serviço de meteorologia disse que amanhã VAI chover, então a regra 3 irá falhar. Mas, observe que isso NÃO quer dizer que a variável amanhã pode chover irá receber o valor “Sim”. Muito pelo contrário, essa variável não vai ser instanciada, permanecendo inalterada. Portanto, a regra 2 também vai falhar, e o sistema simplesmente responderá que não conseguiu atingir nenhuma conclusão. Quando se projeta um sistema especialista, deve-se levar em conta o maior número de possibilidades possível, para evitar mensagens desagradáveis como “Nenhum valor encontrado...”

Além do uso de graus de confiança para tratamento de incertezas, e da “não-instanciação” de variáveis, o Expert SINTA permite o uso de um valor especial, passível de ser usado por todas as variáveis. É o DESCONHECIDO, que representa uma indeterminação total sobre as instanciações de uma variável. Este valor pode ser tanto incluído nas regras como dado como resposta pelo usuário (consulte o capítulo III, Realizando uma consulta com o Expert SINTA).


1.6 Sumário

	Neste capítulo, vimos:

		A arquitetura básica de um sistema especialista construído com o uso do Expert SINTA;

		Explicações sobre regras de produção e encadeamento para trás;

		Definições de conhecimento procedural, declarativo, monotônico e não-monotônico;

		O uso de regras no Expert SINTA e explicações sobre variáveis univaloradas e multivaloradas;

		Exemplo de funcionamento da máquina de inferência;

		Tratamento de fatores de confiança e do valor DESCONHECIDO.

Capítulo II - Implementando o sistema

Com o sistema já definido, deve-se agora transportá-lo para o computador utilizando o Expert SINTA.


	O Expert SINTA foi criado de modo a permitir ao próprio analista de conhecimento implementar a base desejada. O usuário do Expert SINTA não necessita do menor conhecimento de programação, apenas saber como interagir em ambientes visuais.	Uma base de conhecimento no Expert SINTA envolve os seguintes conjuntos de atributos que devem ser indicados pelo projetista da base:		Variáveis		Regras		Perguntas		Objetivos		Informações adicionais	Quando esses elementos estiverem definidos, já se torna possível utilizar o sistema especialista.	O Expert SINTA foi criado de modo a permitir ao próprio analista de conhecimento implementar a base desejada. O usuário do Expert SINTA não necessita do menor conhecimento de programação, apenas saber como interagir em ambientes visuais.	Uma base de conhecimento no Expert SINTA envolve os seguintes conjuntos de atributos que devem ser indicados pelo projetista da base:		Variáveis		Regras		Perguntas		Objetivos		Informações adicionais	Quando esses elementos estiverem definidos, já se torna possível utilizar o sistema especialista.
2.1 Gerenciando bases

	Como padrão, O Expert SINTA grava as bases de conhecimento geradas em arquivos *.BCM. Para abrir bases pré-existentes em seus discos, utilize o menu Arquivo|Abrir. Consulte a ajuda do Windows se precisar de maiores informações de como trabalhar com a caixa de diálogo Abrir Arquivo. Para criar uma base totalmente nova, use o menu Arquivo|Novo. Imediatamente, uma nova base de conhecimento, vazia, será criada para você. 
	Finalmente, quando desejar salvar uma base na qual se está trabalhando para posterior uso, utiliza-se o menu Arquivo|Salvar. Quando você estiver salvando a base pela primeira vez, uma caixa de diálogo aparecerá para que seja indicado o nome do arquivo na qual a base será gravada e a pasta na qual ele será encontrada. Consulte a ajuda do Windows se precisar de maiores explicações sobre o uso da caixa de diálogo Salvar Arquivo.
	A impressão se dá através do menu Arquivo|Imprimir. Uma caixa de diálogo de configuração de impressoras aparecerá. Consulte a ajuda para detalhes de como usar a caixa de diálogo de configuração de impressoras.
	Muitas das opções dos menus encontram-se também na barra de ferramentas, inicialmente presente no topo da área de trabalho do Expert SINTA. É possível mudar a posição da barra de ferramentas clicando sobre ela e arrastando o mouse.


Opções relativas à gerência de bases.Opções relativas à gerência de bases.
	


2.2 A janela “Knowledge-in-a-box” (KIB)

Lista de regras: um duplo-clique abre a regra. É possível arrastar o nome da regra até a posição desejada, para alterar a ordem na qual elas aparecem.Lista de regras: um duplo-clique abre a regra. É possível arrastar o nome da regra até a posição desejada, para alterar a ordem na qual elas aparecem.Clique aqui para criar uma nova regra.Clique aqui para criar uma nova regra.Abre a regra que está selecionada na lista ao lado.Abre a regra que está selecionada na lista ao lado.Exclui a regra que está selecionada na lista ao lado.Exclui a regra que está selecionada na lista ao lado.Chama a janela de edição de variáveis.Chama a janela de edição de variáveis.Chama a janela de edição de interface.Chama a janela de edição de interface.Define os objetivos da base de conhecimento.Define os objetivos da base de conhecimento.Permite a inclusão de informações extras sobre a base.Permite a inclusão de informações extras sobre a base.Fecha a base de conhecimento. Somente uma pode estar aberta por vez.Fecha a base de conhecimento. Somente uma pode estar aberta por vez. 


	Essa janela traz todas as opções de edição de base, além de uma lista com todas as regras presentes até o momento. Tais opções, e outras, encontram-se repetidas na barra de ferramentas, inicialmente presente no topo da área de trabalho do Expert SINTA.


	Deixe o cursor do mouse momentaneamente sobre cada botão para exibir uma descrição breve. 

2.3 Criando variáveis

	Antes de podermos criar regras, é necessário que todas as variáveis utilizadas, bem como seus respectivos valores, sejam criados. Através desse mecanismo, a base fica organizada, fácil de manter e as regras podem ser criadas visualmente. Vejamos os passos necessários para criação e edição de variáveis e valores:

		Na janela da base de conhecimentos, clique o botão Variáveis;

		A janela de variáveis é aberta, e duas caixas de listagem podem ser vistas. A caixa da esquerda representa as variáveis já existentes, enquanto à da direita, os valores relativos à variável que está atualmente selecionada (clique em determinado item da lista de variáveis para exibir os valores correntes);

		Adicionar variáveis é simples: basta que pressione o botão Adicionar Variável e, em seguida, digitemos um valor na caixa de texto Variável. Atenção: Para confirmarmos o novo valor é necessário que se tecle <ENTER> imediatamente após a digitação ou que se pressione o pequeno botão à direita (o que possui a marca de verificação). Para cancelar a inserção, é só clicar em qualquer outro controle da janela (incluindo os botões OK e Cancelar, os quais fecham a janela).

		Adicionar valores é um procedimento análogo, só é necessário que uma variável esteja selecionada;

		Para indicar se uma determinada variável é numérica, multivalorada ou univalorada, primeiro selecionamos uma variável e, em seguida, o tipo desejado entre os botões de opção localizados no canto inferior direito da janela;

		Variáveis numéricas não podem ter valores pré-definidos. Nesse caso, a lista de valores passa atuar como lista de intervalo, ou seja, nela é exibido o intervalo de valores aceitáveis para aquela variável. Intervalos são incluídos de maneiras idênticas a valores, mas é necessário obedecer a certas regras:
	- Os intervalos devem ser da forma a;b ou ;b ou a;, onde a e b representam números reais . Assim sendo 
		a;b representa um número entre a e b;
		;b um número maior ou igual a b;
		a; um número menor ou igual a a;
Quando esses botões estão com a legenda em cinza, é porque realmente não é possível utilizá-los.  Se desejar utilizá-los, verifique o você está fazendo de diferente para isso ocorrer.Quando esses botões estão com a legenda em cinza, é porque realmente não é possível utilizá-los.  Se desejar utilizá-los, verifique o você está fazendo de diferente para isso ocorrer.	- Somente um intervalo pode ser incluído por variável numérica;
Nessa caixa aparecerão os valores relacionados àquela determinada variável.Nessa caixa aparecerão os valores relacionados àquela determinada variável.
Todas as variáveis incluídas aparecerão deste lado.Todas as variáveis incluídas aparecerão deste lado.


Veja a seção 1.3.4 para entender melhor a diferença entre variáveis multi e univaloradas.Veja a seção 1.3.4 para entender melhor a diferença entre variáveis multi e univaloradas.O botão marcado com “V” confirma alterações e equivale à tecla ENTER. O botão marcado com “X” cancela alterações e equivale à tecla ESC.O botão marcado com “V” confirma alterações e equivale à tecla ENTER. O botão marcado com “X” cancela alterações e equivale à tecla ESC.Pressione OK para fechar essa janela e confirmar as alterações feitas, não para efetuar uma única alteração. Para isso, use o botão pequeno marcado com um “V” verde ou a tecla ENTER.Pressione OK para fechar essa janela e confirmar as alterações feitas, não para efetuar uma única alteração. Para isso, use o botão pequeno marcado com um “V” verde ou a tecla ENTER.


	Obs1.: Mudar de variável não numérica para numérica pode implicar perda de valores.

	Obs2.: Apagar uma variável ou valor encontrado em uma regra ou mais regras implica que as mesmas também devem ser eliminadas. O Expert SINTA efetua uma confirmação antes de eliminá-las.

	Obs3.: Se você não definir nenhum valor para determinada variável, o Expert SINTA assume que ela é uma variável lógica, ou seja, que aceita apenas valores “Sim” e “Não”.

2.4 Definindo o(s) objetivo(s)

	O objetivo de uma consulta a um especialista é encontrar a resposta para um determinado problema. Assim também o é um sistema especialista. A diferença é que, aqui, os “problemas” são representados por variáveis. Antes de podermos executar nosso sistema pela primeira vez, é preciso que definamos quais são as variáveis (chamadas variáveis objetivo) que irão controlar o modo como a máquina de inferência se comporta.

	Sigamos o seguinte esquema para determinação de objetivos:

		Na janela de base de conhecimentos, pressionemos o botão Objetivos. Aparecerá uma janela com duas listas: a lista das variáveis comuns e a das variáveis objetivo;

		Para fazer de uma variável comum uma variável objetivo e vice-versa, selecione e variável desejada e clique no botão correspondente, aquele que aponta para a lista de destino. A lista com uma seta dupla (seja para a esquerda ou para direita) move todos os itens de uma lista para outra. Para mudar a ordem dos elementos da lista de objetivos, clique em um item e arraste-o até a posição desejada.

Lista das variáveis que podem ser utilizadas como objetivos do sistema especialista.Lista das variáveis que podem ser utilizadas como objetivos do sistema especialista.Lista das variáveis objetivo.Lista das variáveis objetivo.Deixe marcada este caixa para o Expert SINTA mostre em destaque os resultados atingidos do objetivo marcado acima. Para maiores detalhes, veja o capítulo III.Deixe marcada este caixa para o Expert SINTA mostre em destaque os resultados atingidos do objetivo marcado acima. Para maiores detalhes, veja o capítulo III.

	Observação: caso o sistema seja posto em funcionamento sem nenhum objetivo definido, nada acontecerá (seria como falar com um especialista sem ter nada para descobrir!)


Simulando encadeamento para frente

	O Expert SINTA trabalha naturalmente com encadeamento para trás, mas é possível manipular a máquina de inferência de modo a simular encadeamento para frente Para tal, as variáveis que se deseja encontrar inicialmente devem ser colocadas na lista de objetivos. Obviamente, como não é desejado que uma janela de resultados apareça a cada variável encontrada, é possível desabilitar essa exibição simplesmente desmarcando a caixa “Mostrar resultados” relativo à variável desejada na janela de edição de objetivos. Para maiores informações sobre a janela de exibição de resultados, consulte o capítulo III, Consultando o sistema especialista.

2.5 Trabalhando com regras

	O Expert SINTA utiliza regras de produção para modelar o conhecimento humano, o que o torna ideal para problemas de seleção, no qual uma determinada solução deve ser atingida a partir de um conjunto de seleções.
	Para excluir uma regra, selecione-a na janela da base de conhecimentos e pressione o botão Excluir. Para abrir uma regra, selecione-a na janela da base de conhecimentos e pressione o botão Abrir.

Para criar uma regra, faça os seguintes passos:

		Clique o botão Nova Regra, na janela da base de conhecimentos;

Indique a posição da regra (lembre-se que, em conhecimento procedural, a ordem das regras é importante). Se desejar, mude sua posição depois.Indique a posição da regra (lembre-se que, em conhecimento procedural, a ordem das regras é importante). Se desejar, mude sua posição depois.Se a regra que você está criando é muito parecida com outra, é possível reaproveitá-la. No exemplo, a regra 33 será criada a partir de ligeiras alterações na regra 28.Se a regra que você está criando é muito parecida com outra, é possível reaproveitá-la. No exemplo, a regra 33 será criada a partir de ligeiras alterações na regra 28.

		Um caixa de diálogo pedirá para que você indique a posição dessa regra em relação às demais (pois a ordem das regras influencia o descobrimento de soluções). Selecione a ordem desejada na lista;

Também é possível mudar a ordem da regra a partir daqui.Também é possível mudar a ordem da regra a partir daqui.
SOMENTE pressione esse botão QUANDO FINALIZAR a regra.SOMENTE pressione esse botão QUANDO FINALIZAR a regra.Embora não seja necessário, definir um nome para uma regra facilita sua identificação posterior.Embora não seja necessário, definir um nome para uma regra facilita sua identificação posterior.É aqui que apa-recem as senten-ças da sua regra. A sentença sele-cionada será aquela que sofre-rá alterações.É aqui que apa-recem as senten-ças da sua regra. A sentença sele-cionada será aquela que sofre-rá alterações.Deseja criar logo a próxima regra? Clique aqui. A regra aberta no momento será fechada.Deseja criar logo a próxima regra? Clique aqui. A regra aberta no momento será fechada.
Cada um desses botões efetua uma operação diferente com a sentença selecionada.Cada um desses botões efetua uma operação diferente com a sentença selecionada.


		Valor: clique na seta ao lado da caixa para exibir uma lista com todos os valores relacionados à variável escolhida ao lado.Valor: clique na seta ao lado da caixa para exibir uma lista com todos os valores relacionados à variável escolhida ao lado.Para inserir uma premissa, marque o local na caixa de listagem na qual se desejar inserir a sentença e clique o botão Inserir. Uma outra janela aparecerá com várias listas.
Operador de comparação: estabelece uma relação entre a variável e o valor (igualdade, diferença, etc.) à qual posteriormente será dado um grau de confiança.Operador de comparação: estabelece uma relação entre a variável e o valor (igualdade, diferença, etc.) à qual posteriormente será dado um grau de confiança.


Variável: clique na seta ao lado da caixa para exibir uma lista com as variáveis criadas.Variável: clique na seta ao lado da caixa para exibir uma lista com as variáveis criadas.
Conectivo: escolha o conectivo que irá ligar a sentença de regra às outras sentenças na mesma regra.Conectivo: escolha o conectivo que irá ligar a sentença de regra às outras sentenças na mesma regra.Negação: deixe marcada essa caixa para avaliar essa sentença por sua negativa. Equivale à inverter os operadores (transformar “=” em “<>”, “<=” em “>”, etc.)Negação: deixe marcada essa caixa para avaliar essa sentença por sua negativa. Equivale à inverter os operadores (transformar “=” em “<>”, “<=” em “>”, etc.)Aperte aqui quando terminar de inserir os itens desejados.Aperte aqui quando terminar de inserir os itens desejados.Aperte aqui para inserir uma nova premissa ou conclusão. Você poderá inserir várias sem necessidade de fechar esta janela, contanto que as premissas/conclusões inseridas sejam consecutivas.Aperte aqui para inserir uma nova premissa ou conclusão. Você poderá inserir várias sem necessidade de fechar esta janela, contanto que as premissas/conclusões inseridas sejam consecutivas. 


	


A primeira lista indica a variável a ser utilizada e a última o valor com o qual estabelecemos uma relação. É também possível identificar o tipo de relação: igualdade (“=”), diferença (“<>”), superioridade (“>” - somente para variáveis numéricas), entre outras. Caso a premissa não seja a primeira da regra, você deverá indicar o tipo de conectivo (E ou OU) pelo qual a premissa se liga à anterior. 

		Para inserir uma conclusão, proceda de maneira análoga. escolhendo um item a partir do conectivo ENTÃO na sua regra. Em conclusões, somente o operador de atribuição (“=”) pode ser utilizado. A última lista presente indica o grau de confiança daquela atribuição. Deixar a lista vazia indica uma confiança de 100% (cem por cento).


		Para editar uma sentença, selecione-a e clique o botão Alterar (ou dê um duplo clique sobre ela). Para eliminar uma sentença, selecione-a e clique o botão Excluir.

Aperte aqui para confirmar a mudança de um item, fechando a janela.Aperte aqui para confirmar a mudança de um item, fechando a janela.Fecha a janela sem efetuar alterações.Fecha a janela sem efetuar alterações.


	
Observação: também é possível visualizar regras em conjunto. Clique no botão Visualizar da janela KIB.


Como posso acelerar a digitação de regras?

	O ambiente do Expert SINTA permite a criação de regras sem grandes esforços. Basta que se sejam seguidas as dicas abaixo:

		Procure definir a priori todas as variáveis antes de iniciar as regras. Mas nada impede que uma regra seja iniciada e novas variáveis sejam definidas com a regra aberta. Apenas não esqueça de fechar a janela de variáveisvars com o botão OK antes de prosseguir;

		Use as duas mãos. Enquanto uma usa o mouse para selecionar um item na listas com as variáveis e seus valores, é possível acelerar digitando a primeira ou as primeiras letras que formam o nome de uma variável ou valor. Assim, as listas irão abrir próximas ao item desejado;

		Nessa ocasião, também é possível utilizar as setas do teclado “para cima” e “para baixo” para selecionar um item de uma lista. Digite a primeira ou as primeiras letras de um nome de uma variável/valor e, em seguida, use a seta “para baixo” até aparecer o nome desejado. Mesmo em bases grandes, esse é um método mais eficaz que digitar todo o nome;

		Use e abuse dos modelos. Eles existem para diminuir ao máximo seu trabalho. É muito comum em sistemas especialistas aparecerem regras derivadas de outras, portanto muito comum também será o uso de modelos;

		O botão “Nova...” na janela de edição de regras acelera bastante o processo. Quando você terminar a digitação de regra e desejar criar uma nova, aperte esse botão para uma nova surgir e a antiga ser fechada, tudo em um passo só;

2.6 Definindo interface com o usuário

	Um sistema especialista implementado com o Expert SINTA comunica-se com o usuário final através de menus de múltipla escolha (ou escolha simples, se a variável em questão for univalorada). Estes menus são construídos automaticamente pela shell, mas alguns detalhes devem ser fornecidos pelo criador da base. Os elementos da interface com o usuário são os seguintes:


Entrada para grau de confiança: construído automaticamente.Entrada para grau de confiança: construído automaticamente.


Pergunta realizada pelo sistema ao usuário.Pergunta realizada pelo sistema ao usuário.Opções referentes àquela variável. Foram definidas na janela de Variáveis.Opções referentes àquela variável. Foram definidas na janela de Variáveis.Indicativo de que a variável é multi ou univalorada. Definido na janela de Variáveis.Indicativo de que a variável é multi ou univalorada. Definido na janela de Variáveis.Explicação rápida sobre aquela pergunta.Explicação rápida sobre aquela pergunta.

	Por exemplo, a pergunta realizada pela máquina de inferência deve ser personalizada para que seja inteligível. Quando o Expert SINTA necessita perguntar o valor de determinada variável,  ele cria uma sentença genérica do tipo “Qual o valor de x?”, onde x é o nome da variável. Deve-se criar uma sentença mais clara através do editor de interface.

	Para isso, clique no botão Interface da janela KIB. O Expert SINTA permite modificações em apenas dois itens: perguntas e explicações rápidas.
Use esses botões para mandar elementos de uma lista para outra.Use esses botões para mandar elementos de uma lista para outra.
Variáveis que ainda não possuem perguntas relacionadas.Variáveis que ainda não possuem perguntas relacionadas.Variáveis que já possuem perguntas.Variáveis que já possuem perguntas.


Aqui são modificadas as perguntas. As mudanças são acionadas automatica-mente.Aqui são modificadas as perguntas. As mudanças são acionadas automatica-mente.Explicações sobre a pergunta, motivo pelo qual é feita, entre outras informações.Explicações sobre a pergunta, motivo pelo qual é feita, entre outras informações.É permitido usar graus de confiança na resposta? Então, marque esta caixa.É permitido usar graus de confiança na resposta? Então, marque esta caixa.

		Na parte superior direita da janela, encontra-se uma lista das variáveis que já possuem perguntas prontas. Para visualizá-las, clique em alguma. Para modificar a pergunta ou o motivo, simplesmente rescreva o conteúdo das caixas de texto. As mudanças são automáticas. Para eliminar uma pergunta, simplesmente mova o item selecionado para a lista da esquerda, pressionando o botão correspondente;

		Na lista superior esquerda, aparecem as variáveis que ainda não possuem nenhuma pergunta pronta. Para criar uma nova pergunta, selecione a variável desejada e mova-o para a lista da direita, pressionando o botão correspondente, e digite o conteúdo das caixa de texto. As mudanças são automáticas.

2.7 Informações adicionais sobre a base

	A janela Informações sobre a Base, exibida a partir do botão Informações da janela KIB, permite a definição de informações esclarecedoras sobre a base, indicação dos autores e definição de contextos de ajuda que podem ser vitais para o aproveitamento do sistema especialista. A janela se divide em duas partes:

		Geral: Aqui, informações iniciais sobre o sistema são incluídas, tais como o nome da base, os autores e um texto de abertura. Todo os dados aparecerão em uma tela de abertura quando a base é colocada em funcionamento. Para permitir a exibição desses dados, deixe marcada a caixa de verificação Exibir tela de abertura, na parte inferior da janela.

Deixe marcada se quiser que uma tela de abertura com essas informações apareça no início de cada consulta.Deixe marcada se quiser que uma tela de abertura com essas informações apareça no início de cada consulta.

		Arquivo de Ajuda: um bom sistema especialista não deve saber somente encontrar respostas, mas também fazer o usuário utilizar a resposta com fins práticos. Para isso, o Expert SINTA possibilita a inclusão de tópicos de ajuda para os valores possíveis de determinados atributos, associando a um arquivo no formato Ajuda do Windows a sua base. Explicações sobre a criação de arquivos de ajuda fogem ao escopo do presente documento, mas existem diversas documentações fornecidas por terceiros. O Expert SINTA também não distribui o compilador necessário para a criação do arquivo final.

	A idéia desses textos explicativos não é somente ativar uma ajuda on-line quando o usuário estiver utilizando o sistema, mas ir diretamente ao tópico explicativo de uma resposta, quando o Expert SINTA atinge um determinado objetivo. Para isso, o desenvolvedor deve, na janela Informações sobre a Base, escolher os valores das variáveis-objetivo que irão possuir um número de contexto de ajuda. Quando o usuário final ativar a ajuda on-line, ela aparecerá diretamente no tópico referente à solução específica. O desenvolvedor também deve incluir o nome do arquivo de ajuda na caixa de texto nome do arquivo de ajuda, na mesma janela.

	Por exemplo, no caso da configuração de redes de computadores, o protocolo ideal sugerido pelo sistema foi o TCP/IP. Então, bastará ao usuário selecionar esse item e acionar a ajuda que imediatamente aparecerão informações sobre tal protocolo. 

	Maiores informações sobre números de contexto de ajuda e compiladores de Windows Help  (como o HC31 ou o Microsoft Help Workshop) podem ser conseguidas através de terceiros.

Digite aqui o nome do arquivo de ajuda.Digite aqui o nome do arquivo de ajuda.Para garantir melhor aproveitamento da sua base, textos didáticos podem ser relacionados a cada solução encontrada. Para tanto, crie um arquivo de ajuda no formato Ajuda do Windows e defina os contextos de ajuda relativos a cada valor (se desejado) de cada variável objetivo.


Selecione a variável objetivo desejada aqui...Selecione a variável objetivo desejada aqui......e o valor correspondente aqui....e o valor correspondente aqui.Entre o número de contexto de ajuda daquele valor e pressione <ENTER> ou o pequeno botão verde ao lado.Entre o número de contexto de ajuda daquele valor e pressione <ENTER> ou o pequeno botão verde ao lado.Desfaz a última alteração.Desfaz a última alteração.Confirma mudanças.Confirma mudanças.Só pressione aqui quando concluir tudo.Só pressione aqui quando concluir tudo.


2.8 Opções

	Para permitir maior flexibilidade na construção de um sistema especialista, o Expert SINTA oferece diversas opções de configuração. Para acessá-las, utilize o menu Exibir|Opções. Uma caixa de diálogo com três partes aparecerá.

		Dependendo do modo como a precedência for organizada ou como os fatores de confiança forem definidos, a máquina de inferência pode saber que uma regra não vai atingir o grau mínimo de confiança para ser aceita antes mesmo de avaliá-la por completo. Se você desejar que as regras sejam sempre avaliadas completamente, mesmo quando já se sabe se serão aceitas ou não, marque esta caixa.Dependendo do modo como a precedência for organizada ou como os fatores de confiança forem definidos, a máquina de inferência pode saber que uma regra não vai atingir o grau mínimo de confiança para ser aceita antes mesmo de avaliá-la por completo. Se você desejar que as regras sejam sempre avaliadas completamente, mesmo quando já se sabe se serão aceitas ou não, marque esta caixa.Máquina de inferência: permite pequenas modificações no comportamento da máquina de inferência, como o modo pelo qual as premissas são avaliadas.
Defina aqui qual a precedência dos operadores lógicos E e OU. Lembre-se da diferença: na expressão A e B ou C, se mativermos a precedência no “e”, ela pode ser intepretada como (A e B) ou C. Se a precedência for do “ou”, A e (B ou C).Defina aqui qual a precedência dos operadores lógicos E e OU. Lembre-se da diferença: na expressão A e B ou C, se mativermos a precedência no “e”, ela pode ser intepretada como (A e B) ou C. Se a precedência for do “ou”, A e (B ou C).


		Fatores de confiança: após avaliar todas as premissas de uma regra, o Expert SINTA pode aceitá-la ou rejeitá-la. Se, ao calcular o fator de confiança final ele for maior que o mínimo estipulado, a regra será aceita. É nessa janela que se define o valor mínimo. Também é aqui que se pode personalizar as funções de cálculo de fatores de confiança.


Aqui é definido o valor de confiança mínimo para que uma regra seja aceita. Coloque na escala percentual: mínimo 0 e máximo 100.Aqui é definido o valor de confiança mínimo para que uma regra seja aceita. Coloque na escala percentual: mínimo 0 e máximo 100.O Expert SINTA utiliza algumas funções para calcular fatores de confiança em determinados eventos. Estes eventos podem ser uma conjunção, uma disjunção ou uma regra aceita. Pode-se usar as funções padrões do Expert SINTA ou criar novas. Cada função apresenta dois parâmetros implícitos, cnf1 e cnf2 (ou seja, o grau de confiança do primeiro termo da conjunção e o grau de confiança do segundo termo da conjunção, respectivamente). Para, por exemplo, mudar a função da conjunção para o mínimo entre os dois valores (aperte o botão Alterar se a fórmula padrão estiver sendo utilizada) ao lado da função de conjunção e digite Min(cnf1, cnf2). Para retorna à forma padrão, aperte o botão respectivo. Maiores informações sobre fórmulas posteriormente neste capítulo (Criando fórmulas).O Expert SINTA utiliza algumas funções para calcular fatores de confiança em determinados eventos. Estes eventos podem ser uma conjunção, uma disjunção ou uma regra aceita. Pode-se usar as funções padrões do Expert SINTA ou criar novas. Cada função apresenta dois parâmetros implícitos, cnf1 e cnf2 (ou seja, o grau de confiança do primeiro termo da conjunção e o grau de confiança do segundo termo da conjunção, respectivamente). Para, por exemplo, mudar a função da conjunção para o mínimo entre os dois valores (aperte o botão Alterar se a fórmula padrão estiver sendo utilizada) ao lado da função de conjunção e digite Min(cnf1, cnf2). Para retorna à forma padrão, aperte o botão respectivo. Maiores informações sobre fórmulas posteriormente neste capítulo (Criando fórmulas).


		Senha: um sistema especialista não deixa de ser um programa de computador. Portanto, pode se fazer necessário algum tipo de proteção sobre este software. O Expert SINTA permite três níveis de proteção:

Permitir execução e visualização
		O usuário do seu sistema especialista não tem permissão para modificar a base, mas pode executá-la, depurá-la e imprimi-la. É uma opção para evitar que estranhos modifiquem a base, fazendo com que perca sua consistência.

Permitir somente execução
		Um pouco mais polêmica, essa opção permite que o usuário sem senha apenas possa executar o sistema especialista, mas não pode modificá-la e menos ainda visualizá-la. Porém, um sistema especialista que não exibe o conteúdo de suas regras, não permite acompanhar sua execução e verificar como atingiu o resultado exibido não merece a mesma confiabilidade de um sistema que permite o completo acesso às suas regras. O principal motivo que levaria um projetista a escolher essa opção de restrição é o perigo de pirataria do conteúdo do sistema especialista. Mas, recomenda-se esquecer o risco e dar prioridade à confiabilidade de seu trabalho. Além disso, um sistema especialista que não é protegido por nenhuma lei de direitos autorais dá sempre a impressão de não ser tão profissional quanto deveria.

Nenhuma permissão
		Somente pessoas com senha podem utilizar esse sistema especialista.

O uso de senhas para proteção da base de conhecimento só se efetivará de verdade quando esta caixa de verificação estiver marcada.O uso de senhas para proteção da base de conhecimento só se efetivará de verdade quando esta caixa de verificação estiver marcada.Define diferentes níveis de acesso que uma pessoa que não dispõe de senha tem direito em relação ao sistema especialista.Define diferentes níveis de acesso que uma pessoa que não dispõe de senha tem direito em relação ao sistema especialista.Clique aqui para chamar a caixa de diálogo de definição de senhas para criar ou apagar uma senha que esteja protegendo o sistema especialista.Clique aqui para chamar a caixa de diálogo de definição de senhas para criar ou apagar uma senha que esteja protegendo o sistema especialista.


	O Expert SINTA não pretende criar proteções realmente seguras para seu sistema, mas apenas desencorajar modificações não autorizadas. Se você realmente deseja criar um senha, clique no botão Nova Senha... (ele se chama Mudar Senha... caso alguma senha já esteja sendo utilizada). Uma caixa de diálogo aparecerá:
Digite aqui a senha que irá proteger seu sistema. Note que ela não irá aparecer na tela.Digite aqui a senha que irá proteger seu sistema. Note que ela não irá aparecer na tela.
Repita a senha que foi digitada acima para sua maior segurança, já que não se está vendo qual a palavra-chave.Repita a senha que foi digitada acima para sua maior segurança, já que não se está vendo qual a palavra-chave.Se você não quiser mais nenhuma senha no seu sistema, clique esse botão.Se você não quiser mais nenhuma senha no seu sistema, clique esse botão.


2.9 Criando fórmulas

	Quando se utiliza variáveis numéricas, é comum que queiramos atribuir a elas valores extraídos de fórmulas matemáticas, ou compará-las a valores equivalentes. Se você já sabe como criar regras, já deve saber como inserir uma sentença de regra contendo uma variável numérica e um valor. Para inserir uma função ou expressão, simplesmente digite-a no lugar reservado ao valor. Deve-se, porém, seguir as seguintes regras:

		Os operadores matemáticos válidos são: + (adição), - (subtração), * (multiplicação), / (divisão), + (sinal unário indicando que um número é positivo), - (sinal unário indicando que um número é negativo);

		As seguinte funções podem ser utilizadas:
- Min(a, b): retorna o menor valor entre a e b;
- Max(a, b): retorna o maior valor entre a e b;
- Sqr(x): raiz quadrada de x;
- Exp(x): exponencial de x;
- Pot(a, b): retorna a elevado a b;
- Log(a, b): logaritmo de a na base b;
- Ln(x): logaritmo natural de x;
- Abs(x): módulo (valor absoluto) de x;
- Sen(x): seno de x, x em radianos;
- Cos(x): cosseno de x, x em radianos;
- Tan(x): tangente de x, x em radianos;

		Variáveis podem ser utilizadas nas expressões. Para atribuir a raiz de uma variável a outra, pode-se fazer algo do tipo a = sqr(b). A diferença entre maiúsculas e minúsculas não é considerada. Atenção: se o nome da variável contiver espaços em branco ou símbolos, coloque-a na expressão entre aspas. Por exemplo: Min(“juros de dezembro”, “juros de janeiro”);

		Se o valor da expressão for desconhecidodesconhecido, uma variável que esteja recebendo o valor retornado também será instanciada para desconhecido;

		É possível utilizar parênteses e aninhar funções.

2.10 Sumário

	Neste capítulo, vimos:

		Como tratar com os arquivos de base de conhecimento, os denominados arquivos BCM;

		Quais são as entidades que formam a base de conhecimento: variáveis, objetivos, regras, perguntas, informações adicionais;

		A janela Knowledge-in-a-Box e os editores de entidades do Expert SINTA.

		Opções de configuração e senhas;

		Usando fórmulas matemáticas;

	A esta altura, você está apto a criar seu “especialista digital”!


Capítulo III - Consultando o sistema especialista

Como utilizar um sistema especialista construído com o Expert SINTA, além de permitir entender o seu funcionamento.

	O Expert SINTA mantém uma interface uniforme para a consulta de qualquer base de conhecimento criada em seu ambiente. Existem dois modos pelos quais podem ser efetuadas consultas: 		a execução usual, na qual o usuário acompanha uma sequência de menus de múltipla (ou única) escolha, nos quais deve-se indicar informações que resultarão nas conclusões atingidas pelo sistema especialista;		o modo de acompanhamento, pelo qual é possível examinar o conteúdo das regras que formam o sistema, bem como acompanhar a execução passo a passo e as instâncias (valores) que cada variável possui em determinado momento. Para utilizar este modo, é preciso que o projetista da base não tenha protegido o acesso às regras por meio de uma senha	O Expert SINTA mantém uma interface uniforme para a consulta de qualquer base de conhecimento criada em seu ambiente. Existem dois modos pelos quais podem ser efetuadas consultas: 		a execução usual, na qual o usuário acompanha uma sequência de menus de múltipla (ou única) escolha, nos quais deve-se indicar informações que resultarão nas conclusões atingidas pelo sistema especialista;		o modo de acompanhamento, pelo qual é possível examinar o conteúdo das regras que formam o sistema, bem como acompanhar a execução passo a passo e as instâncias (valores) que cada variável possui em determinado momento. Para utilizar este modo, é preciso que o projetista da base não tenha protegido o acesso às regras por meio de uma senha


	Um sistema especialista procura atingir conclusões para determinados objetivos. Sempre que um desses objetivos é atingido, ou quando se esgotam todas as possibilidades, o Expert SINTA apresentará uma janela com os resultados e o acompanhamento de como se chegou àquela conclusão (também é necessário que o projetista não tenha restringido o acesso).	Alguns sistemas também devem trazer um sistema de ajuda on-line, pelo qual serão dadas maiores informações de como se deve melhor utilizar as respostas conseguidas, bem como explicações mais detalhadas sobre as funções daquele determinado sistema especialista.	Um sistema especialista procura atingir conclusões para determinados objetivos. Sempre que um desses objetivos é atingido, ou quando se esgotam todas as possibilidades, o Expert SINTA apresentará uma janela com os resultados e o acompanhamento de como se chegou àquela conclusão (também é necessário que o projetista não tenha restringido o acesso).	Alguns sistemas também devem trazer um sistema de ajuda on-line, pelo qual serão dadas maiores informações de como se deve melhor utilizar as respostas conseguidas, bem como explicações mais detalhadas sobre as funções daquele determinado sistema especialista.
3.1 Conceitos rápidos

	Estes conceitos são para entendimento das seções a seguir. Se você leu os capítulos anteriores, encontrará alguns termos repetidos:

		projetista do conhecimento: é o encarregado de transporta o conhecimento humano para uma série de passos que um computador é capaz de entender;

		base de conhecimento: é o conjunto de informações, representado no Expert SINTA na forma de regras SE-ENTÃO, as quais supostamente devem agir conforme um especialista humano. Seria, então, a “alma” do sistema especialista;

		variáveis: são os elementos do mundo real representados na base de conhecimento, como, por exemplo, uma tipo de doença, a quilometragem de um carro, pragas de um cajueiro, a posse ou não de crédito em determinada agência bancária, etc.

		valores: são instâncias das variáveis, ou seja, uma variável pode possuir um ou mais valores (quem decide é o projetista do conhecimento). Também podem existir casos nos quais uma variável permanece DESCONHECIDA, ou seja, o sistema não conseguiu nenhum valor satisfatório para ela;

		depuração: acompanhamento da execução da consulta com o intuito de compreender como o sistema especialista chegou àquela(s) conclusão(ões). Também pode ser usada com o intuito de corrigir falhas na base de conhecimento;

3.2 Guia visual de operações

	Existem dois modos pelos quais se pode acompanhar uma consulta no Expert SINTA. Utilizando o menu da janela principal ou a barra de ferramentas. As operações de execução/depuração podem ser:

		iniciar a consulta;

		interromper a execução para saber que regra está utilizando naquele ponto;

		executar passo a passo;

		adicionar elementos da base (variáveis) para acompanhar os valores que eles recebem durante a consulta;

		ver esses valores escolhidos;

		abandonar a consulta.

	Os comandos equivalentes na barra de ferramentas são:
Interrompe a execução da base, exibindo as regras no modo de acompanhamento.Interrompe a execução da base, exibindo as regras no modo de acompanhamento.Cancela a consulta. O sistema pedirá confirmação.Cancela a consulta. O sistema pedirá confirmação.


Inicia a execução/termina o modo de acompanhamento e retorna ao modo de execução.Inicia a execução/termina o modo de acompanhamento e retorna ao modo de execução.Estando no modo de acompanhamento, avança sentença por sentença na base de conhecimento (também conhecido como execução passo a passo).Estando no modo de acompanhamento, avança sentença por sentença na base de conhecimento (também conhecido como execução passo a passo).Visualiza todas os valores das variáveis que você escolheu.Visualiza todas os valores das variáveis que você escolheu.Escolhe variáveis para que sejam exibidos seus valores durante uma consulta.Escolhe variáveis para que sejam exibidos seus valores durante uma consulta.Volta para a última pergunta realizada ao usuário pelo sistema.Volta para a última pergunta realizada ao usuário pelo sistema.


						
			
	figura 3.1 - A barra de ferramentas para execução de consultas

			

	A seguir, são especificados os comandos equivalentes através do menu. Aqueles que estiverem em cinza não estão disponíveis naquele momento. Os itens de menu não explicados aparecem em figuras anteriores. Alguns itens apresentam um nome representando uma tecla (ou uma combinação de teclas) que pode ser utilizada em substituição ao menu. Consulte a ajuda do Windows para maiores informações sobre teclas de atalho.


Inicia a execução do sistema especialista. Inicia a execução do sistema especialista. Inicia a execução no modo de acompanhamento. Inicia a execução no modo de acompanhamento. Antes do início da consulta:

Exibe os valores das variáveis que você escolheu. Exibe os valores das variáveis que você escolheu. 
Escolhe variáveis para exibição. Escolhe variáveis para exibição.  Escolhe variáveis para exibição.  Escolhe variáveis para exibição.	


Coloca o sistema no modo de acompanhamento. Coloca o sistema no modo de acompanhamento. Durante o modo de execução :


Encerra a consulta antes do final. Encerra a consulta antes do final.  Encerra a consulta antes do final.  Encerra a consulta antes do final.	

Durante o modo de acompanhamento:
Retorna ao modo de execução. Retorna ao modo de execução. 

	


figura 3.2 - Os itens de menu para execução de consultas

3.3 A consulta

	Iniciando-se a consulta, seja no modo de execução ou acompanhamento, o sistema especialista poderá apresentar uma tela de abertura, com informações sobre seu funcionamento, os autores e casos aos quais ele se aplica. Leia atentamente esse texto de abertura, pois poderá trazer informações úteis. Se o botão de ajuda estiver disponível (aquele situado no canto inferior direito), aperte-o se desejar informações mais detalhadas.
	Para iniciar a consulta, pressione o botão OK. O botão Cancelar abandona a execução do sistema especialista. O botão Ajuda chama a ajuda da base, se ela estiver disponível.


figura 3.3 - Uma abertura típica de um sistema especialista

	Obs.: Nem todas os sistemas trazem uma abertura, pois sua criação depende do projetista do conhecimento.

	A consulta se desenvolve por meio de menus de múltipla (ou única) escolha. Um menu típico é mostrado na figura 3.4. A figura 3.5 mostra um menu para entrada de valores numéricos.
Preste atenção quantas opções são passíveis de escolha.Preste atenção quantas opções são passíveis de escolha.Qual o grau de confiança da sua resposta? Coloque aqui, em porcenta-gem. Qual o grau de confiança da sua resposta? Coloque aqui, em porcenta-gem. 


As opções de escolha aparecem dentro desse grupo de caixas de verificação. As opções de escolha aparecem dentro desse grupo de caixas de verificação. Pressione aqui para confirmar a sua resposta. Pressione aqui para confirmar a sua resposta. Se esse botão estiver habilitado, é possível ler algumas explicações extras sobre a importância daquela pergunta. Se esse botão estiver habilitado, é possível ler algumas explicações extras sobre a importância daquela pergunta. 


figura 3.4 - Uma pergunta realizada pelo sistema a você


figura 3.5 - Uma pergunta sobre um valor numérico

	Escolha a(s) opção(ões) desejada(s) marcando as respectivas caixas de verificação, localizadas sempre  à esquerda de cada alternativa. Observe, à direita, na figura 3.4, a possibilidade de entrar com o grau de confiança da sua resposta. Graus de confiança são utilizados quando não se possui certeza absoluta sobre um fato. Assim sendo, você pode expressar sua dúvida por meio de um número percentual, ou seja, a sua resposta terá validade no intervalo de zero a cem por cento. Cada resposta assinalada pode ter o seu próprio grau de confiança.
	Caso realmente não saiba responder àquela pergunta, simplesmente deixe todas as alternativas em branco. O Expert SINTA entenderá que você não sabe como responder ao questionamento apresentado.
	Não esqueça que, a qualquer momento, é possível mudar o modo de execução da base ou interrompê-la usando o menu ou a barra de ferramentas.

3.4 As janelas de acompanhamento

	Quando se está no modo de acompanhamento, uma nova janela sempre estará aberta, o depurador (figura 3.6).


figura 3.6 - O depurador

	O depurador exibe, em sua caixa de listagem, todas as regras da base de conhecimento. A sentença em destaque é aquela que está sendo analisada no momento pela máquina de inferência. Observe que é possível rolar a lista para cima e para baixo, exibindo o resto da base, mas somente a linha representando o ponto de parada pode ser selecionada.

	Utilize o menu Depurar|Passo, ou o botão respectivo da barra de ferramentas, ou ainda a tecla F8 para continuar a acompanhar, passo a passo, a execução do sistema. Se o sistema estiver realizando uma pergunta para você, primeiro responda antes de executar um passo, ou nada acontecerá.

	Fechar o Depurador equivale a voltar ao modo de execução. Se você deseja abandonar a consulta, utilize os métodos explicados anteriormente neste capítulo.

	Para acompanhar mudanças nas instanciações das variáveis, primeiro deve-se escolher quais delas se deseja que sejam acompanhadas. Use o menu Depurar|Adicionar Variáveis..., ou o botão respectivo. A caixa de diálogo da figura 3.7 aparecerá.

Só serão exibidas as variáveis que ainda não foram adicionadas. Só serão exibidas as variáveis que ainda não foram adicionadas.  
figura 3.7 - Caixa de diálogo Adicionar Variáveis

	Selecione uma ou mais variáveis. Mantenha pressionada a tecla CONTROL para selecionar mais de uma. Use a tecla SHIFT para selecionar uma seqüência. Ao apertar o botão OK, elas irão para a lista de variáveis acompanhadas.

	É possível exibir essa lista a qualquer momento. Use o menu Depurar|Variáveis, ou o botão respectivo da barra de ferramentas, ou ainda as teclas CONTROL e R ao mesmo tempo.


figura 3.8 - A lista de variáveis acompanhadas

	Sempre que a consulta alterar uma dessa variáveis, essa janela atualizar-se-á automaticamente. Para retirar uma variável da lista de acompanhamento, selecione-a com o mouse e pressione a tecla DELETE.

	Relembrando: para poder utilizar livremente as janelas de acompanhamento, é necessário que o projetista da base não tenha inserido uma proteção por senha.

3.5 Compreendendo os resultados atingidos

	Como foi dito anteriormente, o sistema especialista divide a sua consulta em uma busca de objetivos. Ao final de cada busca, uma janela de resultados é apresentada. Ela se divide em quatro partes:

		Resultados: são apresentados todos os valores atingidos pelo objetivo, com os respectivos graus de confiança. Caso o sistema não tenha chegado a nenhuma solução, ele indicará. Observe também o pequeno botão localizado no canto superior esquerdo, marcado com um ponto de interrogação. Se ele estiver habilitado, significa que o projetista da base também criou uma ajuda sobre o sistema especialista. Assim sendo, selecione no grid de qual solução você deseja maiores explicações (um leve retângulo envolve a seleção). Em seguida, aperte esse botão, e um texto explicativo sobre o que você deve fazer com essa solução aparecerá. Ou dê um clique duplo sobre a resposta, ou ainda pressione F2 com a solução desejada selecionada. O botão Ajuda localizado à direita apenas mostra como se deve utilizar a janela de resultados;


O objetivo em questão. O objetivo em questão. Indica o grau de confiança das soluções respectivas. Indica o grau de confiança das soluções respectivas. 

O botão está habilitado, indicando que esse sistema especialista possui ajuda. O botão está habilitado, indicando que esse sistema especialista possui ajuda. Este item está selecionado Este item está selecionado 

figura 3.9 - Os resultados atingidos

		Histórico: exibe todo o caminho realizado pelo sistema especialista até atingir àquela(s) solução(ões). Mova a lista para cima e para baixo para visualizar o resto. Clique nos ícones para fechar regras ou abri-las, facilitando a visualização. Esta página só está disponível livremente se o projetista da base não houver protegido a base de conhecimento;
Clique aqui para expandir todas as sentenças. Clique aqui para expandir todas as sentenças. 
Clique aqui para fechar todos os itens, exibindo apenas a chama-da de cada regra. Clique aqui para fechar todos os itens, exibindo apenas a chama-da de cada regra. Clique aqui rolar a árvore para cima e para baixo. Clique aqui rolar a árvore para cima e para baixo. 

figura 3.10 - A árvore de pesquisa

		Todos os valores: é uma generalização da primeira página. Exibe todos os valores de todas as variáveis. Dê um clique duplo na figura indicando a variável para exibir ou esconder todos os seus valores. Também só está disponível com permissão do projetista.


Clique aqui para mostrar os valores de todas as variáveis.Clique aqui para mostrar os valores de todas as variáveis.Clique aqui para esconder todos os valores. Clique aqui para esconder todos os valores. Dê um clique duplo nessa figurinha para abrir a variável. Dê um clique duplo nessa figurinha para abrir a variável. 

figura 3.11 - Todos os valores atingidos pelo sistema especialista

		O sistema: exibe as todas as regras do sistema especialista. Útil também para entender como o sistema chegou àquela(s) conclusão(ões), em conjunto com o Histórico.


figura 3.12 - A base de conhecimento

	
	Essa janela pode aparecer em uma consulta tantas vezes quanto for o número de objetivos a serem alcançados.
	Pressione o botão Fechar para continuar a consulta. Parabéns, você agora deve saber tudo sobre a utilização de um sistema especialista construído com o Expert SINTA.

3.6 Sumário

	Neste capítulo, vimos:

		Revisão de conceitos básicos sobre variáveis e valores;

		Itens de menu e botões de atalho para ações de consulta;

		Estrutura da janela de perguntas do Expert SINTA;

		Acompanhamento da consulta;

		A janela de resultados.

	Você pode utilizar este capítulo para a elaboração de manuais dos sistemas especialistas de sua autoria!


Agradecimentos

	O Expert SINTA foi criado pelo Grupo SINTA (Sistemas INTeligentes Aplicados), um projeto UFC / UECE. A presente ferramenta foi o fruto do primeiro ano de trabalho desse grupo. O grupo espera receber várias críticas e sugestões sobre o que foi apresentado até o momento. O contato pode ser feito pelo endereço WWW do Laboratório de Inteligência Artificial (LIA) - http://www.lia.ufc.br.

Grupo SINTA - Sistemas INTeligentes Aplicados
Coordenação geral: Prof. José Helano Matos Nogueira
Integrantes:
	João Fernando Lima Alcântara
	Rafael Castro de Andrade
	Ricardo Bezerra de Andrade e Silva
Expert SINTA - idéia original de Ricardo Sousa Silvestre, ex-integrante, 1995

SECAJU  - Sistema especialista em  pragas e doenças do cajueiro - é de autoria dos seguintes membros do CNPAT:
	Quélzia Maria Silva Melo  (Doutora em Entomologia)
	Antonio Lindemberg M. Mesquita (Mestre em Entomologia)
	Lianna Maria Saraiva Teixeira (Mestre em Fitossanidade)
	Francisco das Chagas O. Freire (Doutor em Fitopatologia).
SECAJU - CNPAT, 1988 - 1996

Agradecimentos especiais: 
	Genaro França - EMBRAPA 
	Marcelino Pequeno - UFC
	Tarcísio Pequeno - UFC

Expert SINTA
1995, 1996 - Laboratório de Inteligência Artificial/LIA-UFC
Projeto financiado pela CAPES e CNPq.

Apêndice A - Sistema de Diagnóstico de Pragas e Doenças do Cajueiro (SECAJU)

A seguir, as regras que compõem o SECAJU, base de conhecimento de autoria de membros do CNPAT.

Objetivos:
praga
suspeita de praga
doença 
suspeita de doença

Regras:

Regra 1
SE castanha broqueada com amêndoa totalmente destruída = Sim
ENTÃO praga = traça das castanhas CNF 100%

Regra 2
SE galerias nos ramos ou inflorescências = Sim
ENTÃO praga = broca das pontas CNF 100%

Regra 3
SE inflorescências = Murchas ou secas
E  brotações novas murchas = Sim
E  orifícios laterais nos ramos ou inflorescências = Sim
ENTÃO praga = broca das pontas CNF 100%

Regra 4
SE inseto mole amarelo ou verde = Sim
ENTÃO praga = pulgão das inflorescências CNF 100%

Regra 5
SE ácaro estreito ou longo nas inflorescências = Sim
ENTÃO praga = eriofiídeo das flores CNF 100%

Regra 6
SE inseto com cinta avermelhada sob as folhas = Sim
ENTÃO praga = tripes CNF 100%

Regra 7
SE lagarta cinza lisa com partes pretas e manchas brancas = Sim
OU lagarta enrolada em folha = Sim
OU espirais de ovos = Sim
ENTÃO praga = lagarta saia justa CNF 100%

Regra 8
SE lagarta grande verde - alaranjada = Sim
ENTÃO praga = lagarta verde CNF 100%

Regra 9
SE lagarta verde com pêlos avermelhados e urticantes = Sim
ENTÃO praga = lagarta das folhas CNF 100%

Regra 10
SE lagarta pequena com pêlos longos e verdes = Sim
ENTÃO praga = lagarta véu de noiva CNF 100%

Regra 11
SE besourinhos vermelhos = Sim
OU larvas marrom - claras e lentas = Sim
ENTÃO praga = besouro vermelho CNF 100%

Regra 12
SE secreção pulverulenta branca sob as folhas = Sim
ENTÃO praga = mosca branca CNF 100%

Regra 13
SE folhas do broto terminal repolhadas = Sim
ENTÃO praga = larva do broto terminal CNF 100%

Regra 14
SE espuma sobre inflorescências ou maturis = Sim
ENTÃO praga = cigarrinha CNF 100%

Regra 15
SE praga <> broca das pontas
E  goma nos ramos = Sim
ENTÃO suspeita de praga = broca das pontas CNF 30%

Regra 16
SE praga <> broca das pontas
E  brotações novas murchas = Sim
ENTÃO suspeita de praga = broca das pontas CNF 40%

Regra 17
SE praga <> broca das pontas
E  inflorescências = Murchas ou secas
ENTÃO suspeita de praga = broca das pontas CNF 40%

Regra 18
SE praga <> pulgão das inflorescências
E  inflorescências = Murchas ou secas
E  maturis = Deformados
ENTÃO suspeita de praga = pulgão das inflorescências CNF 20%

Regra 19
SE praga <> pulgão das inflorescências
E  mela nas inflorescências, folhas e maturis = Sim
ENTÃO suspeita de praga = pulgão das inflorescências CNF 20%


Regra 20
SE praga <> eriofiídeo das flores
E  queda das inflorescências = Sim
ENTÃO suspeita de praga = eriofiídeo das flores CNF 50%

Regra 21
SE praga <> eriofiídeo das flores
E  inflorescências cloróticas = Sim
ENTÃO suspeita de praga = eriofiídeo das flores CNF 70%

Regra 22
SE praga <> tripes
E  folhas = Com coloração prateada
ENTÃO suspeita de praga = tripes CNF 45%

Regra 23
SE praga <> lagarta saia justa
E  praga <> lagarta das folhas
E  praga <> lagarta verde
E  praga <> lagarta véu de noiva
E  praga <> besouro vermelho
E  diminuição da área foliar = Sim
ENTÃO suspeita de praga = lagarta saia justa CNF 50%
      suspeita de praga = lagarta da folhas CNF 50%
      suspeita de praga = lagarta verde CNF 50%
      suspeita de praga = lagarta véu de noiva CNF 50%
      suspeita de praga = besouro vermelho CNF 50%

Regra 24
SE praga <> lagarta saia justa
E  teias envolvendo inflorescências e brotações novas = Sim
ENTÃO suspeita de praga = lagarta saia justa CNF 100%

Regra 25
SE praga <> besouro vermelho
E  folhas = Perfuradas
E  ocorrência de janeiro a abril = Sim
ENTÃO suspeita de praga = besouro vermelho CNF 50%

Regra 26
SE fumagina na face superior das folhas = Sim
ENTÃO suspeita de praga = mosca branca CNF 70%

Regra 27
SE inflorescências <> Nenhum valor encontrado
E  maturis = Rachados
OU maturis = Necrosados (muito escuros)
OU maturis = Mumificados
ENTÃO doença = antracnose CNF 100%


Regra 28
SE bolor negro na face inferior das folhas = Sim
ENTÃO doença = mofo preto CNF 100%

Regra 29
SE cinza vegetal sobre as folhas = Sim
ENTÃO doença = oidio CNF 100%

Regra 30
SE doença <> antracnose
E  folhas = Deformadas
OU folhas = Esfarelam facilmente quando amassadas
E  manchas irregulares nas folhas = Sim
ENTÃO suspeita de doença = antracnose CNF 75%

Regra 31
SE doença <> antracnose
E  folhas = Deformadas
OU folhas = Esfarelam facilmente quando amassadas
E  manchas irregulares nas folhas = Sim
E  folhas = Com exsudação de resina (goma)
ENTÃO suspeita de doença = antracnose CNF 85%

Regra 32
SE doença <> oidio
E  queda das inflorescências = Sim
ENTÃO suspeita de doença = oidio CNF 50%
