# Supplementary material for: Implementation of expert systems to support the functional evaluation of stand-to-sit activity
Source: Biomed Eng Online. 2014 Jul 21;13:98. doi: 10.1186/1475-925X-13-98 (PMC4120714; doi:10.1186/1475-925X-13-98)
Supplement: Additional file 2 — Shell Expert Sinta. [file 1475-925X-13-98-S2.zip › sintaFonte.zip!/HELP/HELPRTF.ZIP!/RESULTS.RTF]

^##^ resultados
 ^$$^ Entendendo os resultados obtidos
 ^KK^ resultados apresentados ^+^
^+^ 0700
 Ententendo os resultados apresentados

	O Expert SINTA apresenta uma janela de resultados divididas em quatro guias:

Resultados
	Aqui, é apresentada uma caixa de listagem com todos os valores encontrados para o atributo objetivoobjetivos do presente ponto da sessão de consulta, com os respectivos graus de confiançafatores. Caso o desenvolvedor tenha construído um arquivo de ajuda, o mesmo poderá ser acionado a partir dessa janela com a tecla F1.
	Os resultados aparecem em ordem decrescente de valor de confiança.

{bmc Results1.shg}

Histórico
	Quando o usuário clica nessa guia, uma árvore no formato texto é exibida com todos os passos e instanciações realizados pela máquina de inferência. É um maneira rápida de visualização geral sobre possíveis erros de transposição do raciocínio humano para a base. Esta página só está disponível livremente se o projetista da base não houver protegido a base de conhecimento.

{bmc Results2.shg}

Todos os valores
	Serão exibidos todos os valores atribuídos a todos os atributos. É uma generalização da primeira página. Exibe todos os valores de todas as variáveis. Dê um clique duplo na figura indicando a variável para exibir ou esconder todos os seus valores. Também só está disponível com permissão do projetista

{bmc Results3.shg}

O Sistema
	Exibe as todas as regras do sistema especialista. Útil também para entender como o sistema chegou àquela(s) conclusão(ões), em conjunto com o Histórico.

{bmc Results4.shg}


^##^ result_objetivo
 Aqui aparece o nome do objetivo que estava sendo procurado.

^##^ result_ajuda
 Se o sistema especialista possuir um arquivo de ajuda relacionadoinfo, esse botão estará visível. Ao clicarmos esse botão, um tópico de ajuda com maiores explicações sobre a solução selecionada na lista à direita aparecerá.

^##^ result_valores
 É uma lista com todas as soluções alcançadas para aquele determinado objetivo, em ordem decrescente de grau de confiançacnfs. Se o sistema possuir um arquivo de ajudainfo (nesses casos, aparecerá um botão azul ao lado, com um ponto de interrogação), clique duas vezes sobre um item para que maiores explicações apareçam.

^##^ result_fechar
 Fecha essa janela, continuando com a consulta se outros objetivos existirem, ou simplesmente encerrando-a.

^##^ result_expande
 Expande todos os itens da "árvore" ao lado.

^##^ result_recolhe
 Fecha todos os itens da "árvore" ao lado.

^##^ result_pesquisa
 Exibe todos os passos feitas pela máquina de inferênciaarqmaquina até a conclusão daquele determinado objetivo. Os itens com uma pequena lupa ao lado podem ser abertos ou fechados com um clique duplo.

^##^ result_todos
 Lista com todos os valores de todas as variáveisvars. Para abrir ou fechar os valores de cada variável, dê um clique duplo no pequeno fichário vermelho ao lado de cada variável.

^##^ result_sistema Aqui estão as regras que compõem o sistema especialista consultado.
