# Supplementary material for: Implementation of expert systems to support the functional evaluation of stand-to-sit activity
Source: Biomed Eng Online. 2014 Jul 21;13:98. doi: 10.1186/1475-925X-13-98 (PMC4120714; doi:10.1186/1475-925X-13-98)
Supplement: Additional file 2 — Shell Expert Sinta. [file 1475-925X-13-98-S2.zip › sintaFonte.zip!/HELP/HELPRTF.ZIP!/OPTIONS.RTF]

^##^ opcoes
 ^$$^ Opções
 ^KK^ opções
 ^++^ 60
 Opções

	Para permitir maior flexibilidade na construção de um sistema especialista, o Expert SINTA oferece diversas opções de configuração. Para acessá-las, abra o arquivogerencia da base de conhecimento desejada e, em seguida, utilize o menu Exibir|Opções. Uma caixa de diálogo com três guias aparecerá. 

Máquina de inferência
Permite pequenas modificações no comportamento da máquina de inferênciaarqmaquina, como o modo pelo qual as premissas são avaliadas.

{bmc Options1.shg}

Fatores de confiança
Após avaliar todas as premissas de uma regra, o Expert SINTA pode aceitá-la ou rejeitá-la. Se, ao calcular o fator de confiança finalcnfs ele for maior que o mínimo estipulado, a regra será aceita. É nessa janela que se define o valor mínimo. Também é aqui que se pode personalizar as funções de cálculo de fatores de confiança.

{bmc Options2.shg}

Senhas
Um sistema especialista não deixa de ser um programa de computador. Portanto, pode se fazer necessário algum tipo de proteção sobre este software. O Expert SINTA permite três níveis de proteção, exibidos na figura abaixo. Para maiores detalhes, arraste o cursor do mouse sobre a figura e clique no item desejado.

{bmc Options3.shg}


^##^ opcoes_ok
 Aperte aqui para fechar essa janela e confirmar as alterações. Eqüivale a fechar a janela utilizando o menu de sistema ou as teclas <ALT> + <F4>.

^##^ opcoes_precedencia
 Defina aqui qual a precedência dos operadores lógicos E e OU. Lembre-se da diferença: na expressão A e B ou C, se mantivermos a precedência no E, ela pode ser interpretada como (A e B) ou C. Se a precedência for do OU, A e (B ou C).

^##^ opcoes_avaliar
 Dependendo do modo como a precedência for organizada ou como os fatores de confiança forem definidos, a máquina de inferência pode saber que uma regra não vai atingir o grau mínimo de confiança para ser aceita antes mesmo de avaliá-la por completo. Se você desejar que as regras sejam sempre avaliadas completamente, mesmo quando já se sabe se serão aceitas ou não, marque esta caixa. Veja também: avaliação de premissasavaliapremissas.

^##^ opcoes_minimo
 Aqui é definido o valor de confiança mínimo para que uma regra seja aceita. Coloque na escala percentual: mínimo 0 e máximo 100.

^##^ opcoes_mudarformula
 O Expert SINTA utiliza algumas funções para calcular fatores de confiança em determinados eventos. Estes eventos podem ser uma conjunção, uma disjunção ou uma regra aceita. Pode-se usar as funções padrões do Expert SINTAcnfs ou criar novas. Cada função apresenta dois parâmetros implícitos, cnf1 e cnf2 (ou seja, o grau de confiança do primeiro termo da conjunção e o grau de confiança do segundo termo da conjunção, respectivamente). Para, por exemplo, mudar a função da conjunção para o mínimo entre os dois valores (aperte o botão Alterar se a fórmula padrão estiver sendo utilizada) ao lado da função de conjunção e digite Min(cnf1, cnf2). Para retorna à forma padrão, aperte o botão respectivo.
Veja também: usando fórmulasformulas

^##^ opcoes_mudarsenha
 Clique aqui para chamar a caixa de diálogo de definição de senhasdlgsenhas para criar ou apagar uma senhasenhas que esteja protegendo o sistema especialista.

^##^ opcoes_restricoes
 Define diferentes níveis de acesso que uma pessoa que não dispõe de senha tem direito em relação ao sistema especialista. Para maiores detalhes, clique aquisenhas.

^##^ opcoes_utilizar
 O uso de senhassenhas para proteção da base de conhecimentoarqbase só se efetivará de verdade quando esta caixa de verificação estiver marcada.

^##^ dlgsenhas
 ^$$^ Caixa de diálogo Senhas
 ^KK^ senhas, definindo
 Caixa de diálogo Senhas

	Essa caixa de diálogo é exibida a partir da janela de opções (menu Exibir|Opções). Sua função é permitir a criação de senhas para proteção da base de conhecimento. Para maiores detalhes sobre cada item desta caixa de diálogo, arraste o cursor do mouse sobre a figura abaixo e clique onde desejar.

{bmc Passwd.shg}


^##^ senha_entre
 Digite aqui a senha que irá proteger seu sistema. Note que ela não irá aparecer na tela.

^##^ senha_repita
 Repita a senha que foi digitada acima para sua maior segurança, já que não se está vendo qual a palavra-chave.

^##^ senha_limpar
 Se você não quiser mais nenhuma senha no seu sistema, clique esse botão.

^##^ senhas
 ^$$^ Como proteger seu sistema especialista
 ^KK^ senhas, como utilizá-las
 ^++^ 60
 Como proteger seu sistema especialista

	É possível associar uma senha às bases de conhecimentoarqbase criadas. Quando uma base protegida é aberta, o Expert SINTA pedirá a senha. Se for digitada corretamente, será dado nível de acesso completo àquele usuário (ele poderá modificar, visualizar, executar e imprimir a base). Também é possível não entrar com uma senha e ficar sujeito a restrições. O Expert SINTA permite três níveis de restrição:

Permitir execução e visualização
O usuário do seu sistema especialista não tem permissão para modificar a base, mas pode executá-la, depurá-la e imprimi-la. É uma opção para evitar que estranhos modifiquem a base, fazendo com que perca sua consistência.

Permitir somente execução
Um pouco mais polêmica, essa opção permite que o usuário sem senha apenas possa executar o sistema especialista, mas não pode modificá-la e menos ainda visualizá-la. Porém, um sistema especialista que não exibe o conteúdo de suas regras, não permite acompanhar sua execução e verificar como atingiu o resultado exibido não merece a mesma confiabilidade de um sistema que permite o completo acesso às suas regras. O principal motivo que levaria um projetista a escolher essa opção de restrição é o perigo de pirataria do conteúdo do sistema especialista. Mas, recomenda-se esquecer o risco e dar prioridade à confiabilidade de seu trabalho. Além disso, um sistema especialista que não é protegido por nenhuma lei de direitos autorais dá sempre a impressão de não ser tão profissional quanto deveria.

Nenhuma permissão
Somente pessoas com senha podem utilizar esse sistema especialista.


^##^ pedesenha
 Confirmação de uso
Esse sistema especialista está protegido por senha. Para podê-lo usar por completo é necessário saber a senha. Porém, geralmente o sistema proibirá apenas modificações e/ou exibição das suas regras. Experimente deixar a caixa de entrada em branco para usar o sistema com restrições.
