# Supplementary material for: Implementation of expert systems to support the functional evaluation of stand-to-sit activity
Source: Biomed Eng Online. 2014 Jul 21;13:98. doi: 10.1186/1475-925X-13-98 (PMC4120714; doi:10.1186/1475-925X-13-98)
Supplement: Additional file 2 — Shell Expert Sinta. [file 1475-925X-13-98-S2.zip › sintaFonte.zip!/HELP/HELPRTF.ZIP!/GENERAL.RTF]

^##^ o_que_e
 ^$$^ Visão geral
 ^KK^ Expert SINTA, características
 ^++^ 001
 O que é o Expert SINTA

	Sistemas especialistas são programas de computador que procuram atingir soluções em determinados problemas do mesmo modo que especialistas humanos, se estiverem sob as mesmas condições. Apesar das limitações das máquinas, é possível, hoje, a construção de sistemas especialistas com alto grau de desempenho, dependendo da complexidade de sua estrutura e do grau de abrangência desejado, sendo a arquitetura de sistemas especialistas mais comum a que envolve regras de produçãoregras (production rules). 
	Mas, como um sistema de bom tamanho tem em torno de mil regras (considerando aqueles que utilizam regras), e muito da tecnologia que o faz funcionar é encontrada em diversos sistemas, foram criadas ferramentas, shellsshell, que possuem realizam muito do trabalho necessário para transpor um sistema especialista para um computador. Essas ferramentas permitem que o criador do sistema preocupe-se somente com a representação do conhecimento do especialistas, deixando para a shell a tarefa de interpretar o conhecimento representado e executá-lo em uma máquina, além de permitir depurações e explicações de como o computador chegou àquela(s) conclusão(ões). A principal função de uma shell é simplificar ao máximo o trabalho de implementação de um sistema especialista e permitir seu uso por qualquer pessoa sem conhecimentos de informática.
	O Expert SINTA é uma ferramenta computacional (shellshell) que utiliza técnicas de Inteligência Artificial para geração automática de sistemas especialistas. Esta ferramenta utiliza um modelo de representação do conhecimento baseado em regras de produção e probabilidades, tendo como objetivo principal simplificar o trabalho de implementação de sistemas especialistas através do uso de uma máquina de inferência compartilhada, da construção automática de telas e menus, do tratamento probabilístico das regras de produção e da utilização de explicações sensíveis ao contexto da base de conhecimento modelada.
	Um sistema especialista baseado em tal tipo de modelo é bastante útil em problemas de classificação. O usuário responde a uma seqüência de menus, e o sistema encarrega-se-á de fornecer respostas que se encaixem no quadro apontado pelo usuário. Como exemplos, temos sistemas de diagnósticos médicos e configuração de redes de computadores.
	Entre outras características inerentes ao Expert SINTA, temos:

	 utilização do encadeamento para trásencadeamento (backward chaining);
	 utilização de fatores de confiançacnfs;
	 ferramentas de depuraçãoexecutando;
	 possibilidade de incluir ajudas on-line para cada basetextos.

	O Expert SINTA procura ser uma ferramenta de criação geral. Dúvidas e sugestões podem ser enviadas ao serviço de suportesuporte.

^##^ shell
 ^$$^ Shell
 ^KK^ shell
 Shell
	Um shell é uma ferramenta genérica para implementação de bases de conhecimentoarqbase. De tal modo, o desenvolvedor não se preocupa com a máquina de inferênciaarqmaquina, apenas com o conjunto de regras que modelam o conhecimento desejado.

^##^ regras
 ^$$^ Regras de produção
 ^KK^ regras de produção, definição
 Regras de produção
	Das diversas representações que existem para a criação de bases de conhecimento, as regras de realização são as mais comuns. São simplesmente um conjunto de regras no estilo SE... ENTÃO..., com a possibilidade de inclusão de conectivos lógicos relacionando os atributos no escopo da base, como vemos no exemplo a seguir:
	SE carne = vermelha E
	     cor preferida = tinto OU
	     temperatura = ambiente
	ENTÃO
	    melhor vinho = exemplo CNF 70;

^##^ encadeamento
 encadeamento para trás
	O encadeamento para trás é o modo mais comum de utilização de um Sistema Especialista. O desenvolvedor deve incluir na definição da base quais os atributos que devem ser encontrados (ou seja, os objetivos - goals - do Sistema Especialista). A máquina de inferência encarrega-se de encontrar uma atribuição para o atributo desejado nas conclusões das regras (após o ENTÃO...). Obviamente, para que a regra seja aprovada, suas premissas devem ser satisfeitas, obrigando à máquina a encontrar os atributos das premissas para que possam ser julgadas, acionando um encadeamento recursivo. Caso o atributo procurado não seja encontrado em nenhuma conclusão de regra, uma pergunta direta é feita ao usuário.

^##^ frente
 encadeamento para frente
	O encadeamento para frente consiste em definir explicitamente a ordem na qual os valores de determinados atributos serão encontrados, podendo ainda examinar todas as regras seqüencialmente. Para a máquina de inferênciaarqmaquina, não há nenhum objetivo inicial, apenas uma tentativa em concluir o máximo possível de informações que aquele conjunto de regras tem a oferecer. Por exemplo, um sistema especialista poderia apresentar um imenso formulário com todas as informações necessárias para o usuário preencher e, em seguida, consultar as regras seqüencialmente e decidir quando uma regra vale ou não de acordo com as respostas do usuário.

^##^novo
 ^$$^ O que há de novo na versão 1.1?
 ^KK^ O que há de novo na versão 1.1? ^+^
^+^ 002
 O que há de novo na versão 1.1a?

	Veja as novas características do Expert SINTA 1.1a:

	 é possível definir (na janela de edição de variáveisvars) a ordem na qual os valores aparecem em uma pergunta;
	 quando um objetivoobjetivos é alcançado, o projetista pode decidir se os resultados serão mostrados ou não. Bastante útil para simular encadeamento para frentefrente;
	 pode-se decidir se uma perguntaperguntas vai ou não permitir a entrada do grau de confiança das respostas;
	 novas opções no editor de regrascrie_regras permitem a digitação rápida e confortável de regras.
	 é possível personalizar as funções que calculam os fatores de confiançaopcoes das regras.
	 uma caixa de diálogo Dica do Dia traz informações úteis para o usuário do Expert SINTA.
	 uma nova ferramenta para avaliar as dependências entre as variáveisdepende.


^##^digitacao
 ^$$^ digitação, como acelerar
 ^KK^ Como posso acelerar a digitação de regras? ^+^
^+^ 900
 Como posso acelerar a digitação de regras?

	O ambiente do Expert SINTA permite a criação de regras sem grandes esforços. Basta que se sigam as seguintes dicas:

	 procure definir de antemão todas as variáveis antes de iniciar as regras. Mas nada impede que uma regra seja iniciada e novas variáveis sejam definidas com a regra aberta. Apenas não esqueça de fechar a janela de variáveisvars com o botão OK antes de prosseguir;
	 use as duas mãos. Enquanto uma usa o mouse para selecionar um item na listas com as variáveis e seus valores, é possível acelerar digitando a primeira ou as primeiras letras que formam o nome de uma variável ou valor. Assim, as listas irão abrir próximas ao item desejado;
	 nessa ocasião, também é possível utilizar as setas do teclado "para cima" e "para baixo" para selecionar um item de uma lista. Digite a primeira ou as primeiras letras de um nome de uma variável/valor e, em seguida, use a seta "para baixo" até aparecer o nome desejado. Mesmo em bases grandes, esse é um método mais eficaz que digitar todo o nome;
	 se você digitar a regra na ordem, ou seja, todas as premissas na ordem desejada, seguida de todas as conclusões, nem é necessário abrir mais de uma vez a janela de edição de itens. Use o botão Alternar (aquele botão verde na janela de edição de itens - NÃO na janela da regra - ao lado direito das opções de conectivo) para alternar entre premissas e conclusões;
	 use e abuse dos modelos. Eles existem para diminuir ao máximo seu trabalho. É muito comum em sistemas especialistas aparecerem regras derivadas de outras, portanto muito comum também será o uso de modelos;
	 o botão "Nova..." na janela de edição de regras não está lá apenas para ocupar espaço. Quando você terminar a digitação de regra e desejar criar uma nova, aperte esse botão para uma nova surgir e a antiga ser fechada, tudo em um passo só;


^##^simula_frente
 ^$$^ encadeamento para frente, simulando
 ^KK^ Como utilizar encadeamento para frente no Expert SINTA ^+^
^+^ 901
 Simulando encadeamento para frente

	O Expert SINTA trabalha naturalmente com encadeamento para trásencadeamento, mas é possível manipular a máquina de inferênciaarqmaquina de modo a simular encadeamento para frentefrente. Basta que as variáveis que se deseja encontrar naturalmente sejam colocadas na lista de objetivos. Obviamente, como não é desejado que uma janela de resultados apareça a cada variável encontrada, é possível desabilitar essa exibição simplesmente desmarcando a caixa "Mostrar resultados" relativo à variável desejada na janela de edição de objetivosobjetivos.


^##^conflitos
 ^$$^ quando a consulta trava
 ^KK^ Conflitos ^+^
^+^ 902
 Evitando conflitos

	Uma consulta pode travar pelo excesso de encadeamento da base de conhecimento, ou seja, quando uma determinada variável, para ser inferida, necessita do valor de outra, e esta, por sua vez, de outras, e assim por diante até esgotar os recursos da máquina. Se isso ocorrer, procure diminuir a profundidade de dependências entre variáveis reestruturando algumas de suas regras. As dependências podem ser examinadas através do menu Depurar|Dependênciasdepende.
	Porém, antes de medidas como esta, verifique se não há dependências circulares entre suas variáveis. Como assim? Vejamos o seguinte exemplo:

REGRA 30
SE    a = 1
ENTÃO b = 2

REGRA 31
SE    b = 3
ENTÃO a = 4

	Se, em determinado momento da consulta, desejássemos inferir o valor de b, poderíamos ir até à regra 30. Mas, para determinar a validade da regra 30, necessitamos do valor de a . Provavelmente a máquina de inferência iria avaliar a regra 31. Porém, como ainda não sabemos o valor de b, a máquina voltaria para a regra 30 e assim encadearia um círculo vicioso que travaria o sistema.
	É possível identificar dependências circulares facilmente com o uso da ferramenta Dependênciasdepende. Quando identificar o ponto de conflito, reestruture suas regras de modo a gerar um sistema especialista consistente.


^##^ suporte
 ^$$^ Sobre o Expert SINTA
 ^KK^ Expert SINTA, sobre ^+^
^+^ 999
 Sobre o Expert SINTA

	O Expert SINTA foi criado pelo Grupo SINTA (Sistemas INTeligentes Aplicados), um projeto UFC / UECE. A presente ferramenta foi o fruto do primeiro ano de trabalho desse grupo. Ainda em versão experimental, o SINTA espera receber várias críticas e sugestões da versão até o momento apresentado. Comentários podem ser enviados para os e-mails:
	bezerra@lia.ufc.br
	helano@lia.ufc.br
	jnando@lia.ufc.br
	rafael@fortal.uece.br

{bmc About.bmp}

Grupo SINTA - Sistemas INTeligentes Aplicados
Coordenação geral: José Helano Matos Nogueira
Integrantes:
	João Fernando Lima Alcântara
	Rafael Castro de Andrade
	Ricardo Bezerra de Andrade e Silva
Expert SINTA - idéia original de Ricardo Sousa Silvestre, ex-integrante, 1995

SECAJU - Sistema especialista em  pragas e doenças do cajueiro - é de autoria dos seguintes membros do CNPAT:
	Quélzia Maria Silva Melo  (Doutora em Entomologia)
	Antonio Lindemberg M. Mesquita (Mestre em Entomologia)
	Lianna Maria Saraiva Teixeira (Mestre em Fitossanidade)
	Francisco das Chagas O. Freire (Doutor em Fitopatologia).
SECAJU © CNPAT, 1988 - 1997

Agradecimentos especiais: 
	Genaro França - EMBRAPA 
	Marcelino Pequeno - UFC
	Tarcísio Pequeno - UFC

Expert SINTA © 1995 - 1997 Laboratório de Inteligência Artificial/LIA-UFC
Projeto financiado pela CAPES e CNPq.
