# Supplementary material for: Implementation of expert systems to support the functional evaluation of stand-to-sit activity
Source: Biomed Eng Online. 2014 Jul 21;13:98. doi: 10.1186/1475-925X-13-98 (PMC4120714; doi:10.1186/1475-925X-13-98)
Supplement: Additional file 2 — Shell Expert Sinta. [file 1475-925X-13-98-S2.zip › sintaFonte.zip!/HELP/HELPRTF.ZIP!/EXSYSTEM.RTF]

^##^ arqsinta
 ^$$^ Arquitetura de sistemas especialistas no Expert SINTA
 ^KK^ Expert SINTA, arquitetura
 ^++^ 010
 Arquitetura de sistemas especialistas no Expert SINTA

	Os sistemas especialistas gerados no Expert SINTA seguem a arquitetura abaixo (passe o mouse sobre a figura para maiores detalhes):

{bmc Struct.shg}

	O objetivo do Expert SINTA é simplificar ao máximo as etapas de criação de um SE completo. Para tanto, já oferece uma máquina de inferência básica, fundamentada no encadeamento para trásencadeamento (backward chaining).
	O encadeamento para trás destaca-se em problemas nos quais há um grande número de conclusões que podem ser atingidas, mas o número de meios pelos quais elas podem ser alcançadas não é grande (um sistema de regras de alto grau de fan out), e em problemas nos quais não se pode reunir um número aceitável de fatos antes de iniciar-se a busca por respostas. O encadeamento para trás também é mais intuitivo para o desenvolvedor, pois é fundamentada na recursão, um meio elegante e racional de programação, para onde a própria Programação em Lógica se direcionou. Em nenhum momento, porém, deixa-se de reconhecer que o encadeamento para frente (foreward chaining) possui vantagens em determinadas ocasiões. 
	A base de conhecimentos é armazenada em diversas estruturas de árvore binária. Como será visto na seção a seguir, a própria interface do programa armazena diretamente as regras e demais dados nas citadas estruturas, sem recorrer a um analisador sintático (muitas shells exigem que o usuário digite sua base em uma pseudo-linguagem, a qual é posteriormente interpretada, quando a máquina de inferência é acionada). As árvores criadas utilizam o paradigma da orientação a objetos, incluindo, principalmente, o conceito de polimorfismo, com o qual nós que representam estruturas tão diferentes como uma variável e uma cabeça de regra são tratados por um mesmo método. Como se vê, nem sempre é necessário recorrer a uma linguagem fundamentada em Lógica para criação de aplicações de Inteligência Artificial.

^##^ arqbase
 ^$$^ Base de conhecimento
 ^KK^ base de conhecimento, definição
 Base de conhecimento
É a informação que um especialista utiliza, representada computacionalmente.

^##^ arqeditor
 ^$$^ Editor de bases
 ^KK^ editor de bases
 Editor de bases
Meio pelo qual a shellshell permite a implementação das bases desejadas

^##^ arqmaquina
 ^$$^ Máquina de inferência
 ^KK^ máquina de inferência
 Máquina de inferência
Parte do sistema especialista responsável pelas deduções sobre a base de conhecimentoarqbase.

^##^ arqbanco
 ^$$^ Banco de dados global
 ^KK^ banco de dados global
 Banco de dados global
Representa as evidências apontadas pelos usuários do sistema especialista durante uma consulta.

^##^ use_regras
 ^$$^ Porque regras de produção
 ^KK^ regras de produção, detalhes
 ^++^ 011 Porque regras de produção

	As regras de produçãoregras são populares por possuírem as seguintes vantagens:

 Modularidade: cada regra, por si mesma, pode ser  considerada como uma peça de  conhecimento independente;

 Facilidade de edição (uma conseqüência da modularidade): novas regras podem ser acrescentadas e antigas podem ser modificadas com relativa independência;

 Transparência do sistema: garante maior legibilidade da base de conhecimentos.

	Portanto, é preciso ter em mente que a modularidade de um sistema baseado nessa arquitetura permite a construção passo-a-passo da base de conhecimentos, ou seja, é possível realizar vários testes com apenas um subconjunto de regras concluído. Obviamente, sabe-se que menos regras implicam geralmente em um menor número de casos abrangidos. 

^#^
^#^ regras_struct
 ^$$^ Estrutura das regras
 ^KK^ regras de produção, estrutura interna
 ^++^ 012
 Estrutura das regras no Expert SINTA

	Vejamos o seguinte exemplo ( tirado da base SECAJU, um exemplo que você irá encontrar completo junto com o Expert SINTA ):

SE	galerias nos ramos ou inflorescências
OU	inflorescências = murchas ou secas
E	brotações novas murchas
E	orifícios laterais nos ramos ou inflorescências
ENTÃO	praga = broca das pontas [90%]

	Denomina-se os conseqüentes de uma regra como as cabeças da regra e os antecedentes, caudas. Esta notação é proveniente da linguagem PROLOG, na qual as conclusões encabeçam as cláusulas.

	Para o projetista do conhecimento que cria bases utilizando o Expert SINTA, o seguinte critério para definições de assertivas deve ser seguido:

( I ). A estrutura de cada cauda (premissa) deve obedecer ao seguinte modelo: 

{bmc Tail.shg}

Arraste o cursor do mouse sobre a figura e clique para saber maiores detalhes.

( II ). A estrutura de cada cabeça (conclusão) deve obedecer ao seguinte modelo:

{bmc Head.shg}

Arraste o cursor do mouse sobre a figura e clique para saber maiores detalhes.


^##^ tail_conectivo
 conectivo é um dos seguintes elementos utilizados na lógica clássica: NÃO, E, OU. Sua função é unir a sentença ao conjunto de premissas que formam a seção de antecedentes de uma regra.

^##^ tail_atributo
 atributo é uma variávelvars capaz de assumir uma ou múltiplas instanciações no decorrer da consulta à base de conhecimentos. Cabe ao desenvolvedor definir o tipo de atributo. Um atributo é uma entidade totalmente abstrata, capaz de armazenar listas de valores cujo significado depende do contexto da base. Por exemplo, um atributo doença, no SECAJU, é capaz de ser instanciado a qualquer um ou vários elementos da lista de valores pré-definida [mofo preto, oidio, antracnose]. Números também podem ser atribuídos a variáveis;

^##^ tail_operador
 operador é um elo de ligação entre o atributo e o valor da premissa que define o tipo de comparação a ser realizada. São operadores relacionais: =, >, <=, <>, entre outros;

^##^ tail_valor
 valor é um item de uma lista a qual foi previamente criada e relacionada a um atributo. Como veremos posteriormente, a interface visual do Expert SINTA foi idealizada de modo a minimizar o trabalho do responsável pela implementação da base de conhecimentos. Para tanto, o Expert SINTA exige que os atributos sejam definidos antes de criarmos uma regra que o utilize. No momento da criação de um atributo, também é possível que seja definida uma lista de valores que constituirá o universo ao qual suas instanciações devem necessariamente pertencer. As listas criadas podem ser utilizadas para construção de menus, caso o Sistema Especialista necessite efetuar uma pergunta ao usuário referente àquele determinado atributo. Um valor também pode ser um número, caso o atributo o permita.

^##^ head_atributo
 atributo eqüivale ao mesmo atributo usado em caudastail_atributo;

^##^ head_igual
 "=" é um operador de atribuição e não de igualdade. Ou seja, o atributo, nas cabeças de regra, é sempre instanciado a um valor. Dependendo se a variável pode ou não acumular múltiplas instanciações, o novo valor substituíra o antigo ou será empilhado com os demais.

^##^ head_valor
 valor equivale ao mesmo valor utilizado em caudastail_valor;

^##^ head_cnf CNF (confidence factor, grau de confiança) é uma porcentagem indicando a confiabilidade daquela conclusão específica da regra. O grau de confiança varia de 0% a 100%.

^#^
^#^ proced_declar
 ^$$^ Conhecimento procedural x conhecimento declarativo
 ^KK^ conhecimento procedural x conhecimento declarativo
 ^++^ 013
 Conhecimento procedural x conhecimento declarativo

	Quando uma pessoa tem conhecimento de algum fato, certamente ela poderá extrair tudo o que souber sobre aquele fato quando bem entender. Porém, na Inteligência Artificial, existe um problema a mais quanto ao uso do conhecimento. 
	Supõe-se que o conhecimento por si só já é o suficiente para a resolução de problemas. Essa é a idéia por trás do conhecimento declarativo: não há preocupações quanto ao seu uso, somente quanto à sua posse e especificação, e ela já garantirá o alcance dos objetivos desejados. 
	Porém, a realidade é bem mais problemática. Um computador não é capaz de decidir qual a próxima informação que ele utilizará para o desenvolvimento de uma atividade. Faltam às máquinas um modo menos metódico e linear de ação. É necessário que especifiquemos uma estratégia de uso do seu "saber". Mas, qual é o problema, quando a máquina possui o potencial necessário? Vejamos um exemplo:

	Todos nós sabemos calcular o fatorial de um número. O fatorial de zero é um, e o fatorial dos demais números positivos é simplesmente ele multiplicado pelo fatorial do seu antecessor (como quando dizemos que o fatorial de 5 é 5 vezes o fatorial de 4). Ao colocarmos essa representação no computador, podemos representar simbolicamente por

	Fatorial (n) = n x Fatorial(n - 1)
	Ah, e é claro: Fatorial(0) = 1

	O conhecimento está aí: agora devemos ditar ao computador o meio pelo qual ele utilizará tais afirmativas. Uma estratégia bastante comum é simplesmente percorrer as informações na ordem: inicialmente, a primeira; depois, se necessário a seguinte, e assim vai. Mas, se desejarmos calcular o prosaico fatorial de 2? Para a máquina, fatorial(2) = 2 x fatorial(1). E quanto é o fatorial de 1? Nesse caso, devemos voltar à primeira informação de como se calcula o fatorial, Fatorial (n) = n x Fatorial(n - 1). Assim chegamos que fatorial(1) é 1 vezes fatorial de zero. E o fatorial de zero? Observe que, como temos um mecanismo fixo de extração de dados, não podemos avaliar a segunda informação enquanto não terminarmos a primeira. Assim, simplesmente o computador calcularia que o fatorial de zero é zero vezes o fatorial de menos um! E continuaria eternamente com essa regra.
	Esse tipo de conhecimento que depende de uma regra de extração chamamos de conhecimento procedural. Nota-se que, se simplesmente invertêssemos a ordem das informações, chegaríamos a um resultado (sempre antes de calcular um fatorial de um número, a máquina verificaria se esse número é zero)! Por isso, o uso do conhecimento é uma questão maior que simplesmente uma descrição do saber.

^##^ proced_exsinta
 ^$$^ O método de extração procedural do Expert SINTA
 ^KK^ Expert SINTA, extração de conhecimento
 ^++^ 014 O método de extração procedural de conhecimento do Expert SINTA

	O Expert SINTA utiliza o encadeamento para trás, o modo mais comum de utilização de um sistema especialista. O projetista deve incluir na definição da base quais os atributos que devem ser encontrados (ou seja, os objetivos - goals - do sistema especialista). A máquina de inferência encarrega-se de encontrar uma atribuição para o atributo desejado nas conclusões das regras (após o ENTÃO...). Obviamente, para que a regra seja aprovada, suas premissas devem ser satisfeitas, obrigando à máquina a encontrar os atributos das premissas para que possam ser julgadas, acionando um encadeamento recursivo. Caso o atributo procurado não seja encontrado em nenhuma conclusão de regra, uma pergunta direta é feita ao usuário.

^#^
^#^ encadeamento_exemplo
 ^$$^ Exemplo de encadeamento para trás
 ^KK^ encadeamento para trás, exemplo
 ^++^ 017 Exemplo de encadeamento para trás

Sejam as seguintes regras um sistema especialista para "decidir se devo ou não ir à praia amanhã".

REGRA 1
SE  	amanhã pode chover = Não
E	tenho dinheiro suficiente = Sim
E	tenho tempo suficiente = Sim
ENTÃO devo ir à praia = Sim

REGRA 2
SE  	amanhã pode chover = Sim
OU	tenho dinheiro suficiente = Não
OU	tenho tempo suficiente = Não
ENTÃO devo ir à praia = Não

REGRA 3
SE	o serviço de meteorologia disse que vai chover amanhã = Sim
ENTÃO	amanhã pode chover = Não

REGRA 4
SE	não vou sair hoje = Sim
E	nenhuma emergência ocorrer = Sim
ENTÃO	tenho dinheiro suficiente = Sim

REGRA 5
SE	minha namorada ligar = Sim
ENTÃO	não vou sair hoje = Não

REGRA 6
SE	meu patrão passar trabalho extra = Sim
ENTÃO	tenho tempo suficiente = Não

	Obs.: Observa-se que as regras acima já estão na sintaxe de regras aceita pelo Expert SINTA, o que demonstra a sua facilidade de entendimento. Os valores atribuídos não precisam ser somente Sim e Não. Para maiores detalhes sobre a criação de sistemas especialistas no Expert SINTA, consulte a visão geral sobre criação de basescriando.

	Examinando o pequeno sistema especialista acima, claramente distinguimos que o seu objetivo é determinar o valor da variável devo ir à praia. Então, iniciamos a busca por ela. O Expert SINTA procura as regras nas quais a variável sendo procurada no momento pode receber um valor se a regra for aceita (ou seja, quando ela aparece após o ENTÃO). Em seguida, a máquina de inferência verifica se a regra vale ou não. No exemplo dado, o sistema avalia a regra 1 para poder determinar se devo ir à praia ou não.
	Mas, para isso, temos que determinar se todas as premissas (amanhã pode chover = Não, tenho dinheiro suficiente = Sim, tenho tempo suficiente = Sim) são verdadeiras. Para descobrir se amanhã pode chover, tenho que repetir o processo, avaliando a regra 3. Agora tenho que saber se a meteorologia disse sobre a possibilidade de chuva amanhã. Como não existe nenhuma regra que possa concluir o que a meteorologia disse, o Expert SINTA realiza uma pergunta ao usuário, do tipo "A meteorologia afirmou se amanhã pode chover (Sim/Não)?" (claro, o computador não irá criar uma frase, essa é uma das tarefas do criador da base de conhecimento - veja o capítulo 3, Implementando o sistema, para maiores detalhes).
	Se a meteorologia disser que amanhã vai chover, então primeira premissa da regra 1 passará no teste (quem confia nos serviços meteorológicos?). Avaliando a segunda premissa e repetindo o processo do encadeamento para trás, chegamos à regra 4. Mas, para descobrir se eu vou sair hoje ou não, é preciso recorrer à regra 5 (observa-se a forte idéia de recursão no backward chaining). A regra 5 leva a uma pergunta, pela qual concluiremos se tal regra vai ser aprovada. Se realmente for aprovada, a primeira premissa da regra 4 é verdadeira, fazendo com que verifiquemos agora a segunda premissa. Quando todas as premissas da primeira regra forem avaliadas, podemos determinar se a regra 1 foi aprovada ou não. Caso contrário, passamos para a próxima regra que possa concluir um valor para a variável devo ir à praia.

^#^
^#^ vars_unimulti
 ^$$^ Variáveis univaloradas e multivaloradas
 ^KK^ variáveis, a diferença entre multivaloradas e univaloradas
 ^++^ 016 Variáveis univaloradas e multivaloradas

	Outra questão deve ser levada em conta durante o acompanhamento da execução de um sistema especialista. Uma única variável pode receber vários valores em uma única consulta ao sistema. É muito comum em sistemas de diagnóstico médico, onde o paciente pode apresentar mais de uma doença. Portanto, é importante saber lidar com variáveis que podem ter apenas uma instanciação (univalorada) ou múltiplas (multivaloradas). Para saber como indicar ao Expert SINTA quais são as variáveis multivaloradas e univaloradas, clique aquivars.
	Quando a máquina de inferênciaarqmaquina está atrás de encontrar instanciações para uma variável univalorada, ela irá procurar até encontrar um valor ou até esgotar todas as possibilidades da base de conhecimentoarqbase. Se, por algum motivo, durante a busca de uma outra variável, uma variável univalorada receber um valor quando já possuía outro, esse valor antigo será descartado e o novo vigorará.
	A busca de valores para variáveis multivaloradas prossegue até que toda a base de conhecimento seja explorada. Os valores permanecem acumulados. É nessa hora que é preciso ter cuidado com contradições presentes na base. O Expert SINTA, na presente versão, não faz verificações de inconsistências lógicas.
	Obs.: variáveis numéricas são tratadas como univaloradas, sempre.

^#^
^#^avaliapremissas
 ^$$^ Operadores e o cálculo de premissas
 ^KK^ operadores
 ^++^ 015 Operadores e a avaliação de premissas

	O Expert SINTA, salvo seja especificado o contrárioopcoes, procurará aceitar ou rejeitar um regra analisando o mínimo de premissas possível. Por exemplo:

SE
   PREMISSA 1
E  PREMISSA 2
ENTÃO
   CONCLUSÃO 1

	Nesse caso, se a premissa 1 for falsa, a premissa 2 não será avaliada. Do modo inverso, temos o exemplo a seguir:

SE
   PREMISSA 1
OU PREMISSA 2
ENTÃO
   CONCLUSÃO 1

	Sendo verdadeira a premissa 1, a premissa 2 não será avaliada e a regra, aprovada.


^#^
^#^monotonicidade
 ^$$^ Leitura adicional: conhecimento monotônico x conhecimento não-monotônico
 ^KK^ monotonicidade
 ^++^ 016 Leitura adicional: conhecimento monotônico x conhecimento não-monotônico

	Digamos que você estacionou o seu carro em frente ao local de trabalho. Evidentemente, ao retornar, você saberá o local onde deixou o seu carro e saberá que o utilizará para voltar para casa. Mas, em um intervalo de trabalho, alguém lhe diz que seu carro foi roubado! Agora você sabe que não o encontrará onde o tinha deixado.
	No dia-a-dia, enfrentamos situações que modificam as nossas certezas. O que antes tínhamos por certo agora estamos convictos que não são mais realidade. Isso porque nós pensamos em um tipo de lógica não-monotônica, ou seja, podemos receber informações que contrarie as que já possuímos.
	No tratamento tradicional de informações da Inteligência Artificial, o conhecimento monotônico, ou seja, aquele que ao acrescentarmos novas informações nunca fica contraditório, inconsistente, é o comumente usado. O Expert SINTA trata o conhecimento de forma essencialmente monotônica.
