# Supplementary material for: Implementation of expert systems to support the functional evaluation of stand-to-sit activity
Source: Biomed Eng Online. 2014 Jul 21;13:98. doi: 10.1186/1475-925X-13-98 (PMC4120714; doi:10.1186/1475-925X-13-98)
Supplement: Additional file 2 — Shell Expert Sinta. [file 1475-925X-13-98-S2.zip › sintaFonte.zip!/HELP/HELPRTF.ZIP!/EXECUTE.RTF]

^##^ executegeral
 ^$$^ Visão geral de consultas
 ^KK^ consultas, visão geral
 ^++^ 089
 Visão geral de uma consulta

	O Expert SINTA mantém uma interface uniforme para a consulta de qualquer base de conhecimento criada em seu ambiente. Existem dois modos pelos quais podem ser efetuadas consultas: 

 a execução usual, na qual o usuário acompanha uma seqüência de menus de múltipla (ou única) escolha, nos quais deve-se indicar informações que resultarão nas conclusões atingidas pelo sistema especialista;

 o modo de acompanhamento, pelo qual é possível examinar o conteúdo das regras que formam o sistema, bem como acompanhar a execução passo a passo e as instâncias (valores) que cada variável possui em determinado momento. Para utilizar este modo, é preciso que o projetista da base não tenha protegido o acesso às regras por meio de uma senhasenhas.

	Um sistema especialista procura atingir conclusões para determinados objetivos. Sempre que um desses objetivos é atingido, ou quando se esgotam todas as possibilidades, o Expert SINTA apresentará uma janela com os resultados e o acompanhamento de como se chegou àquela conclusão (também é necessário que o projetista não tenha restringido o acesso).
	Alguns sistemas também devem trazer um sistema de ajuda on-linetextos, pelo qual serão dadas maiores informações de como se deve melhor utilizar as respostas conseguidas, bem como explicações mais detalhadas sobre as funções daquele determinado sistema especialista.
	Existem dois modos pelos quais se pode acompanhar uma consulta no Expert SINTA. Utilizando o menu da janela principal ou a barra de ferramentasbarra. As operações de execução/depuração podem ser:

 iniciar a consulta;
 interromper a execução para saber que regra está utilizando naquele ponto;
 executar passo a passo;
 adicionar elementos da base (variáveis) para acompanhar os valores que eles recebem durante a consulta;
 ver esses valores escolhidos;
 abandonar a consulta.


^##^ visualizar
 ^$$^ Visualizando regras
 ^KK^ regras, visualizando
 ^++^ 090
 Visualizando regras

	Na caixa de diálogo de visualização de regras (acionada a partir do botão Visualizar, na janela da base de conhecimentos), estão disponíveis dois modos de visualização: 

Todas as regras
	Assinale essa opção para ver todas as regras em ordem de criação.

Regras que contenham as seguintes variáveis na conclusão
	Como o próprio nome diz, acrescente variáveis à lista logo através dos botões localizados entre as listas. Somente regras que contenham tais variáveis em suas conclusões aparecerão.

	Para maiores detalhes, arraste o ponteiro do mouse sobre a figura abaixo e clique quando desejar.

{bmc View.shg}


^##^ ver_todasregras
 Selecionando essa opção, serão exibidas todas as regras.

^##^ ver_conclusoes
 Selecionando essa opção, deve-se escolher abaixo variáveis de modo que somente as regras que contiverem essas variáveis em suas conclusões (após a cláusula ENTÃO) serão exibidas.

^##^ ver_variaveis
 Nessa lista estão todas as variáveis do sistema especialista. Pode-se escolher algumas para que somente as regras que as contiverem em suas respectivas conclusões (após a cláusula ENTÃO) serão exibidas. Também é necessária que a opção acima esteja marcada (Regras que contenham as seguintes variáveis...).

^##^ ver_adiciona
 Clique aqui para adicionar a variável selecionada à esquerda para a lista das variáveis que determinam as regras a serem exibidas.

^##^ ver_todas
 Clique aqui para adicionar todas as variáveis para a lista das variáveis que determinam quais regras serão exibidas.

^##^ ver_elimina
 Clique aqui para retirar a variável selecionada na lista à direita da lista das variáveis que determinam quais regras serão exibidas.

^##^ ver_nenhuma
 Clique aqui para limpar a lista à direita.

^##^ ver_exibicao
 Se a opção Regras que contenham as seguintes variáveis na conclusão estiver marcada, somente serão exibidas as regras que contiverem alguma variável presente nessa lista nas suas conclusões (após a cláusula ENTÃO).

^##^ ver_ok
 Clique aqui para exibir as regras de acordo com as opções escolhidas.

^##^ ver_cancelar
 Clique aqui para fechar essa janela sem exibir as regras.

^##^ executando
 ^$$^ Uma consulta típica
 ^KK^ consulta ^+^
^+^ 091
 Uma consulta típica

	Iniciando-se a consulta, seja no modo de execução ou acompanhamento, o sistema especialista geralmente apresentará uma tela de abertura, com informações sobre seu funcionamento, os autores e casos aos quais ele se aplica. Leia atentamente esse texto de abertura, pois poderá trazer informações úteis. Se o botão de ajuda estiver disponível (aquele situado no canto inferior direito), aperte-o se desejar informações mais detalhadas.
	Para iniciar a consulta, pressione o botão OK. O botão Cancelar abandona a execução do sistema especialista. O botão Ajuda chama a ajuda da basetextos, se ela estiver disponível.

{bmc Overture.shg}

	A consulta se desenvolve por meio de menus de múltipla (ou única escolha). Um menu típico é mostrado na figura abaixo. Arraste o cursor do mouse sobre a figura para maiores detalhes.

{bmc Running.shg}

	A próxima figura mostra um menu para entrada de valores numéricos.

{bmc Running2.shg}

	Escolha a(s) opção(ões) desejadas marcando as respectivas caixas de verificação, localizadas sempre  à esquerda de cada alternativa. Observe, à direita, a possibilidade de entrar com o grau de confiança da sua resposta. Graus de confiança são utilizados quando não se possui certeza absoluta sobre um fato. Assim sendo, você pode expressar sua dúvida por meio de um número percentual, ou seja, a sua resposta terá validade no intervalo de zero a cem por cento. Cada resposta assinalada pode ter o seu próprio grau de confiança.
	Caso realmente não saiba responder àquela pergunta, simplesmente deixe todas as alternativas em branco. O Expert SINTA entenderá que você não sabe como responder ao questionamento apresentado.
	No caso de valores numéricos, se for necessário usar o separador decimal, use o ponto ou a vírgula dependendo da configuração de idioma de seu computador.
	Não esqueça que, a qualquer momento, é possível mudar o modo de execução da base ou interrompê-la usando o menu ou a barra de ferramentasbarra.

^##^ run_pergunta
 Aqui aparecerá uma perguntaperguntas que deve ser respondida utilizando-se as opções abaixo.

^##^ run_alt
 Uma pequena informação surgirá aqui, indicando se é possível marcar mais de uma alternativa das opções abaixo.

^##^ run_itens
 Aqui estão as opções que devem ser marcadas para responder a pergunta em questão. Em alguns casos, é possível assinalar mais de um item. Caso não saiba o que responder, simplesmente não assinale nenhuma. O sistema interpretará sua resposta como sendo algo desconhecidodesconhecido. Para saber como criar essas opções, clique aquivars.

^##^ run_cnf
 Entre aqui a confiançacnfs que cada resposta respectiva merece, em porcentagem (até 100%). Obviamente, é necessário que o item ao lado esteja marcado para que se possa entrar algum dado aqui.

^##^ run_ok
 Após a entrada de sua resposta, clique aqui para confirmá-la e prosseguir na consulta. Para cancelar uma consulta, utilize o menu Consulta|Finalizar ou a combinação de teclas <ALT>+F4.

^##^ run_porque
 Algumas perguntas trazem explicaçõesperguntas sobre a sua importância. Se elas estiverem disponíveis, é possível clicar nesse botão para visualizá-las. O Expert SINTA gera uma explicação caso o criador da base não tenha definido nenhuma. Essa explicação exibe a importância daquela pergunta para a conclusão de algum fato. Ela não irá aparecer se o sistema não disponibilizar a exibição das regrassenhas.

^##^ run_altnum
 Se você não souber realmente informar a resposta, deixe a caixa de texto abaixo em branco. Para maiores informações sobre valores desconhecidos, clique aquidesconhecido.

^##^ run_valor
 Entre aqui um valor numérico que seja uma resposta aceitável para a pergunta acima. Para saber mais sobre variáveis numéricas, clique aquivars.

^##^ acompanha
 ^$$^ A janela de acompanhamento
 ^KK^ depuração
 ^KK^ janela de acompanhamento
 ^++^ 092
 A janela de acompanhamento

	Quando se está no modo de acompanhamento, uma nova janela sempre estará aberta, o depurador.

{bmc Debugger.shg}

	O depurador exibe, em sua caixa de listagem, todas as regras da base de conhecimento. A sentença em destaque é aquela que está sendo analisada no momento pela máquina de inferência. Observe que é possível rolar a lista para cima e para baixo, exibindo o resto da base, mas somente a linha representando o ponto de parada pode ser selecionada.
	Utilize o menu Depurar|Passo, ou o botão respectivo da barra de ferramentas, ou ainda a tecla F8 para continuar a acompanhar, passo a passo, a execução do sistema. Se o sistema estiver realizando uma pergunta para você, primeiro responda antes de executar um passo, ou nada acontecerá.
	Fechar o depurador eqüivale a voltar ao modo de execução. Se você deseja abandonar a consulta utilize o menu Consulta|Finalizar ou o botão respectivo na barra de ferramentasbarra.

^##^ vars_ver
 ^$$^ Acompanhando variáveis
 ^KK^ variáveis, depurando
 ^KK^ janela de acompanhamento
 ^++^ 093
 Acompanhando variáveis

	Para melhor depuração da base de conhecimentos, é possível monitorar o valor de cada variável existente. Para isso, utilize a opção Depurar|Adicionar Variáveis... e selecione as variáveis desejadas para monitoração (é possível selecionar várias ao mesmo tempo). Arraste o cursor do mouse sobre a figura abaixo para aprender maiores detalhes sobre cada item dessa caixa de diálogo.

{bmc AddVars.shg}

	Para visualizar o(s) valor(es) das variáveis, selecione o menu Depurar|Ver Variáveis durante uma consulta. Clique em qualquer item da lista e o(s) seu(s) respectivo(s) valor(es) aparecerão. Arraste o cursor do mouse sobre a figura abaixo para aprender maiores detalhes sobre cada item dessa caixa de diálogo.

{bmc WatVars.shg}

Observação 1: obviamente, se a base não estiver sendo executada, de nada servirá abrir a janela de visualização;
Observação 2: para apagar um item da lista de visualização, selecione-o e pressione a tecla DELETE;
Observação 3: note que somente as variáveis ainda não incluídas aparecem na caixa de diálogo Adicionar Variáveis...

^##^ adiciona_vars
 Selecione uma ou mais variáveis dessa lista para acompanhar durante a execução. Mantenha a tecla CTRL pressionada enquanto clica para selecionar várias.

^##^ adiciona_ok
 Pressionando esse botão, as variáveis selecionadas acima serão adicionadas à lista de variáveis em acompanhamento.

^##^ adiciona_cancelar
 Clique nesse botão para fechar essa janela sem adicionar nenhuma variável extra.

^##^ debug_vars
 Clique sobre qualquer item dessa lista para exibir os respectivos valores aos quais a variável foi instanciada.

^##^ debug_valores
 Aqui são exibidas as instâncias (valores) da variável selecionada acima. Se a base de conhecimento não estiver sendo executada, uma mensagem de erros aparecerá aqui. Se a variável não houver sido instanciada naquele momento, nada aparecerá nessa caixa.

^##^ debug_fechar
 Fecha a janela de acompanhamento de variáveis.

^##^ guia
 ^$$^ Guia rápido de execução
 ^KK^ consulta, barra de ferramentas ^+^
^+^ 094
 Guia rápido de execução

	Essa seção tem como objetivo mostrar rapidamente os comandos de execução e depuração na barra de ferramentasbarra e menus do Expert SINTA.

	Para iniciar a execução de uma base de conhecimentos, selecione Iniciar no menu Consulta, ou aperte F9.
	Eqüivale ao botão  na barra de ferramentas. 

	A qualquer momento a execução pode ser interrompida para que se observe a base de conhecimentos e o ponto no qual a máquina de inferência está posicionada. Para isso, selecione Interromper a consulta, no menu Consulta, ou aperte F5. Com isso, pode ser feita a execução passo a passo.
	Eqüivale ao botão  na barra de ferramentas.

	No modo de execução passo a passo, a máquina de inferência só reage quando o usuário aciona um passo. Para acionar um passo, selecione Passo no menu Depurar, ou aperte F8. Também é possível iniciar uma sessão de consulta a partir da inferência passo a passo.
	Eqüivale ao botão  na barra de ferramentas.

	Para abortar uma consulta, selecione Finalizar no menu Consulta.
	Eqüivale ao botão  na barra de ferramentas.

	Para voltar a uma pergunta anterior e modificar sua resposta, selecione Voltar pergunta no menu Consulta.
	Eqüivale ao botão  na barra de ferramentas.

	Para a exibição das variáveis instanciadas até o momentovars_ver, selecione Ver no menu Depurar.
	Eqüivale ao botão  na barra de ferramentas.
