# Supplementary material for: Implementation of expert systems to support the functional evaluation of stand-to-sit activity
Source: Biomed Eng Online. 2014 Jul 21;13:98. doi: 10.1186/1475-925X-13-98 (PMC4120714; doi:10.1186/1475-925X-13-98)
Supplement: Additional file 2 — Shell Expert Sinta. [file 1475-925X-13-98-S2.zip › sintaFonte.zip!/HELP/HELPRTF.ZIP!/CONTENTS.RTF]

^##^ contents
Expert SINTA

O que é o Expert SINTAo_que_e
A finalidade do Expert SINTA e suas
facilidades.

Criando uma base de conhecimentoscriando
Os passos necessários para que um especialista humano 
possa modelar o seu conhecimento e fazê-lo funcionar
em uma máquina.

Executando e depurando uma base de conhecimentosexecutando
Como detectar erros através do acompanhamento passo a passo
da máquina de inferência do Expert SINTA e colocar a base de
conhecimentos em funcionamento.

Entendendo o modo como se realizam os cálculos de fatores de confiançacnfs
O porquê de utilizar fatores de confiança e a descrição das fórmulas utilizadas
pelo Expert SINTA.

Criando textos explicativos para a sua base de conhecimentostextos
Um modo de tornar a sua base mais didática e útil para os usuários.

Entendendo os resultados apresentadosresultados
Como usufruir das informações que o Expert SINTA
calcula para você.

Serviço de suportesuporte
Tire suas dúvidas e 
apresente sugestões.
