# Supplementary material for: Implementation of expert systems to support the functional evaluation of stand-to-sit activity
Source: Biomed Eng Online. 2014 Jul 21;13:98. doi: 10.1186/1475-925X-13-98 (PMC4120714; doi:10.1186/1475-925X-13-98)
Supplement: Additional file 2 — Shell Expert Sinta. [file 1475-925X-13-98-S2.zip › sintaFonte.zip!/HELP/HELPRTF.ZIP!/CNFS.RTF]

^##^ fatores
 ^$$^ O que são fatores de confiança
 ^KK^ fatores de confiança, definição ^+^
^+^ 016
 O que são fatores de confiança

	Sabemos que o conhecimento humano não é determinístico. Não há especialista que sempre se encontre em condições de afirmar determinada conclusão com certeza absoluta. Graus de confiança são freqüentemente atribuídos às suas respostas,  principalmente quando existe mais de uma. Este, sem dúvida, é um dos mais fortes pontos críticos na elaboração de uma representação computacional do saber humano. 
	Vejamos a dificuldade em representar a confiabilidade das informações:

 Especialistas humanos não se sentem confortáveis em pensar em termos de probabilidade. Suas estimativas não precisam corresponder àquelas definidas matematicamente;

 Tratamentos rigorosamente matemáticos de probabilidade utilizam informações nem sempre disponíveis ou simplificações que não são claramente justificáveis em aplicações práticas.

	Na verdade, existem duas correntes de pensamento: aquela que utiliza fórmulas estatísticas rigorosas e aquela que utiliza uma abordagem ad hoc sobre os fatores de certeza, ou seja, mais generalizada e sem uma base matemática forte. O Expert SINTA utiliza atualmente uma abordagem não-estatística, mas baseada na Teoria dos Conjuntos.

^##^ cnfs
 ^$$^ Como se calculam os fatores de confiança no Expert SINTA
 ^KK^ fatores de confiança, como se aplica no Expert SINTA ^+^
^+^ 017
 Os fatores de confiança no Expert SINTA

	Vejamos como se atribuem fatores de confiançafatores na presente versão do Expert SINTA:

Caso 1prob_caso1: Quando queremos saber o valor final atribuído às variáveis na conclusão de um regra.
Caso 2prob_caso2: Cálculo do grau de confiança com o operador E.	
Caso 3prob_caso3: Cálculo do grau de confiança com o operador OU.
Caso 4prob_caso4: Quando uma variável recebe duas vezes o mesmo valor em pontos diferentes da consulta.

	Obs._1_: O sistema admite 50% como valor mínimo de confiança para que uma igualdade seja considerada verdadeira, mas esse valor pode ser mudado. O intervalo de grau de confiança varia de 0 a 100.
	Obs._2_: O operador E tem precedência sobre o OU.


^##^ prob_caso1
 Seja c_1_ o grau de confiança atribuído ao resultado final da premissa de uma regra r. Na conclusão de r, devemos ter expressões como var = value CNF c_2_, onde var é uma variável, value é um termo qualquer que pode ser atribuído a uma variável, c_2_ é um real pertencente ao intervalo [0; 100] que representa o grau de confiança da atribuição. Mas, c_2_ é apenas uma referência, pois o valor final é dependente do resultado da premissa. Assim sendo, realizar-se-á a operação var = value CNF c_1_. c_2_.

Exemplo de aplicação:

SE fumagina = sim
ENTÃO suspeita de praga = mosca branca, 
	grau de confiança (CNF) 70%.

Assim sendo, supondo que o grau de confiança da igualdade fumagina = sim é 80%, teremos que à variável suspeita de praga será atribuído o valor mosca branca, com o respectivo grau de confiança 0.80 * 0.70 = 0.56 = 56%.


^##^ prob_caso2
 Se possuímos duas igualdades var_1_ = value_1_ e var_2_ = value_2_, com os respectivos graus de confiança c_1_ e c_2_, temos que a sentença var_1_ = value_1_ E var_2_ = value_2_ retornará como valor de confiança c_1_. c_2_.

Exemplo de aplicação:

SE estados das folhas = esfarelam facilmente 
E   presença de manchas irregulares = sim...

	Se o grau de confiança da igualdade estados das folhas = esfarelam facilmente é 80% e o grau de confiança da igualdade presença de manchas irregulares = sim é 70%, temos que a conjunção das duas sentenças retornará um valor CNF de 56%, pois esse é o produto dos dois valores.

^##^ prob_caso3
 Se possuímos duas igualdades var_1_ = value_1_ e var_2_ = value_2_, com os respectivos graus de confiança c_1_ e c_2_, temos que a sentença var_1_ = value_1_ OU var_2_ = value_2_ retornará como valor de confiança c_1_ + c_2_ - c_1_ . c_2_.

Exemplo de aplicação:

SE  besouros vermelhos = sim 
OU larvas marrons = sim ...	

	Se o grau de confiança da igualdade besouros vermelhos = sim é 80% e o grau de confiança da igualdade larvas marrons = sim é 70%, temos que a disjunção das duas sentenças retornará um valor CNF de 0.70 + 0.80 - 0.70 * 0.80 = 1.50 - 0.56 = 0.94 = 94%.


^##^ prob_caso4
 Em momentos diferentes de uma consulta, uma mesma variável var pode receber o mesmo valor v, sendo que até à penúltima instanciação ela possuía grau de confiança c_1_, e a última atribuiu um CNF c_2_. Sendo assim, temos que o valor final de confiança para var = v será dado pela fórmula c_a_ + c_n_ - c_a_ * c_n_, onde c_a_ representa o grau de confiança antes da última mudança e c_n_ o último grau de confiança atribuído.

Exemplo de aplicação:

	A variável doença possuía valor mofo preto com grau de confiança 60%. Após a aplicação de outras regras chegou-se a uma outra atribuição doença = mofo preto, desta vez com CNF 50%.
	O cálculo se dá de maneira semelhante à aplicação da regra OU: doença terá como um dos valores mofo preto, com respectivo grau de confiança 0.60 + 0.50 - 0.60 * 0.50 = 1.10 - 0.30 = 0.80 = 80%.


^##^ desconhecido
 ^$$^ O valor DESCONHECIDO
 ^KK^ desconhecido ^+^
^+^ 018 Lidando com o desconhecido

	Vejamos o seguinte exemplo.

	REGRA 1
SE  	amanhã pode chover = Não
E	tenho dinheiro suficiente = Sim
E	tenho tempo suficiente = Sim
ENTÃO devo ir à praia = Sim

REGRA 2
SE  	amanhã pode chover = Sim
OU	tenho dinheiro suficiente = Não
OU	tenho tempo suficiente = Não
ENTÃO devo ir à praia = Não

REGRA 3
SE	o serviço de meteorologia disse que vai chover amanhã = Sim
ENTÃO	amanhã pode chover = Não

REGRA 4
SE	não vou sair hoje = Sim
E	nenhuma emergência ocorrer = Sim
ENTÃO	tenho dinheiro suficiente = Sim

REGRA 5
SE	minha namorada ligar = Sim
ENTÃO	não vou sair hoje = Não

REGRA 6
SE	meu patrão passar trabalho extra = Sim
ENTÃO	tenho tempo suficiente = Não

Se respondermos que o serviço de meteorologia disse que amanhã VAI chover, então a regra 3 irá falhar. Mas, observe que isso NÃO quer dizer que a variável amanhã pode chover irá receber o valor "Sim". Muito pelo contrário, essa variável não vai ser instanciada, permanecendo sem instanciações. Portanto, a regra 2 também vai falhar, e o sistema simplesmente responderá que não conseguiu atingir nenhuma conclusão. Quando se projeta um sistema especialista, deve-se levar em conta o maior número de possibilidades possível, para evitar mensagens desagradáveis como "Nenhum valor encontrado..."
	Obs.: O Expert SINTA não apresenta a opção SENÃO, mas futuras versões poderão apresentá-la.
Além do uso de graus de confiança para tratamento de incertezas, e da "não-instanciação" de variáveis, o Expert SINTA permite o uso de um valor especial, passível de ser usado por todas as variáveis. É o DESCONHECIDO, que representa uma indeterminação total sobre as instanciações de uma variável. Este valor pode ser tanto incluído nas regras como dado como resposta pelo usuário.
