# Supplementary material for: Implementation of expert systems to support the functional evaluation of stand-to-sit activity
Source: Biomed Eng Online. 2014 Jul 21;13:98. doi: 10.1186/1475-925X-13-98 (PMC4120714; doi:10.1186/1475-925X-13-98)
Supplement: Additional file 2 — Shell Expert Sinta. [file 1475-925X-13-98-S2.zip › sintaFonte.zip!/HELP/HELPRTF.ZIP!/BASE.RTF]

^##^ criando
 ^$$^ Bases de conhecimento no Expert SINTA
 ^KK^ base de conhecimento, como se usa no Expert SINTA ^+^
^+^ 050
 Visão geral de uma base de conhecimento no Expert SINTA

	O Expert SINTA foi criado de modo a permitir ao próprio analista de conhecimentos implementar a base desejado. O usuário do Expert SINTA não necessita do menor conhecimento de programação, apenas de como interagir em ambientes visuais.
	Na gerência de uma base de conhecimentosgerencia, deve-se fornecer ao sistema as seguintes informações:

	 Variáveisvars
	 Regrascrie_regras
	 Perguntasperguntas
	 Objetivosobjetivos
	 Informações adicionaisinfo

	Todos esses elementos podem ser controlados pela janela KIBkib. Ao término de tais definições, é possível a execução a base.
	Obs1.: O Expert SINTA, até a presente versão, não é capaz de identificar erros de inconsistência lógica. Por isso, erros (como "estouro de pilha", no caso em que a base entra em recursão infinita) podem vir a ocorrer em bases mal implementadas.
	Obs2.: É necessário que se possua ao menos uma variável objetivoobjetivos definida. Informações adicionaisinfo são dispensáveis.


^##^ gerencia
 ^$$^ Gerenciando de uma base de conhecimentos
 ^KK^ base de conhecimento, gerenciando ^+^
^+^ 051
 Gerenciando bases de conhecimento

	Como padrão, O Expert SINTA grava as bases de conhecimento geradas em arquivos *.BCM. Para abrir bases pré-existentes em seus discos, utilize o menu Arquivo|Abrirarquivoabrir. Consulte a ajuda do Windows se precisar de maiores informações de como trabalhar com a caixa de diálogo Abrir Arquivo. Para criar uma base totalmente nova, use o menu Arquivo|Novoarquivonovo. Imediatamente, uma nova base de conhecimento, vazia, será criada para você. 
	Finalmente, quando desejar salvar uma base na qual se está trabalhando para posterior uso, utiliza-se o menu Arquivo|Salvararquivosalvar. Quando você estiver salvando a base pela primeira vez, uma caixa de diálogo aparecerá para que seja indicado o nome do arquivo na qual a base será gravada e a pasta na qual ele será encontrada. Consulte a ajuda do Windows se precisar de maiores explicações sobre o uso da caixa de diálogo Salvar Arquivo.
	A impressão se dá através do menu Arquivo|Imprimirarquivoimprimir. Uma caixa de diálogo de configuração de impressoras aparecerá. Consulte a ajuda para detalhes de como usar a caixa de diálogo de configuração de impressoras.
	Muitas das opções dos menus encontram-se também na barra de ferramentas, inicialmente presente no topo da área de trabalho do Expert SINTA. É possível mudar a posição da barra de ferramentas clicando sobre ela e arrastando a moldura que surge para a posição desejada. Clique nos diferentes botões abaixo para saber a quais menus são referenciados por quais botões.

{bmc ToolBox.shg}


^##^ arquivonovo
 Arquivo|Novo
Essa opção, a primeira do menu Arquivo, permite a criação de uma nova base de conhecimento vazia. O nome da base e o local onde ela é gravada são definidos somente na gravação do arquivoarquivosalvar. Sempre que se desejar criar uma base do começo, utilize essa opção. 

^##^ arquivoabrir
 Arquivo|Abrir
Quando se desejar utilizar um sistema especialista previamente criado, deve-se informar onde o mesmo se encontra no seu computador. Para isso, utilize o menu Abrir, o segundo do menu Arquivo. Uma caixa de diálogo aparecerá, onde será necessário que se informe qual o arquivo contendo uma base de conhecimento se deseja abrir.

^##^ arquivosalvar
 Arquivo|Salvar
Sempre que se desejar armazenar uma base de conhecimento para posterior uso, é necessário que a mesma seja salva em disco. Para isso, utilize o menu Salvar (localizado sob o menu Arquivo). Se esta for a primeira vez que o sistema é salvo, uma caixa de diálogo aparecerá, e será necessário que se indique o nome da base e o local onde ela será gravada. Bases já anteriormente salvas são gravadas automaticamente. Para salvar uma base com outro nome, utilize o menu Salvar Como, localizado imediatamente abaixo do menu Salvar.

^##^ executarcomecar
 Consulta|Iniciar
Inicia uma consulta ao sistema especialistaexecutando desenvolvido no Expert SINTA.

^##^ executarinterromper
 Consulta|Interromper a consulta
Paralisa uma consulta ao sistema especialistaexecutando, permitindo que se veja em que ponto da base de conhecimento a máquina de inferência se situa.

^##^ executarparar
 Consulta|Finalizar
Aborta uma consulta ao sistema especialistaexecutando antes do seu término.

^##^ executarvoltar
 Consulta|Voltar pergunta
Volta para a última pergunta realizada pelo sistema em uma consultaexecutando, permitindo modificar uma resposta.

^##^ depurarpasso
 Depurar|Passo
Executa passo a passo uma consultaexecutando que estiver paralisada.

^##^ depurarver
 Depurar|Ver variáveis
Exibe as variáveis que estão sendo acompanhadasvars_ver durante uma consulta.

^##^ depuraradicionar
 Depurar|Adicionar variáveis...
Adiciona variáveis a uma lista de acompanhamentovars_ver.

^##^ arquivoimprimir
 Arquivo|Imprimir
Imprime a base de conhecimento, com todas as regras, variáveis, objetivos e perguntas. Em alguns casos pode ser mais vantajoso salvar o sistema em um arquivo texto e depois, utilizando um editor de textos, fazer as alterações desejadas. Para isso, utilize o menu Arquivo|Exportar...

^##^ ajuda
 ?|Conteúdo
Exibe a presente ajuda.

^##^ sistemanovaregra
 Sistema|Nova regra...
Cria uma nova regranovaregra para o sistema especialista.

^##^ sistemaabreregra
 Sistema|Abrir regra
Abre uma regracrie_regras selecionada na janela KIBkib.

^##^ sistemadeleta
 Sistema|Exclui regra
Exclui uma regracrie_regras selecionada na janela KIBkib.

^##^ sistemaver
 Sistema|Visualizar
Exibe todas as regrasvisualizar do sistema especialista.

^##^ sistemavars
 Sistema|Variáveis
Abre a janela de edição de variáveisvars.

^##^ sistemaobjetivos
 Sistema|Objetivos
Define os objetivosobjetivos do sistema especialista.

^##^ sistemainterface
 SistemaIInterface
Define a interfaceperguntas do sistema especialista com o usuário final.

^##^ sistemainfo
 Sistema|Informações
Permite a inclusão de informações complementaresinfo sobre o sistema especialista.

^##^ barra
 ^$$^ A barra de ferramentas
 ^KK^ barra de ferramentas
 A barra de ferramentas

	A barra de ferramentas permite acionar diversas opções do menu de modo mais direto. Arraste o cursor do mouse sobre a figura abaixo para saber mais sobre cada botão.

{bmc ToolBox.shg}

^##^ kib
 ^$$^ A janela KIB
 ^KK^ kib, janela ^+^
^+^ 052
 A janela Knowledge In a Box (KIB)

{bmc kib.shg}

	Essa janela traz todas as opções de edição de base, além de uma lista com todas as regras presentes até o momento. Tais opções, e outras, encontram-se repetidas na barra de ferramentas, inicialmente presente no topo da área de trabalho do Expert SINTA.

{bmc ToolBox.shg}


^##^ kibnovaregra
 Clique aqui para criar uma nova regra. Veja também: gerenciando regrascrie_regras.

^##^ kibabrirregra
 Abre a regra que está selecionada na lista ao lado. Veja também: gerenciando regrascrie_regras.

^##^ kibexcluirregra
 Exclui a regra que está selecionada na lista ao lado. Veja também: gerenciando regrascrie_regras.

^##^ kibvisualizar
 Exibevisualizar todas as regras em conjunto.

^##^ kibvariaveis
 Chama a janela de edição de variáveisvars.

^##^ kibobjetivos
 Define os objetivosobjetivos da base de conhecimento.

^##^ kibinterface
 Chama a janela de edição de interfaceperguntas.

^##^ kibinformacoes
 Permite a inclusão de informações extrasinfo sobre a base.

^##^ kibfechar
 Fecha a base de conhecimento. Somente uma pode estar aberta por vez.

^##^ kibmover
 Lista de regras: um duplo-clique abre a regra. É possível arrastar o nome da regra até a posição desejada, para alterar a ordem na qual elas aparecem. Veja também: gerenciando regrascrie_regras.

^##^ vars
 ^$$^ Como criar variáveis e valores
 ^KK^ variáveis, criando
 ^KK^ valores, criando ^+^
^+^ 053
 Criando variáveis e valores

	Antes de podermos criar regras, é necessário que todas as variáveis utilizadas, bem como seus respectivos valores, sejam criados. Através desse mecanismo, a base fica organizada, fácil de manter e as regrascrie_regras podem ser criadas visualmente. A janela de edição de variáveis é exibida abaixo. Para saber mais sobre cada item da janela, arraste o mouse sobre o ponto desejado e clique.

{bmc Vars.shg}

	Vejamos os passos necessários para criação e edição de variáveis e valores:

 Na janela KIBkib, clique o botão Variáveis;

 A janela de variáveis é aberta, e duas caixas de listagem podem ser vistas. A caixa da esquerda representa as variáveis já existentes, enquanto à da direita, os valores relativos à variável que está atualmente selecionada (clique em determinado item da lista de variáveis para exibir os valores correntes);

 Adicionar variáveis é simples: basta que pressione o botão Adicionar Variável e, em seguida, digitemos um valor na caixa de texto Variável. Atenção: Para confirmarmos o novo valor é necessário que tecle <ENTER> imediatamente após a digitação ou cliquemos o pequeno botão à direita (o que possui a marca de verificação). Para cancelar a inserção, é só clicar em qualquer outro controle da janela (incluindo os botões OK e Cancelar, os quais fecham a janela).

 Adicionar valores é um procedimento análogo, só é necessário que uma variável esteja selecionada;

 Para indicar se uma determinada variável é numérica, multivaloradamulti ou univaloradauni, primeiro selecionamos uma variável e, em seguida, o tipo desejado entre os botões de opção localizados no canto inferior direito da janela

 Variáveis numéricas não podem ter valores pré-definidos. Nesse caso, a lista de valores passa atuar como lista de intervalo, ou seja, nela é exibido o intervalo de valores aceitáveis para aquela variável. Intervalos são incluídos de maneiras idênticas a valores, mas é necessário obedecer a certas regras:
	- Os intervalos devem ser da forma a;b ou ;b ou a;, onde a e b representam números reais . Assim sendo 
	a;b representa um número entre a e b;
	;b um número maior ou igual a b;
	a; um número menor ou igual a a;
	- Somente um intervalo pode ser incluído por variável numérica;

	Para definir a ordem na qual os valores aparecerão nas janelas criadas durante uma pergunta, clique na variável desejada, em seguida clique e segure o cursor sobre o valor desejado e arraste-o até a posição certa.
	Observação: Mudar de variável não numérica para numérica pode implicar perda de valores.
	Observação 2: apagar uma variável ou valor encontrado em uma regra ou mais regras implica que as mesmas também devem ser eliminadas. O Expert SINTA efetua uma confirmação antes de eliminá-las.

^##^ multi
 variável multivalorada
Uma variável é dita multivalorada quando pode assumir mais de um valor. Se durante a consulta um novo valor é inferido a tal variável, ele é acumulado junto com os anteriores. Isso é muito comum em variáveis objetivoobjetivos, pois diversas soluções podem ser alcançadas para um determinado quadro de consulta.

^##^ uni
 variável univalorada
Uma variável é dita univalorada quando pode assumir somente um valor. Se durante a consulta um novo valor é inferido a tal variável, o valor antigo é descartado.

^##^ lista_vars
 Todas as variáveis já incluídas aparecerão aqui.

^##^ lista_valores
 Nessa caixa aparecerão os valores relacionados à variável selecionada na lista à esquerda.

^##^ nome_var
 Sempre que uma variável for selecionada na lista acima, seu nome aparecerá aqui. Utilize essa caixa de texto para indicar um nome para uma variável já existente ou criar uma nova variável.

^##^ nome_valor
 Sempre que um valor for selecionado, seu nome aparecerá aqui. Utilize essa caixa de texto para indicar um nome para um valor já existente ou criar um novo valor.

^##^ botao_v
 Clique aqui para efetuar uma modificação realizada na caixa de texto à esquerda, tanto para modificar uma variável/valor existente para incluir novos. Possui o mesmo efeito que apertar a tecla <ENTER>. Não confundir com o botão OKOK_vars abaixo.

^##^ botao_x
 Clique aqui para desfazer quaisquer modificações na caixa de texto à esquerda. Possui o mesmo que apertar a tecla <ESC>. Não confundir com o botão CancelarCancelar_vars abaixo.

^##^ incluir_vars
 Aperte esse botão sempre antes de incluir uma variável. A caixa de texto Variável será limpa e o cursor nela aparecerá, indicando que o nome da nova variável deve ser digitado. Após incluir uma nova variável, o cursor irá para a caixa de texto relativo a valores. Você não é obrigado a incluir valores para aquela nova variável, embora essa ação seja comum quando da sua criação.

^##^ excluir_vars
 Aperte esse botão para excluir a variável que estiver selecionada na lista de variáveis. Se nenhuma estiver marcada, o botão estará em cinza, indicando que a ação não é possível. Importante: se alguma regra estiver utilizando essa variável, ela será apagada. O Expert SINTA lhe avisá se isso poder ocorrer e pedirá sua confirmação.

^##^ incluir_valores
 Aperte esse botão sempre antes de incluir um valor. A caixa de texto Valor será limpa e o cursor nela aparecerá, indicando que o nome da nova variável deve ser digitado. Na realidade, ao se incluir uma nova variável, o Expert SINTA imediatamente entra em um modo de inserção de valores. Você só precisará utilizar essa opção se quiser associar um valor a uma variável tempos depois de criá-la.

^##^ excluir_valores
 Aperte esse botão para excluir o valor que estiver selecionada na lista de variáveis. Se nenhum estiver marcada, o botão estará em cinza, indicando que a ação não é possível. Importante: se alguma regra estiver utilizando esse valor, ela será apagada. O Expert SINTA lhe avisá se isso poder ocorrer e pedirá sua confirmação.

^##^ botao_numerica
 Marque esse botão se a variável referente for numérica.

^##^ botao_multi
 Marque esse botão se a variável referente for multivaloradamulti.

^##^ botao_uni
 Marque esse botão se a variável referente for univaloradauni.

^##^ OK_vars
 Pressione OK para fechar essa janela e confirmar as alterações feitas, não para efetuar uma única alteração. Para isso, use o botão pequeno marcado com um "V" verdebotao_v. Eqüivale a fechar a janela com o botão de sistema do Windows ou com <CTRL> + F4.

^##^ Cancelar_vars
 Pressione Cancelar para fechar essa janela sem confirmar as alterações feitas, não para desfazer uma única alteração. Para isso, use o botão pequeno marcado com um "X" vermelhobotao_x.

^##^ novaregra
 ^$$^ Caixa de diálogo "Nova regra"
 ^KK^ regras, novas
 Nova Regra

	Essa caixa de diálogo surge sempre antes da criação de uma nova regra. Ela tem como utilidade inicializar uma regra nova com alguma informações, como a sua posição em relação às outras (a ordem das regras tem importânciaproced_declar), bem como um "modelo" no qual ela pode se basear.
	Arraste o mouse sobre a figura e clique para maiores informações:

{bmc NewRule.shg}


^##^ novaregra_modelo
 Se a regra que você está criando é muito parecida com outra, é possível reaproveitá-la. Indique aqui uma regra e todos os seus itens serão copiados para a nova regra criada, poupando o seu trabalho. Para criar um regra do zero, deixe marcado o item NENHUM.

^##^ novaregra_ordem
 Indique a posição da regra (lembre-se que, em conhecimento procedural, a ordem das regras é importante). Se desejar, mude sua posição depois.

^##^ novaregra_ok
 Clique aqui para iniciar a edição de uma nova regra.

^##^ novaregra_cancelar Clique aqui para cancelar a edição da regra.

^#^
^#^ crie_regras
 ^$$^ Como criar regras
 ^KK^ regras, criando e modificando ^+^
^+^ 054
 Trabalhando com regras

	O Expert SINTA utiliza regras de produçãoregras para modelar o conhecimento humano, o que o torna ideal para problemas de seleção, no qual uma determinada solução deve ser atingida a partir de um conjunto de seleções.
	Para abrir uma regra, selecione-a na janela KIBkib e pressione o botão Abrir.
	Para excluir uma regra, selecione-a na janela KIB e pressione o botão Excluir.
	Para criar uma nova regra, selecione-a na janela KIB e pressione o botão Nova Regra. Para maiores detalhes sobre a criação de novas regras, clique aquinovaregra.

	Veja abaixo um exemplo de uma janela contendo uma regra. Para saber mais sobre cada item, arraste o mouse sobre a figura e clique onde quiser.

{bmc Rule.shg}

Para editar uma regra, acompanhe o seguinte esquema:

	 Para inserir uma premissa, marque o local na caixa de listagem na qual se desejar inserir a sentença e clique o botão Inserir. Uma outra janela aparecerá com várias listas.
	
	{bmc RuleItem.shg}
	
 Para inserir uma conclusão, proceda de modo análogo, escolhendo um item a partir do conectivo ENTÃO na sua regra. Em conclusões, somente o operador de atribuição ("=") pode ser utilizado. A última lista presente indica o grau de confiança daquela atribuição. Deixar a lista vazia indica uma confiança de cem por cento.

	{bmc RuleIte2.shg}

 Para editar uma sentença, selecione-a e clique o botão Alterar (ou dê um duplo clique sobre ela). Para eliminar uma sentença, selecione-a e clique o botão Excluir.

 Se a variável relativa à sentença for uma variável numérica, a caixa de valores não exibirá uma lista, apenas um espaço vazio para a entrada de um valor numérico ou uma fórmulaformulas.

{bmc RuleIte3.shg}

	Observação: também é possível visualizarvisualizar regras em conjunto.

^##^ regra_itens
 É aqui que aparecem as sentenças da sua regra. A sentença selecionada será aquela que sofrerá alterações. Ao se inserir um novo item, o mesmo aparecerá imediatamente após a sentença selecionada.

^##^ regra_nome
 Crie um nome para regra, a fim de facilitar a sua localização e esclarecer sua importância.

^##^ regra_ordem
 Indica a posição da regra em relação às demais. Também pode ser mudada arrastando o nome da regra para a posição desejada na janela KIBkib.

^##^ regra_altera
 Edita o item selecionado na lista acima.

^##^ regra_insere
 Insere um novo item de regra imediatamente após ao item selecionado na lista acima.

^##^ regra_deleta
 Apaga a sentença selecionada na lista acima.

^##^ regra_OK
 Clique aqui para fecha a regra e confirmar as alterações. É necessário que a regra possua pelo menos uma premissa e uma conclusão.

^##^ regra_cancela
 Fecha a regra sem levar em consideração as modificações.

^##^item_nova
 Clicando aqui, uma nova regra será criada, fechando-se a atual.

^##^ item_var
 Indica qual a variávelvars que será comparada a um valor nesse item de regra.

^##^ item_operador
 Identifica o tipo de relação: igualdade ("="), diferença ("<>"), outros (">", ">=", "<", "<=" - somente para variáveis numéricas).
^##^ item_valor
 Indica qual o valorvars que será comparada a uma variável nesse item de regra.

^##^ item_conectivo
 Caso a premissa não seja a primeira da regra, você deverá indicar o tipo de conectivo (E ou OU, conjunção ou disjunção) pelo qual a premissa se liga à anterior. Também é possível avaliar esse item por sua negação, marcando a caixa Não.

^##^ item_alterar
 Aperte aqui para confirmar a mudança do item. Esta janela será fechada.

^##^ item_inserir
 Aperte aqui para inserir uma nova premissa ou conclusão. Você poderá inserir várias sem necessidade de fechar esta janela, contanto que as premissas/conclusões inseridas sejam consecutivas. 

^##^ item_cancelar
 Aperte aqui para cancelar a mudança do item.
^##^ item_fechar
 Aperte aqui quando terminar de inserir os itens desejados.

^##^ item_alternar
 Se você estiver inserindo uma premissa e desejar agora inserir uma conclusão, aperte aqui. As novas conclusões inseridas aparecerão ao final da regra. No caso contrário, se você houver iniciado a inserção de uma conclusão e agora desejar uma premissa, aperte aqui. As novas premissas aparecerão ao final das já existentes.

^##^ item_var_2
 Indica qual a variávelvars que receberá um valor nesse item de regra.
^##^ item_igual
 Atenção: o símbolo de igual está indicando aqui uma atribuição e não uma comparação.

^##^ item_valor_2
 Indica qual o valorvars que será atribuído a uma variável nesse item de regra.

^##^ item_cnf
 Indica o grau de confiança (CNF) dessa regra em relação a uma determinada sentença, em porcentagem. Deixar a lista em branco eqüivale a 100 por cento de confiança.

^##^ item_numero
 Entre aqui um número ou uma fórmula matemáticaformulas.

^##^ item_zoom
 Clique aqui para exibir o conteúdo da caixa ao lado em uma janela maior.

^##^ formulas
 ^$$^ Inserindo fórmulas matemáticas em uma regra
 ^KK^ fórmulas matemáticas
 ^KK^ funções matemáticas
 ^++^ 055
 Inserindo fórmulas matemáticas em uma regra

	Quando se utiliza variáveis numéricas, é comum que queiramos atribuir a elas valores extraídos de fórmulas matemáticas, ou compará-las a valores equivalentes. Se você já sabe como criar regrasregras, já deve saber como inserir uma sentença de regra contendo uma variável numérica e um valor. Para inserir uma função ou expressão, simplesmente digite-a no lugar reservado ao valor. Deve-se, porém, seguir as seguintes regras:

 Os operadores matemáticos válidos são: + (adição), - (subtração), * (multiplicação), / (divisão), + (sinal unário indicando que um número é positivo), - (sinal unário indicando que um número é negativo);

 As seguinte funções podem ser utilizadas:
- Min(a, b): retorna o menor valor entre a e b;
- Max(a, b): retorna o maior valor entre a e b;
- Sqr(x): raiz quadrada de x;
- Exp(x): exponencial de x;
- Pot(a, b): retorna a elevado a b;
- Log(a, b): logaritmo de a na base b;
- Ln(x): logaritmo natural de x;
- Abs(x): módulo (valor absoluto) de x;
- Sen(x): seno de x, x em radianos;
- Cos(x): cosseno de x, x em radianos;
- Tan(x): tangente de x, x em radianos;

 Variáveis podem ser utilizadas nas expressões. Para atribuir a raiz de uma variável a outra, pode-se fazer algo do tipo a = sqr(b). A diferença entre maiúsculas e minúsculas não é considerada. Atenção: se o nome da variável contiver espaços em branco ou símbolos, coloque-a na expressão entre aspas. Por exemplo: Min("juros de dezembro", "juros de janeiro");

 Se o valor da expressão for desconhecidodesconhecido, uma variável que esteja recebendo o valor retornado também será instanciada para desconhecido;

 É possível utilizar parênteses e aninhar funções.

 ATENÇÃO! Use como separador decimal o ponto, nunca a vírgula!


^##^ perguntas
 ^$$^ Personalizando perguntas
 ^KK^ perguntas, personalizando
 ^++^ 055
 Personalizando interface

	Na versão atual, o Expert SINTA não apresenta grande flexibilidade no desenho da interface do sistema especialista com o usuário final. O Expert SINTA gera automaticamente telas com perguntas dirigidas ao usuário baseado nas regras e variáveis. 
	Além disso, embora não seja necessário, é bastante útil que as perguntas que o sistema realiza ao usuário tenham um texto claro. Sempre que o Expert SINTA procura o valor de uma variávelvars v e não encontra uma regra capaz de inferir um valor, ele realiza uma pergunta direta ao usuário. Caso o desenvolvedor tenha criado um texto para a pergunta relativa àquela variável, ele aparecerá. Caso contrário, uma pergunta genérica e pouco esclarecedora aparecerá: "Qual o valor de v?".
	Portanto, vale a pena que o desenvolvedor facilite a vida daqueles que utilizarão seu sistema. Além disso, é possível incluir os motivos pelos quais aquela pergunta é necessária, deixando ainda mais claro quais os objetivos do sistema.
	Para criar perguntas, realizemos os seguintes passos:

 Na janela da base de conhecimentos, clique o botão Perguntas;

 Na parte superior direita da janela de Perguntas, encontra-se uma lista das variáveis que já possuem perguntas prontas. Para visualizá-las, clique em alguma. Para modificar a pergunta ou o motivo, simplesmente rescreva o conteúdo das caixas de texto. As mudanças são automáticas. Para eliminar uma pergunta, simplesmente mova o item selecionado para a lista da esquerda, pressionando o botão correspondente;

 Na lista superior esquerda, aparecem as variáveis que ainda não possuem nenhuma pergunta pronta. Para criar uma nova pergunta, selecione a variável desejada e mova-o para a lista da direita, pressionando o botão correspondente, e digite o conteúdo das caixa de texto. As mudanças são automáticas.

	Arraste o mouse sobre a figura abaixo para conhecer os itens da janela de definição de interface e clique quando desejar.

{bmc Intface.shg}

	Observação: Não faz sentido criar perguntas para variáveis que podem ser inferidas a partir das conclusões de uma regra. O Expert SINTA ignorará esse tipo de pergunta.
	Observação 2: É possível selecionar várias variáveis ao mesmo tempo. Use a tecla <CONTROL> ou a tecla <SHIFT> simultaneamente ao ato de clicar sobre o item.

^##^ intface_restantes
 Nessa lista estão todas as variáveis que ainda não possuem nenhuma pergunta a ela associada.

^##^ intface_adiciona
 Clicando aqui, a variável que estiver selecionada à esquerda irá agora fazer parte da lista de variáveis que possui uma pergunta relacionada.

^##^ intface_elimina
 Apaga a pergunta relacionada à variável selecionada à direita, devolvendo-a à lista da esquerda.

^##^ intface_adicionadas
 Nessa lista se encontram todas as variáveis que possuem alguma pergunta relacionada. A pergunta (e o motivo) correspondentes sempre aparecem nas caixas abaixo.

^##^ intface_pergunta
 Sempre um item é selecionado na lista de variáveis com perguntas, essa caixa de texto é relacionada a essa pergunta. Para modificar alguma pergunta, basta reescrever o seu conteúdo. A atualização é automática.

^##^ intface_motivo
 Sempre um item é selecionado na lista de variáveis com perguntas, essa caixa de texto é relacionada ao motivo correspondente àquela pergunta. Para modificar algum motivo, basta reescrever o seu conteúdo. A atualização é automática. Para inserir uma quebra de linha, simplesmente aperte <ENTER> quando o cursor estiver na posição desejada.

^##^ intface_ok
 Aperte aqui para fechar essa janela e confirmar as alterações. Eqüivale a fechar a janela utilizando o menu de sistema ou as teclas <CTRL> + <F4>.

^##^ intface_cancelar
 Aperte aqui para fechar essa janela sem confirmar as alterações. Todas as modificações feitas desde a última vez que a janela foi aberta serão perdidas.

^##^ use_cnf
 Com essa opção marcada, o usuário poderá, durante a pergunta, indicar o grau de confiança de suas respostas.

^##^ objetivos
 ^$$^ Definindo os objetivos
 ^KK^ objetivos, definindo
 ^++^ 056
 Definindo os objetivos

	O objetivo de uma consulta a um especialista é encontrar a resposta para um determinado problema. Assim também o é um Sistema Especialista. A diferença é que, aqui, os "problemas" são representados por variáveisvars. Antes de podermos executar nosso sistema pela primeira vez, é preciso que definamos quais são as variáveis (chamadas variáveis objetivo) que irão controlar o modo como a máquina de inferência se comporta.
	Sigamos o seguinte esquema para determinação de objetivos:

 Na janela de base de conhecimentos, cliquemos o botão Objetivos. Aparecerá uma janela com duas listas: a lista das variáveis comuns e a das variáveis objetivo;

 Para fazer de uma variável comum uma variável objetivo e vice-versa, selecione e variável desejada e clique no botão correspondente, aquele que aponta para a lista de destino. A lista com uma seta dupla (seja para a esquerda ou para direita) move todos os items de uma lista para outra. Para mudar a ordem dos elementos da lista de objetivos, clique em um item e arraste-o até a posição desejada.

	Arraste o mouse sobre a figura abaixo para maiores detalhes sobre cada item da janela.

{bmc Targets.shg}

	Observação: caso o sistema seja posto em funcionamento sem nenhum objetivo definido, nada acontecerá (seria como falar com um especialista sem ter nada para descobrir!)

^##^ objetivos_mostrar
 Se essa caixa estiver marcada, o Expert SINTA mostrará uma janela com os resultados do objetivo sempre que todas as conclusões sobre ele tiverem sido efetuadas.
^##^ objetivos_vars
 Lista todas as variáveis que não são variáveis objetivos.

^##^ objetivos_var2
 Lista todas as variáveis que são objetivos. Importante: a máquina de inferênciaarqmaquina realizará a busca na ordem na qual os objetivos aqui aparecem. Para mudar a ordem, simplesmente clique e segure o cursor do mouse sobre um dos itens desta lista e arraste-o (o cursor mudará) até a nova posição desejada.

^##^ objetivos_adiciona
 Transforma a variável que está selecionada na lista à esquerda em uma variável objetivo.

^##^ objetivos_todos
 Transforma todas as variáveis criadas até o momento em variáveis objetivo do sistema especialista.

^##^ objetivos_elimina
 Retira a variável seleciona à direita da lista de variáveis objetivo.
^##^ objetivos_nenhum
 Limpa a lista de variáveis objetivo.

^##^ textos
 ^$$^ Criando textos explicativos para sua base de conhecimentos
 ^KK^ textos explicativos ^+^
^+^ 057
 Criando textos explicativos

	Um bom Sistema Especialista não deve saber somente encontrar respostas, mas também fazer o usuário utilizar a resposta com fins práticos. Para isso, o Expert SINTA possibilita a inclusão de tópicos de ajuda para os valores possíveis de determinados atributos, associando a um arquivo no formato Ajuda do Windows a sua base. Explicações sobre a criação de arquivos de ajuda fogem ao escopo do presente documento, mas existem diversas documentações fornecidas por terceiros. O Expert SINTA também não distribui o compilador necessário para a criação do arquivo final.
	A idéia desses textos explicativos não é somente ativar uma ajuda on-line quando o usuário estiver utilizando o sistema, mas ir diretamente ao tópico explicativo de uma resposta, quando o Expert SINTA atinge um determinado objetivo. Para isso, o desenvolvedor deve, na janela "Informações" ativada a partir da janela de base de conhecimentos, escolher os valores das variáveis-objetivo que irão possuir um número de contexto de ajuda. Quando o usuário final ativar a ajuda on-line, ela aparecerá diretamente no tópico referente à solução específica. O desenvolvedor também deve incluir o nome do arquivo de ajuda na caixa de texto nome do arquivo de ajuda, na mesma janela.
	Por exemplo, no caso da configuração de redes de computadores, o protocolo ideal sugerido pelo sistema foi o TCP/IP. Então, bastará ao usuário selecionar esse item e acionar a ajuda que imediatamente aparecerão informações sobre tal protocolo. 
	Maiores informações sobre números de contexto de ajuda e compiladores de Windows Help  (como o HC31) podem ser conseguidas através de terceiros. Para saber mais sobre a implementação de contextos de ajuda no Expert SINTA, clique aquiinfo.


^##^ info
 ^$$^ Adicionando informações sobre a base
 ^KK^ informações adicionais
 ^++^ 058
 Adicionando informações sobre a base

	Após a implementação do sistema, nada mais natural que desejemos incluir vários tipos de informação complementar, como, por exemplo, o nome da base, os autores, um texto de abertura e textos didáticos relativos às soluções encontradas.
	Na janela de Informações (acionada através do botão Informações da janela da base de conhecimentos), todos esses dados podem ser incluídos. A janela é dividida em duas guias:

Geral
	Aqui, informações iniciais sobre o sistema são incluídas, tais como o nome da base, os autores e um texto de abertura. Todo os dados aparecerão em uma tela de abertura quando a base é posta em funcionamento. Para permitir a exibição dos dados, deixe marcada a caixa de verificação Exibir tela de abertura.

{bmc Info1.shg}

Arquivo de Ajuda
	Para garantir melhor aproveitamento da sua base, textos didáticostextos podem ser relacionados a cada solução encontrada. Para tanto, crie um arquivo de ajuda no formato Ajuda do Windows e defina os contextos de ajuda relativos a cada valor (se desejado) de cada variável objetivo.

{bmc Info2.shg}

^##^ info_nomebase
 Digite aqui o nome do sistema especialista para identificação.

^##^ info_autores
 Digite aqui os nomes dos criadores da base de conhecimento.

^##^ info_abertura
 Digite aqui um resumo informativo sobre o sistema especialista, que pode ser, inclusive, exibido no início de uma consulta. Dica: esse campo pode ser usado para exibir também a licença de uso do sistema.

^##^ info_exibir
 Deixe a caixa marcada caso queira que cada consulta comece com uma tela de abertura exibindo as informações aqui digitadas.

^##^ info_nomeajuda
 Entre com o nome do arquivo de ajuda que contiver informações extras sobre a base. Obviamente, ele não é necessário, mas aumenta em muito a utilidade do sistema especialista.

^##^ info_vars
 Lista todas as variáveis objetivo. A idéia é incluir explicações sobre as conclusões. Como as conclusões se relacionam principalmente a variáveis objetivo, não seria útil a criação de contextos para as demais variáveis. Ao selecionar um item nessa caixa, seus respectivos valores possíveis aparecerão na lista ao lado.

^##^ info_valores
 Lista todos os valores possíveis da variável selecionada na lista à esquerda.

^##^ info_contexto
 Entre aqui o número do contexto de ajuda daquele determinado valor selecionado na lista à esquerda. Para maiores detalhes sobre arquivos de ajuda, consulte documentação de terceiros. Para eliminar um contexto de ajuda, digite 0 nessa caixa.

^##^ info_v
 Clique aqui para confirmar mudanças no contexto de ajuda em edição. Eqüivale a pressionar a tecla <ENTER>. Não confundir com o botão OK, o qual fecha a janela.

^##^ info_x
 Clique aqui para desfazer mudanças no contexto de ajuda em edição. Eqüivale a pressionar a tecla <ESC>. Não confundir com o botão Cancelar, o qual fecha a janela.

^##^ depende
 ^$$^ Dependências entre variáveis
 ^KK^ variáveis, dependências
 ^++^ 059
 Dependências entre variáveis

	Como se sabe, para que a máquina de inferênciaarqmaquina defina o valor de uma variável, esta deve ser inferida pelo encadeamento para trásencadeamento embutido no Expert SINTA. Isto implica que uma variável, para ser determinada, necessita das instâncias (valores) de outras variáveis, ou de indicação direta do usuário.
	Com o crescimento da base de conhecimento, o número de variáveis pode aumentar de forma a dificultar sua gerência. Para melhor organização, é sempre bom que se saiba de que modo as variáveis estão unidas, seja para verificar conflitosconflitos, seja para verificar redundâncias.
	A ferramenta Dependências do Expert SINTA auxilia nesta tarefa. Clicando-se no menu Depurar|Dependências, a seguinte janela aparecerá:

{bmc Depend.shg}

	Observe que os pontos de partida da "árvore" exibida acima são os objetivos do sistema, enquanto os nós terminais são sempre requisições ao usuário final. Se conflitosconflitos ocorrerem, será exibida uma mensagem de erro.

^##^ depend_vars
 Este painel exibe as dependências de forma hierarquizada. As variáveis trazem incluídas dentro de seu domínio todos os seus pré-requisitos: outras variáveis ou a indicação do usuário. Se houver uma dependência circular entre variáveis, o conflito será indicado.

^##^ depend_fechar
 Fecha esta caixa de diálogo.

^##^ depend_expande
 Expande todos os itens da "árvore" acima.

^##^ depend_recolhe
 Fecha todos os itens da "árvore" acima.
