# Supplementary material for: Implementation of expert systems to support the functional evaluation of stand-to-sit activity
Source: Biomed Eng Online. 2014 Jul 21;13:98. doi: 10.1186/1475-925X-13-98 (PMC4120714; doi:10.1186/1475-925X-13-98)
Supplement: Additional file 2 — Shell Expert Sinta. [file 1475-925X-13-98-S2.zip › sintaFonte.zip!/HELP/SECAJU.ZIP!/TOPICOS3.RTF]

^##^ sus_traca
 ^$$^ traça das castanhas
 ^KK^pragas, suspeitas
 ^++^ 025
 Traça das castanhas (suspeita)
Veja também:recom_trac_sus

Para um diagnóstico categórico, verifique se as castanhas estão broqueadas, com a amêndoa totalmente destruída.

^##^ sus_broca
 ^$$^ broca das pontas
 ^KK^ pragas, suspeitas
 ^++^ 026
 Broca das pontas (suspeita)
Veja também:recom_broc_sus

Para um diagnostico categórico, verifique se existem galerias dentro dos ramos ou inflorescências
OU se, conjuntamente, as inflorescências estão murchas ou secas, as brotações novas estão murchas ou secas, e existem orifícios laterais nos ramos ou inflorescências.

^##^ sus_pulgao
 ^$$^ pulgão das inflorescências
 ^KK^ pragas, suspeitas
 ^++^ 027
 Pulgão das inflorescências (suspeita)
Veja também:recom_pulg_sus

Para um diagnóstico categórico, verifique se existem insetos pequenos, moles, de cor amarelo-clara ou verde-escura, nas inflorescências, folhas (principalmente na face inferior) e maturis.

^##^ sus_eriofiideo
 ^$$^ eriofiídeo das flores
 ^KK^ pragas, suspeitas
 ^++^ 028
 Eriofiídeo das flores (suspeita)
Veja também:recom_erio_sus

Para um diagnóstico categórico, verifique se existem colônias de ácaros de corpo estreito e longo, nas inflorescências. Eles só são visualizados com o auxílio de uma lupa.

^##^ sus_tripes
 ^$$^ tripes
 ^KK^ pragas, suspeitas
 ^++^ 029
 Tripes (suspeita)
Veja também:recom_tripes_sus

Para um diagnóstico categórico, verifique se existem colônias de insetos pequenos, amarelados, com uma cinta avermelhada, na face inferior das folhas.

^##^ sus_saiajusta
 ^$$^ lagarta saia justa
 ^KK^ pragas, suspeitas
 ^++^ 030
 Lagarta saia justa (suspeita)
Veja também:recom_saia_sus

Para um diagnóstico categórico, verifique se existem lagartas de tamanho médio (6 cm), coloração cinza, lisas (sem pêlos), com a cabeça e as partes laterais do corpo pretas com manchas brancas,
OU se existem lagartas enroladas em folhas (normalmente secas), de modo a lembrar um cartucho ou uma saia justa.
OUTRA evidência certa da presença da lagarta saia justa é a formação de espiraizinhas de ovos presas, por uma extremidade, aos ramos.

^##^ sus_verde
 ^$$^ lagarta verde
 ^KK^ pragas, suspeitas
 ^++^ 031
 Lagarta verde (suspeita)
Veja também:recom_verde_sus

Para um diagnóstico categórico, verifique se existem lagartas de tamanho grande (10 cm), coloração variável (verde-alaranjada, amarela ou marrom), e pêlos urticantes.

^##^ sus_folhas
 ^$$^ lagarta das folhas
 ^KK^ pragas, suspeitas
 ^++^ 032
 Lagarta das folhas (suspeita)
Veja também:recom_folhas_sus

Para um diagnostico categórico, verifique se existem lagartas de tamanho grande (8 cm), coloração verde, e pêlos curtos, avermelhados e urticantes.

^##^ sus_noiva
 ^$$^ lagarta véu de noiva
 ^KK^ pragas, suspeitas
 ^++^ 033
 Lagarta véu de noiva (suspeita)
Veja também:recom_noiva_sus

Para um diagnóstico categórico, verifique se existem lagartas de tamanho pequeno (3 cm), coloração verde-clara, e pêlos longos e verdes.

^##^ sus_besouro
 ^$$^ besouro vermelho
 ^KK^ pragas, suspeitas
 ^++^ 034
 Besouro vermelho (suspeita)
Veja também:recom_besouro_sus

Para um diagnóstico categórico, verifique se existem besourinhos de cor vermelha
OU larvas de cor marrom-clara, movimentos lentos, e tamanho em torno de 2 cm. Elas costumam descer para o chão, onde ficam enterradas sob a forma de pupa.

^##^ sus_mosca
 ^$$^ mosca branca
 ^KK^ pragas, suspeitas
 ^++^ 035
 Mosca branca (suspeita)
Veja também:recom_mosca_sus

Para um diagnóstico categórico, verifique se existe uma secreção pulverulenta branca, na face inferior das folhas, 
OU se existem insetos parecidos com uma mosca, de cor branca.

^##^ sus_larva
 ^$$^ larva do broto terminal
 ^KK^ pragas, suspeitas
 ^++^ 036
 Larva do broto terminal (suspeita)
Veja também:recom_broto_sus

Para um diagnóstico categórico, verifique se as folhas do broto terminal estão repolhadas, isto é, fortemente retorcidas e enroladas umas sobre as outras.

^##^ sus_cigarrinha
 ^$$^ cigarrinha
 ^KK^ pragas, suspeitas
 ^++^ 037
 Cigarrinha (suspeita)
Veja também:recom_cig_sus

Para um diagnóstico categórico, verifique se existe uma espuma sobre as inflorescências, maturis (frutos novos) ou pedúnculos.

^##^ sus_antracnose
 ^$$^ antracnose
 ^KK^ doenças, suspeitas
 ^++^ 038
 Antracnose (suspeita)
Veja também:recom_antrac_sus

Para um diagnóstico categórico, verifique se as inflorescências estão murchas, secas ou com lesões necróticas escuras, 
E se os maturis estão rachados, mumificados ou necrosados (muito escuros).

^##^ sus_mofo
 ^$$^ mofo preto
 ^KK^ doenças, suspeitas
 ^++^ 039
 Mofo preto (suspeita)
Veja também:recom_mofo_sus

Para um diagnóstico categórico, verifique se existem colônias de fungo em forma de um bolor negro feltroso, na face inferior das folhas.

^##^ sus_oidio
 ^$$^ oidio
 ^KK^ doenças, suspeitas
 ^++^ 040
 Oidio (suspeita)
Veja também:recom_oidio_sus

Para um diagnóstico categórico, verifique se existe um revestimento ralo, branco-acinzentado e pulverulento, aparentando um polvilhamento com cinza vegetal, na face superior das folhas.

^##^ recom_trac_sus
 Recomendações gerais para suspeitas de pragas e doençassuspeita
Detalhes sobre a traça das castanhastraca

^##^ recom_broc_sus
 Recomendações gerais para suspeitas de pragas e doençassuspeita
Detalhes sobre a broca das pontasbroca

^##^ recom_pulg_sus
 Recomendações gerais para suspeitas de pragas e doençassuspeita
Detalhes sobre o pulgão das inflorescênciaspulgao

^##^ recom_erio_sus
 Recomendações gerais para suspeitas de pragas e doençassuspeita
Detalhes sobre o eriofiídeo das floreseriofiideo

^##^ recom_tripes_sus
 Recomendações gerais para suspeitas de pragas e doençassuspeita
Detalhes sobre a tripestripes

^##^ recom_saia_sus
 Recomendações gerais para suspeitas de pragas e doençassuspeita
Detalhes sobre a lagarta saia justasaiajusta

^##^ recom_verde_sus
 Recomendações gerais para suspeitas de pragas e doençassuspeita
Detalhes sobre a lagarta verdeverde

^##^ recom_folhas_sus
 Recomendações gerais para suspeitas de pragas e doençassuspeita
Detalhes sobre a lagarta das folhasfolhas

^##^ recom_noiva_sus
 Recomendações gerais para suspeitas de pragas e doençassuspeita
Detalhes sobre a lagarta véu de noivanoiva

^##^ recom_besouro_sus
 Recomendações gerais para suspeitas de pragas e doençassuspeita
Detalhes sobre o besouro vermelhobesouro

^##^ recom_mosca_sus
 Recomendações gerais para suspeitas de pragas e doençassuspeita
Detalhes sobre a mosca brancamosca

^##^ recom_broto_sus
 Recomendações gerais para suspeitas de pragas e doençassuspeita
Detalhes sobre a larva do broto terminallarva

^##^ recom_cig_sus
 Recomendações gerais para suspeitas de pragas e doençassuspeita
Detalhes sobre a cigarrinhacigarrinha

^##^ recom_antrac_sus
 Recomendações gerais para suspeitas de pragas e doençassuspeita
Detalhes sobre a antracnoseantracnose

^##^ recom_mofo_sus
 Recomendações gerais para suspeitas de pragas e doençassuspeita
Detalhes sobre o mofo pretomofo

^##^ recom_oidio_sus
 Recomendações gerais para suspeitas de pragas e doençassuspeita
Detalhes sobre o oidiooidio
