# Supplementary material for: Implementation of expert systems to support the functional evaluation of stand-to-sit activity
Source: Biomed Eng Online. 2014 Jul 21;13:98. doi: 10.1186/1475-925X-13-98 (PMC4120714; doi:10.1186/1475-925X-13-98)
Supplement: Additional file 2 — Shell Expert Sinta. [file 1475-925X-13-98-S2.zip › sintaFonte.zip!/HELP/SECAJU.ZIP!/TOPICOS2.RTF]

^##^ azinphos_etil 
 ^$$^ azinphos etil
 ^kk^ produtos químicos
 ^++^ 050
 azinphos etil

	Inseticida/acaricida altamente tóxico.
	Intervalo de segurança: 21 dias


NOME COMERCIAL		FORMULAÇÃO E CONCENTRAÇÃO	DOSAGEM
						    (*)			(ml/100 l agua)	

Azincol						CE  40 %		100-200
Guzathion					CE  40 %		100-200

(*) CE concentrado emulsionável

^##^ benomil
 ^$$^ benomil
 ^kk^ produtos químicos ^+^
^+^ 051 benomil

	Fungicida pouco tóxico.
	Intervalo de segurança: 1 dia

NOME COMERCIAL		FORMULAÇÃO E CONCENTRAÇÃO	DOSAGEM
						    (*)			(ml/100 l agua)	

Bentale						PM  50 %		 60-100

(*) PM = pó molhável

^##^ fenitrothion
 ^$$^ fenitrothion
 ^kk^ produtos químicos ^+^
^+^ 052 fenitrothion

	Inseticida/acaricida pouco tóxico.
	Intervalo de segurança: 14 dias

NOME COMERCIAL		FORMULAÇÃO E CONCENTRAÇÃO	DOSAGEM
				  		    (*)			(ml/100 l agua)	
Folithion					CE  50 %		100-200
Sumithion					CE  50 %		100-200

(*) CE  = concentrado emulsionável

^##^ hidro_cobre
 ^$$^ hidróxido de cobre
 ^kk^ produtos químicos ^+^
^+^ 053 hidróxido de cobre

	Fungicida praticamente não-tóxico.
	Intervalo de segurança: 7 dias

NOME COMERCIAL		FORMULAÇÃO E CONCENTRAÇÃO	DOSAGEM
				  		    (*)			(ml/100 l agua)	
Copidrol					PM  45 %		250-300

(*) PM = pó molhável

^##^ malathion
 ^$$^ malathion
 ^kk^ produtos químicos ^+^
^+^ 054 malathion

	Inseticida/acaricida pouco tóxico.
	Intervalo de segurança: 7 dias

NOME COMERCIAL		FORMULAÇÃO E CONCENTRAÇÃO	DOSAGEM
				  		    (*)			(ml/100 l agua)	
Agridion 					CE  50 %		150-200
Malatol 						CE  50 %		150-200

(*) CE  = concentrado emulsionável

^##^ mancozeb
 ^$$^ mancozeb
 ^kk^ produtos químicos ^+^
^+^ 055 mancozeb

	Fungicida pouco tóxico.
	Intervalo de segurança: 21 dias

NOME COMERCIAL		FORMULAÇÃO E CONCENTRAÇÃO	DOSAGEM
				  		    (*)			(ml/100 l agua)	
Dithane M-45					PM  80 %		150-200

(*) PM = pó molhável

^##^ oxi_cobre
 ^$$^ oxicloreto de cobre
 ^kk^ produtos químicos ^+^
^+^ 056
 oxicloreto de cobre

	Fungicida praticamente não-tóxico.
	Intervalo de segurança: 7 dias

NOME COMERCIAL		FORMULAÇÃO E CONCENTRAÇÃO	DOSAGEM
				  		    (*)			(ml/100 l agua)	
Coprantol					SC  52 %		400 ml
Coprantol BR					PM  87 %		200-300 g
Cuproson Azul					PM  35 %		400-500 g
Fluguran					SAq 30 %		300-500 ml
Ramexane					PM  85 %		200-300 g
Super Cupra					PM  50 %		350 g
Agrinose br					PM  60 %		650 g

(*) SC  = suspensão concentrada
    PM  = pó molhável
    Saq = solução aquosa

^##^ cobre_maneb_zineb
 ^$$^ oxicloreto de cobre + maneb + zineb
 ^kk^ produtos químicos ^+^
^+^ 057
 oxicloreto de cobre + maneb + zineb

	Fungicida praticamente não-tóxico.
	Intervalo de segurança: 7 dias

NOME COMERCIAL		FORMULAÇÃO E CONCENTRAÇÃO	DOSAGEM
				  		    (*)			(ml/100 l agua)	
Peprosan					PM  50 %		300-400

(*) PM  = pó molhável

^##^ parathion_etil
 ^$$^ parathion etil
 ^kk^ produtos químicos ^+^
^+^ 058
 parathion etil

	Inseticida/acaricida altamente tóxico.
	Intervalo de segurança: 15 dias

NOME COMERCIAL		FORMULAÇÃO E CONCENTRAÇÃO	DOSAGEM
				  		    (*)			(ml/100 l agua)	
Rhodiatox					CE  60 %		 50-80

(*) CE = concentrado emulsionável

^##^ parathion_methyl
 ^$$^ parathion methyl
 ^kk^ produtos químicos ^+^
^+^ 059
 parathion methyl

	Inseticida/acaricida altamente tóxico.
	Intervalo de segurança: 15 dias

NOME COMERCIAL		FORMULAÇÃO E CONCENTRAÇÃO	DOSAGEM
				  		    (*)			(ml/100 l agua)	
Folidol						CE  60 %		 70-100
Folisuper					CE  60 %		 70-100

(*) CE = concentrado emulsionável

^##^ trichlorfon
 ^$$^ trichlorfon
 ^kk^ produtos químicos ^+^
^+^ 060
 trichlorfon

	Inseticida pouco tóxico.
	Intervalo de segurança: 7 dias

NOME COMERCIAL		FORMULAÇÃO E CONCENTRAÇÃO	DOSAGEM
				  		    (*)			(ml/100 l agua)	
Dipterex					SC  50 %		150-200
Danex						SC  50 %		150-200

(*) SC = suspensao concentrada
