# Supplementary material for: Implementation of expert systems to support the functional evaluation of stand-to-sit activity
Source: Biomed Eng Online. 2014 Jul 21;13:98. doi: 10.1186/1475-925X-13-98 (PMC4120714; doi:10.1186/1475-925X-13-98)
Supplement: Additional file 2 — Shell Expert Sinta. [file 1475-925X-13-98-S2.zip › sintaFonte.zip!/HELP/SECAJU.ZIP!/TOPICOS1.RTF]

^##^ abertura
 ^$$^ como funciona o SECAJU
 ^KK^ SECAJU, utilidade e funcionamento
 ^++^ 001
 Funcionamento do SECAJU

	Este  sistema  reúne,  de  forma  aplicável,  o  conhecimento  de especialistas em pragas e doenças da cultura do cajueiro. Seu objetivo é realizar a diagnose de algum cajueiral afetado e prover as recomendações de  controle  fitossanitário.  A  diagnose  é  feita  em  dois  níveis: categórica e presuntiva. 

	O SECAJU destina-se a produtores de caju,  extensionistas rurais, e pesquisadores e estudantes das áreas de entomologia e fitopatologia.

	As pragaspragas que podem ser diagnosticadas pelo SECAJU são: 
 1. traça das castanhas
 2. broca das pontas
 3. pulgão das inflorescências
 4. eriofiídeo das flores
 5. tripes
 6. lagartas desfolhadoras (saia justa, verde, das folhas e véu de noiva)
 7. besouro vermelho
 8. mosca branca
 9. larva do broto terminal
10. cigarrinha

	Existem outras pragas (ácaros) associadas à cultura do cajueiro que não tem ainda importância relevante comprovada, tais como os tenuipalpídeos, os tetraniquídeos e o eriofiídeo das folhas, os quais só poderão ser vistos com o auxilio de microscópio estereoscópio (lupa) ou lente de aumento.

	Três doençasdoencas também podem ser diagnosticadas:
 1. antracnose
 2. mofo preto
 3. oidio

	Para obter informações sobre COMO USAR O EXPERT SINTA, durante uma consulta, aperte a TECLA F1. Essas teclas estão disponíveis no momento de uma pergunta ou da apresentação de CONCLUSÕES.

	Sugestões e pedidos de informação são muito bem-vindos !

^##^ sobre
 ^$$^ Sobre o SECAJU
 ^KK^ equipe de desenvolvimento ^+^
^+^ 002
 Equipe de desenvolvimento
Projeto conjunto CNPCa - DIN:
. CNPCa - Centro Nacional de Pesquisa de Caju
. DIN   - Departamento de Informática

Equipe responsável pela base de conhecimentos:
. Quélzia Maria Silva Melo 	 (Doutora em Entomologia  / CNPCa)
. Antonio Lindemberg M. Mesquita (Mestre em Entomologia   / CNPCa)
. Lianna Maria Saraiva Teixeira  (Mestre em Fitossanidade / CNPCa)
. Francisco das Chagas O. Freire (Doutor em Fitopatologia / CNPCa)

Equipe responsável pela área de Informática:
. Adroaldo Guimarães Rossetti	 (Mestre em Estatística   / CNPCa)
. Genaro de Azevedo Franca	 (Analista de Sistemas    / DIN)

Primeira versão: Agosto - Outubro / 1988.
Adaptação para o Expert SINTA: Outubro - Novembro / 1995

^##^ bibliografia
 ^$$^ Bibliografia
 ^KK^ bibliografia ^+^
^+^ 003
 Bibliografia
1. EMPRESA BRASILEIRA DE PESQUISA AGROPECUÁRIA. CNPCa. "Cultura de Cajueiro";
      Identificação e controle de pragas e doenças. Fortaleza, EMBRAPA:CNPCa,
      1988. 1p. (Folder)
      
2. MELO, Q. M. S. & CAVALCANTE, R. D.. Pragas. In: LIMA, V. de P. M. S. (org.)
      "A cultura do cajueiro no Nordeste do Brasil". Fortaleza, BNB:
      Escritório Técnico de Estudos Econômicos do Nordeste, 1988. p. 267-300.
      (BNB. Estudos Econômicos e Sociais, 35)
      
3. MELO, Q. M. S. "Eficiência de inseticidas, número e época de aplicações no 
      controle da 'Broca das Pontas' do cajueiro, Anthistarcha binocularis 
      Meyrick, 1929 (Lep., Gelechiidae). Recife, Universidade Federal Rural de
      Pernambuco, 1980. 37p. (Dissertação de mestrado)

4. MELO, Q. M. S.; CAVALCANTE, M. L. S.; MELO, F. I. O. & CAVALCANTE, R. D.
      "Incidência de pragas nos cajueirais do Ceará'". Fortaleza, EPACE, 1979.

5. MELO, Q. M. S. "Pragas do cajueiro". Piracicaba, USP: ESALQ, 1981. 31p.
      (mimeografado)
      
6. TEIXEIRA, L. M. S. "Doenças". In: LIMA, V. de P. M. S. (org.) "A cultura do
      cajueiro no Nordeste do Brasil". Fortaleza, BNB: Escritório Técnico de 
      Estudos Econômicos do Nordeste, 1988. p. 231-66. 
      (BNB. Estudos Econômicos e Sociais, 35)

^##^ pragas
  ^++^ 004
 Pragas Diagnosticáveis pelo SECAJU
	As pragas que podem ser diagnosticadas pelo SECAJU são:

 1.   traça das castanhastraca
 2.   broca das pontasbroca
 3.   pulgão das inflorescênciaspulgao
 4.   eriofiídeo das floreseriofiideo
 5.   tripestripes
 6.   lagarta saia justasaiajusta
 7.   lagarta verdeverde
 8.   lagarta das folhasfolhas
 9.   lagarta véu de noivanoiva
 10. besouro vermelhobesouro
 11. mosca brancamosca
 12. larva do broto terminallarva
 13. cigarrinhacigarrinha

^##^ traca
 ^$$^ traça das castanhas
 ^KK^pragas
 ^++^ 005
 Traça das castanhas
Veja também:recomendacoes

Taxonomia

Anacampsis sp.
Lepidoptera, Gelechiidae

Recomendações de controle

Durante a colheita, todas as castanhas broqueadas (tanto as que estiverem na própria planta, como as que estiverem no chão) deverão ser coletadas, separadas das castanhas sadias e queimadas  fora da área do plantio.

Produtos químicos utilizados

fenitrothionfenitrothion
malathionmalathion


^##^ broca
 ^$$^ broca das pontas
 ^KK^ pragas
 ^++^ 006
 Broca das pontas
Veja também:recomendacoes

Taxonomia

Anthistarcha binocularis
Meyrick, 1929
Lepidoptera, Gelechiidae

Controle preventivo:

Pulverizar:
. Época:		início das épocas de floração e frutificação;
. Modo de Aplicação:	6 pulverizações a intervalos de 10 dias;

Produtos químicos utilizados

fenitrothionfenitrothion
malathionmalathion


^##^ pulgao
 ^$$^ pulgão das inflorescências
 ^KK^ pragas
 ^++^ 007
 Pulgão das inflorescências
Veja também:recomendacoes

Taxonomia

Aphis gossypii
Glover, 1875
Homoptera, Aphididae

Controle preventivo:

Pulverizar:
. Época:		assim que aparecerem os primeiros sintomas da presença do inseto;
. Modo de aplicação:	2 pulverizações com um intervalo de 10 dias.

Produtos químicos utilizados

fenitrothionfenitrothion
parathion methylparathion_methyl
malathionmalathion


^##^ eriofiideo
 ^$$^ eriofiídeo das flores
 ^KK^ pragas
 ^++^ 008
 Eriofiídeo das flores
Veja também:recomendacoes

Taxonomia

Eriophyes rossettonis (?)
Keifer, 1969 
Acarina, Eriophyiidae

Controle preventivo:

Pulverizar:
. Época:		assim que forem observados os primeiros sintomas do ataque do ácaro;
. Modo de aplicação:	2 pulverizações com um intervalo de 10 dias.

Produtos químicos utilizados

fenitrothionfenitrothion
parathion methylparathion_methyl
parathion etilparathion_etil
azinphos etilazinphos_etil
malathionmalathion


^##^ tripes
 ^$$^ tripes
 ^KK^ pragas
 ^++^ 009
 Tripes
Veja também:recomendacoes

Taxonomia

Selenothrips rubrocinctus
Giard, 1901
Thysanoptera, Thripidade

Controle preventivo:

Pulverizar:
. Época:		assim que aparecerem os primeiros sintomas da presença do inseto;
. Modo de aplicação:	2 pulverizações com um intervalo de 10 dias.

Produtos químicos utilizados

fenitrothionfenitrothion
parathion methylparathion_methyl
malathionmalathion


^##^ saiajusta
 ^$$^ lagarta saia justa
 ^KK^ pragas
 ^++^ 010
 Lagarta saia justa
Veja também:recomendacoes

Taxonomia

Cicinnus callipius
Sch., 1928
Lepidoptera, Mimallonidae

Controle preventivo:

Pulverizar:
. Época:		sempre que se constatar ataque dessa praga;
. Modo de aplicação:	2 pulverizações com um intervalo de 10 dias.

Produtos químicos utilizados

fenitrothionfenitrothion
parathion methylparathion_methyl
trichlorfontrichlorfon
azinphos etilazinphos_etil
malathionmalathion


^##^ verde
 ^$$^ lagarta verde
 ^KK^ pragas
 ^++^ 011
 Lagarta verde
Veja também:recomendacoes

Taxonomia

Eacles imperialis magnifica
Walk, 1956
Lepidoptera, Attacidae

Controle preventivo:

Pulverizar:
. Época:		sempre que se constatar ataque dessa praga;
. Modo de aplicação:	2 pulverizações com um intervalo de 10 dias.

Produtos químicos utilizados

fenitrothionfenitrothion
parathion methylparathion_methyl
trichlorfontrichlorfon
azinphos etilazinphos_etil
malathionmalathion


^##^ folhas
 ^$$^ lagarta das folhas
 ^KK^ pragas
 ^++^ 012
 Lagarta das folhas
Veja também:recomendacoes

Taxonomia

Cerodirphia rubripes
Draudt, 1930
Lepidoptera, Hemileucidae

Controle preventivo:

Pulverizar:
. Época:		sempre que se constatar ataque dessa praga;
. Modo de aplicação:	2 pulverizações com um intervalo de 10 dias.

Produtos químicos utilizados

fenitrothionfenitrothion
parathion methylparathion_methyl
trichlorfontrichlorfon
azinphos etilazinphos_etil
malathionmalathion


^##^ noiva
 ^$$^ lagarta véu de noiva
 ^KK^ pragas
 ^++^ 013
 Lagarta véu de noiva
Veja também:recomendacoes

Taxonomia

Thagona sp.
Lepidoptera, Lymantriidae

Controle preventivo:

Pulverizar:
. Época:		sempre que se constatar ataque dessa praga;
. Modo de aplicação:	2 pulverizações com um intervalo de 10 dias.

Produtos químicos utilizados

fenitrothionfenitrothion
parathion methylparathion_methyl
trichlorfontrichlorfon
azinphos etilazinphos_etil
malathionmalathion


^##^ besouro
 ^$$^ besouro vermelho
 ^KK^ pragas
 ^++^ 014
 Besouro vermelho
Veja também:recomendacoes

Taxonomia

Crimissa cruralis
Stal, 1858
Coleoptera, Chrysomelidae

Controle preventivo:

Pulverizar:
. Época:		sempre que se constatar ataque dessa praga;
. Modo de aplicação:	2 pulverizações com um intervalo de 10 dias.

Produtos químicos utilizados

fenitrothionfenitrothion
parathion methylparathion_methyl
trichlorfontrichlorfon
azinphos etilazinphos_etil
malathionmalathion


^##^ mosca
 ^$$^ mosca branca
 ^KK^ pragas
 ^++^ 015
 Mosca branca
Veja também:recomendacoes

Taxonomia

Aleurodicus cocois
Curtis, 1846
Homoptera, Alevrodidae

Controle preventivo:

Pulverizar:
. Época:		assim que aparecerem os primeiros sintomas da presença do inseto;
. Modo de aplicação:	2 pulverizações com um intervalo de 10 dias.

Produtos químicos utilizados

fenitrothionfenitrothion
parathion methylparathion_methyl
malathionmalathion


^##^ larva
 ^$$^ larva do broto terminal
 ^KK^ pragas
 ^++^ 016
 Larva do broto terminal
Veja também:recomendacoes

Taxonomia

Diptera, Cecidomyiidae

Controle preventivo:

Pulverizar:
. Época:		assim que surgirem os primeiros sintomas do ataque da praga;
. Modo de aplicação:	2 pulverizações com um intervalo de 10 dias.

Produtos químicos utilizados

trichlorfontrichlorfon
malathionmalathion


^##^ cigarrinha
 ^$$^ cigarrinha
 ^KK^ pragas
 ^++^ 017
 Cigarrinha
Veja também:recomendacoes

Taxonomia

Homoptera, Cercopidae

Controle preventivo:

Pulverizar:
. Época:		no início do ataque da praga;
. Modo de aplicação:	2 pulverizações com um intervalo de 10 dias.

Produtos químicos utilizados

trichlorfontrichlorfon


^##^ doencas
  ^++^ 018
 Doenças Diagnosticáveis pelo SECAJU
	As doenças que podem ser diagnosticadas pelo SECAJU são:

 1.   antracnoseantracnose
 2.   mofo pretomofo
 3.   oidiooidio


^##^ antracnose
 ^$$^ antracnose
 ^KK^ doenças
 ^++^ 019
 Antracnose
Veja também:recomendacoes

Taxonomia

Glomerella cingulata
(Colletotrichum gloeospoioides)
Penz.
Melanconiales, Melanconiaceae

Controle preventivo:

PODA DE LIMPEZA:

. Época: no fim do período chuvoso e antes do fluxo foliar;
. Eliminar e queimar os ramos mais atacados ou secos;
. Manter os viveiros longe de plantas doentes.

PULVERIZAÇÃO:

. Época:		durante o período de floração e na fase inicial da frutificação;
. Modo de Aplicação:	o número e os intervalos de aplicação são definidos de acordo com o nível de infecção e prevalência de condições climáticas favoráveis à antracnose.

Produtos químicos utilizados

benomilbenomil
hidróxido de cobrehidro_cobre
mancozebmancozeb
oxicloreto de cobreoxi_cobre
oxicloreto de cobre + maneb + zinebcobre_maneb_zineb


^##^ mofo
 ^$$^ mofo preto
 ^KK^ doenças
 ^++^ 020
 Mofo preto
Veja também:recomendacoes

Taxonomia

Perisporiopsella anacardii
(Diploidium anacardiacearum)
Bat. & Caval.
Moniliales, Dematiaceae

Controle preventivo:

PODA DE LIMPEZA:

. Eliminar as partes mais afetadas.

PULVERIZAÇÃO:

. Não se conhece a eficiência de fungicidas específicos.

Produtos químicos utilizados

hidróxido de cobrehidro_cobre
oxicloreto de cobreoxi_cobre
oxicloreto de cobre + maneb + zinebcobre_maneb_zineb


^##^ oidio
 ^$$^ oidio
 ^KK^ doenças
 ^++^ 021
 Oidio
Veja também:recomendacoes

Taxonomia

Oidium anacardii
Noack
Moniliales, Moniliaceae

Controle preventivo:

Como os danos causados tem sido muito pouco significativos, não se tem recomendado controle específico sistemático contra o oidio.

Em casos de ataque muito severo, sobretudo às inflorescências, justifica-se a aplicação de fungicidas à base de enxofre.

^##^ recomendacoes Recomendações gerais para pragas e doençasde_fato

^#^
^#^ de_fato
 ^$$^ recomendações de controle
 ^KK^ recomendações
 ^++^ 090 Recomendações de controle

Poda de limpeza:

Antes de qualquer PULVERIZAÇÃO e antes do início da FLORAÇÃO, os cajueiros deverão receber uma poda de limpeza, com a finalidade de uniformizar o seu estado sanitário. Esse cuidado, no entanto, é praticamente inviável em grandes plantios.

Dosagem do produto químico:

Em cada recomendação de produto, a dosagem está fixada para 100 litros de água, isto é, aplicação a ALTO VOLUME. Nesses casos, o consumo da calda varia de 500 a 1000 litros por hectare e a pulverização deve cobrir uniformemente a copa das plantas até o inicio do escorrimento.

Para aplicações a BAIXO VOLUME, a dosagem do produto deve ser aumentada proporcionalmente à redução do gasto de água. Nesses casos, o consumo da calda varia de 80 a 120 litros por hectare e as gotas devem cair sobre as folhas sem necessidade de molhá-las excessivamente.

Meios de aplicação:

PULVERIZADOR COSTAL, manual ou motorizado, para plantas jovens (até 4 anos de idade).

ATOMIZADOR, acionado por trator, para plantios maiores, mesmo com plantas de pequeno e médio porte.

VIA AÉREA, a partir do 7o.-8o. ano de cultivo (quando as copas dos cajueiros estão bastante desenvolvidas) e para grandes plantios (independentemente do porte das plantas).

Intervalo de segurança:

É a quantidade de dias que devem decorrer desde a última aplicação do defensivo até o início da colheita. Esse intervalo serve para assegurar que os resíduos do produto não ultrapassem os limites máximos permitidos.

CUIDADO: TÓXICO !!

- Trata-se de substâncias tóxicas que requerem cuidados especiais para seu manuseio.
- Leia atentamente as instruções do rótulo do produto !
- Adote as devidas medidas de segurança para proteger o aplicador, o meio ambiente e o consumidor !
- Respeite o intervalo de segurança anterior `a colheita !

^#^
^#^ suspeita
 ^$$^ recomendações sobre suspeitas
 ^kk^ recomendações
 ^++^ 091
 Recomendações sobre suspeitas

Diagnóstico presuntivo:

Essas pragas ou doenças não puderam ser diagnosticadas de maneira categórica porque as informações fornecidas ao sistema não foram suficientes. De qualquer forma, há alguma evidência da ocorrência dessas pragas ou doenças.

Para que possa ser feito um diagnóstico seguro, sugerimos que sejam verificadas as características chaves para cada uma das pragas ou doenças suspeitas, segundo as recomendações disponíveis.

Em caso de dúvidas, sugerimos que o material seja coletado e enviado ao CNPCa (Centro Nacional de Pesquisa de Caju - Fortaleza, CE) e/ou ao escritório local da EMATER (Empresa de Assistência Técnica e Extensão Rural).

Cuidados na coleta do material:

- Retirar as partes da planta ATACADAS por pragas ou doenças;
- Acondicioná-las, SEPARADAMENTE, em saco de papel;
- Colocar o saco de papel, contendo o material, dentro de um saco plástico;
- EVITE a coleta de partes da planta já mortas (totalmente secas);
- Enviar o material no menor espaço de TEMPO possível. Se for haver demora, armazenar em REFRIGERADOR.
- Incluir INFORMAÇÕES COMPLEMENTARES sobre o material, tais como: local e data de coleta, nome e endereço do interessado, tipo de sintoma ou dano causado, partes da planta afetadas, se o problema ocorreu em anos anteriores, e informações gerais sobre as condições climáticas ocorrentes no período imediatamente anterior ao ataque da praga ou doença;
- No caso de INSETOS não tão pequenos (lagartas, besouros, percevejos etc.), enviá-los em recipiente com álcool, hermeticamente fechado.
