# Supplementary material for: Implementation of expert systems to support the functional evaluation of stand-to-sit activity
Source: Biomed Eng Online. 2014 Jul 21;13:98. doi: 10.1186/1475-925X-13-98 (PMC4120714; doi:10.1186/1475-925X-13-98)
Supplement: Additional file 2 — Shell Expert Sinta. [file 1475-925X-13-98-S2.zip › sintaFonte.zip!/HELP/SECAJU.ZIP!/CONTENTS.RTF]

Funcionamento do SECAJUabertura
Equipe de desenvolvimentosobre
Bibliografiabibliografia
Pragas diagnosticáveispragas
Doenças diagnosticáveisdoencas
