# Supplementary material for: Implementation of expert systems to support the functional evaluation of stand-to-sit activity
Source: Biomed Eng Online. 2014 Jul 21;13:98. doi: 10.1186/1475-925X-13-98 (PMC4120714; doi:10.1186/1475-925X-13-98)
Supplement: Additional file 2 — Shell Expert Sinta. [file 1475-925X-13-98-S2.zip › sintaFonte.zip!/3DPARTY/TB97_147.ZIP!/TB97.DOC]

**Toolbar97**

Version 1.47

By Jordan Russell

email:	jordanr7@aol.com

www:	http://members.aol.com/jordanr7

Toolbar97 is a free dockable toolbar component for Delphi 1.0, 2.0, 3.0, and C++Builder that features the new Office 97 look and behavior. Some of its features include:

- Ability to drag and dock toolbars to any side of a form, or leave them floating. Multiple toolbars can lined up side-by-side or in rows.
- Full source code for customization if necessary.
- Includes a *TToolbarButton97* control that looks and works just like the buttons in Office 97.
- Includes a *TEdit97* control for creating Office 97-style edit controls on toolbars.
- Ability to save and load its position from the registry (see below).
- Doesn't require the new COMCTL32.DLL to run, as Delphi 3's TToolBar (sometimes) and TCoolBar components do.
- Tested and certified to work on Windows 95, NT 4.0, and 3.51 (16/32-bit), and Windows 3.1 (16-bit only).

This version does not include *all* of the functionality and behavior of the Office 97 toolbars yet. I am working on a v2.0 that should include these features:

- Office 97-style *menus*!
- Run-time toolbar customization.
- Dockable windows (i.e. not toolbars)
- An Office 97-style toolbar combo box.
- Arrows on toolbars that go off the screen.
- Smooth window dragging support, like in Office 97 with Windows NT or the *Plus!* pack installed.

**How to Use**

**IMPORTANT:** You must unzip the Toolbar97 package with the *-d* parameter of PKZIP. Or if you're using WinZip, you must check *Use Folder Names*.

First you need to install the component in Delphi or C++Builder. Click *Component | Install,* then the *Add* button, and locate the TB97.PAS file.

You might want to take a look at the DEMO (or DEMOCB for C++Builder) project to see a demonstration of Toolbar97's capabilities. **Note:** The DEMO project will not load in Delphi 1 (sorry!).

The Toolbar97 package includes five components: TDock97, TToolbar97, TToolbarButton97, TToolbarSep97, and TEdit97. Please read the following sections for important details on each.

**TB97 Globals**

**Functions:**

- procedure IniLoadToolbarPositions (const Form: TForm; const Filename: String);

Loads the positions of all toolbars owned by Form to the .INI file specified by Filename. Only 16-bit applications need to use this — 32-bit applications should load and save to the registry. This should be called when your application starts (usually in the OnCreate handler of your form). If they were not previously saved in the registry, IniLoadToolbarPositions has no effect. Each toolbar's data is loaded from a section whose name is the Name property of the toolbar.

- procedure IniSaveToolbarPositions (const Form: TForm; const Filename: String);

Saves the positions of all toolbars owned by Form to the .INI file specified by Filename. Only 16-bit applications need to use this — 32-bit applications should load and save to the registry. This should be called when your application exits (usually in the OnDestroy handler of your form). Each toolbar's data is saved to a section whose name is the Name property of the toolbar.

- procedure RegLoadToolbarPositions (const Form: TForm; const BaseRegistryKey: String);

Loads the positions of all toolbars owned by Form from the registry. This should be called when your application starts (usually in the OnCreate handler of your form). If they were not previously saved in the registry, RegLoadToolbarPositions has no effect. BaseRegistryKey is the name of the key it loads the data from. LoadPosition will append the Name of the toolbars onto this. For example, if BaseRegistryKey is *SOFTWARE\My Company\My Program\Toolbars* and the Name of a toolbar is *MyToolbar*, it loads the data from the *SOFTWARE\My Company\My Program\Toolbars\MyToolbar* key.

- procedure RegSaveToolbarPositions (const Form: TForm; const BaseRegistryKey: String);

Saves the positions of all toolbars owned by Form to the registry. This should be called when your application exits (usually in the OnDestroy handler of your form). BaseRegistryKey is the name of the key it saves the data to. SavePosition will append the Name of the toolbars onto this. For example, if BaseRegistryKey is *SOFTWARE\My Company\My Program\Toolbars* and the Name of a toolbar is *MyToolbar*, it saves the data from the *SOFTWARE\My Company\My Program\Toolbars\MyToolbar* key.

**TDock97 Reference**

**Description:**

Create TDock97 controls at locations you want a TToolbar97 to be able to dock at. These automatically resize as toolbars are docked onto them. Be sure to set the Position property to designate which side of the form the dock is to be located.

**Key Properties:**

- property Background: TBitmap;

A background bitmap, which is optional. It is tiled across the length of the dock, and any docked toolbars used it also.

- property BackgroundTransparent: Boolean default False;

When True, the color of the bottom-left pixel of the Background bitmap is considered transparent, and is replaced by value of the Color property.

- property BoundLines: TDockBoundLines;

TDockBoundLines = set of (blTop, blBottom, blLeft, blRight);

Use this to add extra lines to the sides of the dock. For docks Positioned at the top of the form, it looks best if you set this to [blTop].

- property FixAlign: Boolean default False;

If at run-time you notice a dock not appearing in the location it should, enabling this should correct the problem. This problem only occurs when you have a TDock97 and another control with the same Align setting (i.e. a toolbar and a list both set to alLeft). When True, this adds an extra pixel to the width or height so that the VCL is able to align it correctly.

- property LimitToOneRow: Boolean default False;

Set this to True if you want to prevent the user from having more than one row of docked toolbars. I generally don't recommend you enable this (since Office 97 doesn't do this), unless you have a fixed-size form that would look wrong with too many rows of toolbars.

- property Position: TDockPosition;

TDockPosition = (dpTop, dpBottom, dpLeft, dpRight);

Determines where the dock is located on the form.

**Events:**

- property OnInsertRemoveBar: TInsertRemoveEvent;

```
TInsertRemoveEvent = procedure (Sender: TObject; Inserting: Boolean;
  Bar: TToolbar97) of object;
```

Occurs after a toolbar is docked (Inserting = True) or undocked (Inserting = False).

**TToolbar97 Reference**

**Description:**

This is the toolbar control itself. You can insert TToolbarButton97 controls or any other controls on this, which it automatically lines up. To create separators, use the TToolbarSep97 component.

**Remarks:**

When the CloseButton property is True (the default), the toolbar can hide itself if the user clicks the close button on a floating toolbar. Because of this, you should always include an item on a menu that toggles the Visible property so the user can get it back if it is closed. See the demo application source code for an example of this.

Toolbars have to recreate themselves whenever they changed from a docked to a floating state and vice versa. Because of this you may notice some types of controls lose some of their values when the toolbar is docked or undocked. If you notice this happening, you need to respond to the OnRecreating and OnRecreated events. Put code in the OnRecreating event that saves the state of the controls in the toolbar, and put code in the OnRecreated event that restores the state. For example, if you had a TComboBox dropdown list, you could make it save its ItemIndex value into a temporary variable in OnRecreating. OnRecreated could then restore the ItemIndex value from the temporary variable.

**Key Properties:**

- property CanDockLeftRight: Boolean default True;

When True, the toolbar can be docked to a TDock97 with a Position of dpLeft or dpRight.

- property Caption;

What appears in the title bar of a floating toolbar.

- property CloseButton: Boolean default True;

When True, a close button appears in the title bar when the toolbar is floating.

- property DefaultDock: TDock97;

The default dock location. This is used when the user double-clicks a floating toolbar. If this is not set, nothing will happen.

- property DockedTo: TDock97;

The TDock97 control that the toolbar is currently docked to. You can assign to this value any TDock97 control that belongs to the same form as the toolbar. To undock a toolbar at design time, delete the value from this property. To undock a toolbar at run time, assign **nil** to this property.

- property DockPos: Integer;

This is only valid if the toolbar is currently docked (DockedTo <> nil). This is its current horizontal (or vertical, if docked to a left or right dock) position in pixels.

- property DockRow: Integer;

This is only valid if the toolbar is currently docked (DockedTo <> nil). This is the row the toolbar is currently docked at.

**Events:**

- property OnDockChanged: TNotifyEvent;

Occurs after the toolbar has changed between docks, or from a docked to floating state or vice versa.

- property OnDockChanging: TNotifyEvent;

Occurs immediately before the toolbar changes between docks, or from a docked to floating state or vice versa.

- property OnRecreated: TNotifyEvent;

Occurs immediately after the toolbar recreates itself. This usually happens when it changes between a docked and non-docked state. See the *Remarks* above for more information.

- property OnRecreating: TNotifyEvent;

Occurs immediately before the toolbar recreates itself. This usually happens when it changes between a docked and non-docked state. See the *Remarks* above for more information.

**Key Methods:**

- procedure SetSlaveControl (const ATopBottom, ALeftRight: TControl);

Call this when the form is created to designate a top/bottom docked and left/right docked version of a control on the toolbar. At design time, create both versions side-by-side (with no separator in between). See the demo application source code for an example of how to use this.

**TToolbarButton97 Reference**

**Description:**

The TToolbarButton97 component is just like Delphi 3's TSpeedButton component, but it works more like Office 97 and adds some new features. You aren't required to use this for the buttons on toolbar, but it's recommended that you do so.

If you want your application to look just like Office 97, you should leave the width of buttons at 23 pixels and the height at 22 pixels. Glyphs should be 16x16 pixels, or 17x17 for some disabled glyphs.

**See the help for the TSpeedButton control for help on the properties, methods, and events not listed here.**

**Key Properties:**

- property DisplayMode: TButtonDisplayMode read FDisplayMode write SetDisplayMode default dmBoth;

TButtonDisplayMode = (dmBoth, dmGlyphOnly, dmTextOnly);

Determines whether the glyph, caption, or both are drawn on the button. You could adjust this if you wanted to, for example, hide glyphs without deleting them.

- property DropdownArrow: Boolean default True;

When True, the button displays an arrow if DropdownMenu is assigned.

- property DropdownMenu: TPopupMenu;

When this is assigned, the button will display this popup menu instead of calling the OnClick handler when clicked. The menu appears below the button, or to the right if the parent toolbar is docked to the left or right side.

- property Opaque: Boolean default True;

When True, the button is not transparent, which prevents the "blinking" effect that you see when you move the mouse over it or click it (as seen in Delphi 3's TSpeedButton, and most other button components). You should only need to set this to False if you have a Background for the dock.

- property WordWrap: Boolean default False;

When True, text that is too wide for the button is wrapped into several lines.

**TToolbarSep97 Reference**

**Description:**

The TToolbarSep97 component is used to create separators between toolbar buttons. It automatically adjusts its size and orientation at design and run time. At run time, it also adjusts its visibility when necessary.

**TEdit97 Reference**

**Description:**

The TEdit97 component is just like Delphi's TEdit component, except it has the Office 97 appearance, and is missing a few properties that are not applicable.

**Revision History**

1.47

- Rewrote most of moving and resizing routines to make it work exactly like Office 97.
- Added a DisplayMode property to TToolbarButton97.
- Toolbars now work flawlessly on MDI child forms.
- A few other minor improvements and fixes.

1.46

- Added a LimitToOneRow property to TDock97.
- C++Builder transparency problems in 1.45 fixed.
- A minor disabled glyph transparency problem that affected Delphi 2 and C++Builder fixed.
- Some minor improvements.

1.45

- Dropdown menus now fully supported on TToolbarButton97.
- Backgrounds on TDock97's now supported. See Background property.
- Added a FixAlign property to TDock97.
- Added a WordWrap property to TToolbarButton97.
- The problems in 1.4 fixed.
- A *lot* of little problems that have been around since 1.3 fixed (see source code for details).

1.4

- Now supports Delphi 1 also.
- Loading and saving toolbars' positions is now done with procedures called RegLoadToolbarPositions and RegSaveToolbarPositions for loading and saving to the registry, and IniLoadToolbarPositions and IniSaveToolbarPositions for loading and saving to .INI files. This corrects a problem that sometimes caused toolbars to be loaded incorrectly.
- Fixed minor flickering problem while dragging.
- Now prevents you from dropping a toolbar under the taskbar (32-bit only).
- Several minor, but annoying, problems fixed. See source code for details.
- A lot of tweaks to the source code.

1.37

- Fixed problem of floating toolbars sometimes reappearing (without the program telling them to) after you closed them.

1.36

- Fixed a couple of very minor and insignificant cosmetic problems. See the source code for details.

1.35

- Fixes all known problems, including the rather serious problem in TToolbarButton97 of it sometimes displaying the wrong glyphs in Delphi 2.
- Several more problems fixed. See the source code for details.

1.33

- Added a TEdit97 component.
- Fixed a lot of minor but noteworthy problems. See the source code for details.

1.32

- Fixed another minor problem in TToolbar97 that caused it to sometimes incorrectly reorder the controls.

1.31

- Fixed problems with loading the demo application.
- Fixed a very minor problem in TToolbarButton97.

1.3

- Floating toolbars are now resizable. Like Office 97, they also now hide themselves when the application is deactivated.
- Separators are no longer automatically created. You now create them using the TToolbarSep97 component.
- Added a CanDockLeftRight property to TToolbar97.
- Added an Opaque property to TToolbarButton97. Also made the buttons now stay down until it returns from the OnClick handler, like Office 97.
- A *lot* of small fixes on all of the controls (too many to mention) to make them work more like Office 97.
- C++Builder now supported.

1.2

- Left and right side docking now supported. A SetSlaveControl method added to TToolbar97 for designating top/bottom docked and left/right docked versions of controls on the toolbar.
- A lot of little problems fixed.

1.11

- Added a CloseButton property to TToolbar97.
- More bug fixes.

1.1

- Delphi 3 now supported!
- TToolbarButton97 component completely redesigned. Now it works exactly like a TSpeedButton. I also borrowed some of the glyph caching techniques used in Delphi 3 to make it much faster.
- Added a Color property to the TDock97 and TToolbar97 controls so it now looks right in MDI forms.
- TToolbar97's BarHeight property is no longer global to the whole application, as this sometimes caused problems. Now each toolbar has an individual BarHeight setting, which allows for greater flexibility.
- Many design mode problems fixed. (And you can now move docked toolbars!)

1.0

- First release.
